# Global and China trends in glomerulonephritis-induced chronic kidney disease: health inequities, risk factors and projections to 2050

**DOI:** 10.1080/0886022X.2025.2564373

**Published:** 2025-10-15

**Authors:** Mengting Wang, Zhengyu Hu, Lu Yang, Tao Yang, Hui Yang, Weijin Zhang, Lu Li, Rui Chu, Na Tian

**Affiliations:** ^a^ Department of Nephrology, General Hospital of Ningxia Medical University, School of Ningxia Medical University, Yinchuan, Ningxia, People’s Republic of China; ^b^ Department of Nephrology, General Hospital of Ningxia Medical University, Yinchuan, Ningxia, People’s Republic of China

**Keywords:** Chronic kidney disease, glomerulonephritis, health inequality, global burden of disease, Bayesian age-period-cohort analysis, epidemiological changes

## Abstract

**Background:**

Glomerulonephritis-induced chronic kidney disease (CKD) imposes heavy global health and socioeconomic burdens, but regional vs. global data are limited. We assessed its burden, inequalities, projections in China and globally (1990–2021) to inform prevention strategies.

**Methods:**

Using 2021 Global Burden of Disease data, we analyzed glomerulonephritis-induced CKD indicators: prevalence, incidence, mortality, and disability-adjusted life years. We used advanced methods, including age-period-cohort analysis, decomposition analysis, and Bayesian forecasting to assess temporal trends and health disparities.

**Results:**

From 1990 to 2021, global age-standardized rates (ASRs) of prevalence, incidence, mortality, and DALYs for glomerulonephritis-induced CKD increased. Global age-standardized prevalence (ASPR) rose from 128.55 to 129.94 per 100,000 (1.09%, 95% UI: −0.78% to 2.90%), while China’s ASPR fell from 109.57 to 94.21 (−14.02%, 95% UI: −17.31% to −10.29%). Global slope index of inequality and concentration index decreased. In China, all ASRs decreased. Sex and age disparities were evident, with higher mortality observed among older populations. Decomposition analysis revealed population aging and growth as key contributors to increased mortality. Although a continued reduction in China’s overall disease burden has been projected for 2050, an increasing incidence among individuals over 45 years was observed. Impaired kidney function, hyperglycemia, high body mass index, and hypertension were identified as major risk factors both globally and in China.

**Conclusion:**

Glomerulonephritis-induced CKD remains a considerable global burden, particularly in lower Sociodemographic Index countries. Pronounced age and sex disparities underscore urgency of targeted interventions for high-risk populations in China.

## Introduction

Chronic kidney disease (CKD) is a significant global health threat, affecting an estimated 8% to 16% of the global population [1]. It is a primary contributor to both mortality and disability [2], and is characterized by a progressive decline in glomerular function and elevated urinary albumin levels [3]. The 2021 Global Burden of Disease (GBD) study data revealed that the number of patients with CKD worldwide reached nearly 700 million, ranking 12th among all diseases [4]. Alarmingly, CKD is projected to become the fifth leading cause of death by 2040 [5], driven by rising incidence and mortality [6]. Beyond its direct health impacts, CKD exacerbates cardiovascular disease risks, imposing substantial socioeconomic burdens on healthcare systems [7]. Although CKD encompasses diverse etiologies, glomerulonephritis-induced CKD remains one of the most common causes epidemiologically, particularly in low- and middle-income countries [8–10].

Glomerulonephritis-induced CKD comprises a spectrum of immune-mediated disorders characterized by glomerular structural damage [11]. Clinical manifestations include proteinuria, hematuria, hypertension, and reduced glomerular filtration rate (GFR) [3,8]. Due to the clinically silent nature of early-stage pathogenesis, the disease often delays diagnosis. It typically follows an irreversible progression trajectory. By the time overt symptoms prompt medical evaluation, the disease has often progressed to advanced stages [12–14], frequently culminating in renal replacement therapy, such as dialysis or kidney transplantation. Studies have reported significant regional and population-level differences in glomerulonephritis-induced CKD burden. For example, the 2019 GBD report identified the highest burden in low-middle Sociodemographic Index (SDI) countries (age-standardized prevalence rate, ASPR: 150.19/100,000 vs 107.56/100,000 in high-SDI regions) [15]. This geographical gradient is particularly striking in Asia, where the elevated burden may be correlates with the high prevalence of IgA nephropathy (IgAN) – the most prevalent subtype of glomerulonephritis globally. IgAN exhibits marked epidemiological heterogeneity, with prevalence rates of 47.4% in Japan, 45% in China, and 16.7% in India, contrasting sharply with the 9.4% prevalence observed in the United States [16,17].

China’s epidemiological profile represents a critical paradigm for investigating glomerulonephritis-induced CKD. With a national CKD prevalence of 10.6%, China harbors the world’s largest CKD patient population [2]. Epidemiological surveillance reveals that glomerulonephritis-induced CKD accounts for 55.3% of end-stage kidney disease (ESKD) cases [18 – a proportion significantly higher than that in Western countries such as the United States (16% ESKD attribution) [19]. In 2019, glomerulonephritis caused over 3 million CKD cases in China, ranking second globally [15]. With advancing age, renal function gradually declines, thereby decreasing the GFR [20,21]. Among individuals aged 45 years and older, 11.5% exhibit an estimated glomerular filtration rate (eGFR) < 60 mL/min/1.73 m^2^, affecting approximately 4.3 million individuals. However, fewer than 10% are diagnosed and less than 5% are treated with guideline-directed pharmacotherapy [22]. Compounding this crisis, mortality and disability-adjusted life years (DALYs) attributable to glomerulonephritis-induced CKD peak dramatically in individuals aged 80 years and above [23], identifying advanced age as the predominant risk factor for terminal renal outcomes. Thus, the pressing requirement exists to create screening and therapeutic interventions customized to the specific profiles of diverse populations, thereby reducing the impact of disease. Research on the glomerulonephritis-induced CKD burden in China, particularly among different time periods, sex, and age groups, remains limited.

This study utilized data from the GBD 2021 study, and for the first time employed the age-period-cohort (APC) model and decomposition analysis to systematically compare disparities in the disease burden of glomerulonephritis-induced CKD across different ages, sexes, and temporal trends between global and Chinese populations. It further quantified the relative contributions of population growth, aging, and epidemiological transitions to this burden, thereby providing critical evidence-based support for addressing existing research gaps and developing more targeted prevention and control strategies.

## Materials and methods

### Data sources

Data were obtained from the GBD 2021 database (https://ghdx.healthdata.org/gbd-results-tool), developed by the Institute for Health Metrics and Evaluation (IHME). This database synthesizes cohort studies, national health surveys, vital statistics registries, and hospital records to provide comprehensive estimates for 371 diseases and injuries across 204 countries and regions from 1990 to 2021 [24,25]. We extracted data on glomerulonephritis-induced CKD, including incidence, prevalence, mortality, DALYs, years lived with disability (YLDs) and years of life lost (YLLs), both globally and in China. DALYs were computed as the sum of YLDs and YLLs [4]. We also obtained SDI metrics to examine correlations with socioeconomic development levels. Due to the lack of globally standardized data on specific glomerular disease subtypes, glomerulonephritis-induced CKD was operationally defined using International Classification of Diseases (ICD)-10 codes (N02–N06.9), covering glomerular disorders with morphological alterations, specifically including recurrent/persistent hematuria, chronic nephritic syndrome, nephrotic syndrome, unspecified nephritic syndrome, and isolated proteinuria with documented morphological lesions [15]. This classification approach may group subtypes with distinct clinical characteristics, thereby affecting the specificity and generalizability of the results. Detailed code specifications are provided in Supplementary Table 1. Because the database is publicly available and anonymized, no ethical approval or informed consent was necessary.

### Disease burden indicators

Age-standardized rates (ASRs) for prevalence (ASPR), incidence (ASIR), death (ASDR), DALYs, YLDs, and YLLs were calculated using the global standard population obtained from the GBD 2021 to ensure comparability across regions and time. Data were stratified by sex to explore sex-specific differences in disease burden.

### Trend analysis

Temporal trends of ASRs from 1990 to 2021 were evaluated using the estimated annual percentage change (EAPC) and its 95% uncertainty interval (UI), calculated through linear regression. EAPC allows for standardized comparisons of disease indicators over specific periods [26].

### Age-period-cohort (APC) model

An age-period-cohort (APC) model based on a Poisson distribution was applied to analyze the contributions of age, period, and cohort effects on the incidence and mortality rates of CKD [27]. The analysis was performed using the APC analysis tool (https://analysistools.cancer.gov/apc/) [28].

### Attributable risk analysis

The comparative risk assessment (CRA) methodology was applied to quantify population-attributable disease burden [29]. Risk factors for glomerulonephritis-induced CKD include a diet high in processed meat, red meat, sodium, and sugar-sweetened beverages, as well as low intake of fruits, vegetables, and whole grains, high body mass index (BMI), high fasting plasma glucose, high systolic blood pressure, high and low temperatures, kidney dysfunction, lead exposure, and low physical activity. Detailed definitions of these 15 risk factors are available in the relevant articles on the GBD 2021 risk factors [25]. We used ASDR and ASR of DALYs to assess the disease burden attributable to these factors.

### Decomposition analysis

Leveraging the Das Gupta decomposition technique, we dissected temporal changes in CKD incidence and mortality burden (1990–2021) into proportional contributions from three drivers: population aging, demographic expansion, and epidemiological shifts [30]. This method systematically partitions observed disease burden variations into three distinct components: Aging (Age-structure change): calculated by holding age-specific incidence rates and total population constant while varying age-group proportions between 1990 and 2021. Population growth: derived by fixing age-structure and incidence rates while varying total population size. Epidemiological change: computed by holding age-structure and population size constant while varying age-specific incidence rates.

### Bayesian age-period-cohort (BAPC) model

To project future trends of glomerulonephritis-induced CKD burden in China from 2021 to 2050, we implemented a Bayesian Age-Period-Cohort (BAPC) model with integrated nested Laplace approximation (INLA). This model integrates age, period, and cohort effects to forecast ASPR, ASIR, ASDR, and age-standardized DALY rates, leveraging both sample data and prior information to enhance parameter estimation – a validated approach for modeling disease burden trends [31]. Here, let *a* = 1,…, *A* index age groups (20 strata: 0–4, 5–9, …, 95+ *years*), *p* = 1,…,*P* denote observation periods (1990–2021, 32 intervals), and *c = (A − a) + p* represent birth cohorts. The log-linear model is specified as: log(*Y_ap_)* = *μ + α_a_ + β_p_ + γ_c_ + ϵ_ap_*, where *Y_ap_* represents the age-standardized rate for age group *a* in period *p*, *μ* is the intercept, *α_a_*, *β_p_*, and *γ_c_* denote age, period, and cohort effects, and *ϵ_ap_*∼*N(*0,*σ^−^*^2^*)* captures random noise. Smoothing Priors: Age Effect (*α_a_*): A second-order random walk (RW2) prior enforces smoothness: Δ^2^*α_a_*=*α_a_*−2*α_a−_*_1_+*α_a−_*_2_∼N(0,*τ_α_*^−1^), Period (*β_p_*) and Cohort (*γ_c_*_)_ Effects: Similarly constrained by RW2 priors: Δ^2^*β_p_* ∼N(0,*τ_β_*^−1^), Δ^2^*γ_c_*∼N(0,*τ_γ_*^−1^). Precision parameters *τ_α_*, *τ_β_*, *τ_γ_* were assigned Gamma(1, 0.01) hyperpriors to balance flexibility and regularization. Implementation: Future rates *Y_a,p+t_* (2022–2050) were projected by extrapolating period effects *β_p+t_* while holding age (*α_a_*) and cohort (*γ_c_*) effects constant. All analyses were performed in R 4.3.3 using the BAPC package (v0.0.36) and INLA (v23.04.24), which leverages Laplace approximations for efficient Bayesian computation.

### Health inequality

The Slope Index of Inequality (SII) and Concentration Index (CI) [32] were applied to SDI rankings to evaluate both absolute and relative health inequities. Positive CI values indicated disproportionate DALYs concentration in high-SDI nations, whereas negative coefficients demonstrated a reversal of this pattern. Larger absolute magnitudes for both indices corresponded to heightened health disparities [33].

Data were analyzed and visualized using R (version 4.3.3) and INLA (v23.04.24). Results are presented as means with 95% uncertainty intervals (UIs) or CIs, with significance set at *p* < 0.05.

## Results

### Disease burden of glomerulonephritis-induced CKD in China in 2021

**Figure 1.**
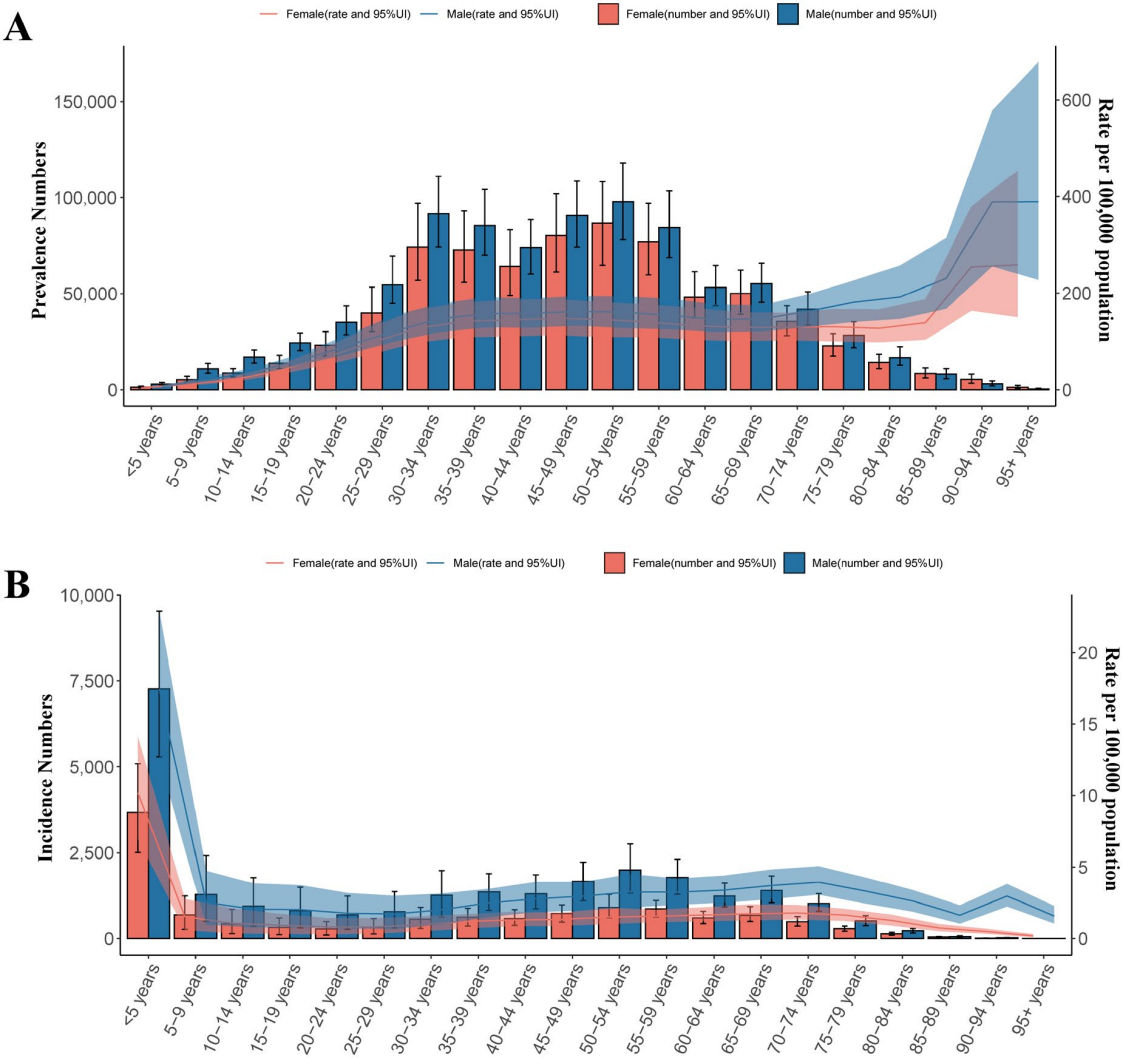
Numbers and ASPR, ASIR for glomerulonephritis-induced CKD in China, 2021. (A) The number of prevalence cases and ASPR by age and sex. (B) The number of incidence cases and ASIR by age and sex. ASPR: age-standardized prevalence rate; ASIR: age-standardized incidence rate.

In 2021, the estimated number of prevalent cases of glomerulonephritis-induced CKD in China was 1.61 million (95% UI: 1.42 million, 1.80 million), reflecting a 28.47% increase from 1990. Among these, 876,650 cases were male and 733,638 were female. The ASPR was notably higher in males (102.94/100,000; 95% UI: 93.51, 112.64) compared to females (85.37/100,000; 95% UI: 72.91, 98.94). Overall, the ASPR was 94.21/100,000 (95% UI: 83.71, 104.86) (Table 1). Among males, the highest number of prevalent cases was observed in the 50 to 54-year age group, with a marked increase in ASPR after the age of 80 years (Figure 1(A)). In addition, sex disparities were evident in the incidence and mortality. In 2021, 37,892.75 new cases were reported (95% UI: 33,122.34, 43,563.66), with ASIR of 4.26/100,000 for males (95% UI: 3.57, 4.99) and 2.27/100,000 for females (95% UI: 1.79, 2.80), resulting in an overall ASIR of 3.31/100,000 (95% UI: 2.71, 3.95) (Table 1). The highest incidence number occurred in the 0 to 4-year age group, with an age-specific decline in ASIR (Figure 1(B)). Mortality data revealed 5,320 deaths from glomerulonephritis-induced CKD, with ASDR of 0.36/100,000 and 0.25/100,000 for males (95% UI: 0.24, 0.50) and females (95% UI: 0.17, 0.35), respectively, resulting in an overall ASDR of 0.30/100,000 (95% UI: 0.22, 0.39) (Table 1). The number of deaths was concentrated in the 65–89 age range, peaking at 70 to 74 years, with a significant elevation in ASDR after the age of 60 years (Supplementary Figure 1A).

**Table 1.** The number of cases of glomerulonephritis-induced CKD across all age groups, ASRs of incidence, prevalence, deaths, DALYs, YLDs, YLLs, and corresponding EAPC in the total population of China and globally in 1990 and 2021.

		1990		2021		1990–2021	
Location	Measure	Cases	ASR per 100,000	Cases	ASR per 100,000	Percentage change	EAPC
		No. (95% UI)	No. (95% UI)	No. (95% UI)	No. (95% UI)	No. (95% UI)	No.(95% UI)
Global	Prevalence	6,370,882.29 (5,926,722.83, 6,848,154.07)	128.55 (119.33, 137.58)	10,735,809.09 (9,925,500.14, 11,520,170.78)	129.94 (120.25, 139.51)	1.09(−0.78, 2.90)	0.06(0.04, 0.08)
	Incidence	244,229.23 (218,810.80, 271,856.94)	4.30 (3.88, 4.75)	357,288.00 (329,226.02, 388,483.48)	4.84 (4.42, 5.29)	12.70(8.12, 17.94)	0.39(0.36, 0.43)
	Death	83,169.75 (69,625.31, 97,737.53)	2.02 (1.68, 2.38)	193,997.40 (162,332.32, 226,569.35)	2.34 (1.96, 2.74)	16.10(6.83, 24.83)	0.54(0.50, 0.59)
	DALYs	3,751,088.42 (3,252,292.04, 4,269,460.41)	77.78 (67.62, 88.41)	6,959,757.64 (6,018,414.33, 7,961,672.88)	84.47 (73.20, 96.13)	8.60(0.46, 17.20)	0.28(0.25, 0.31)
	YLDs	519,467.50 (347,734.12, 690,714.51)	11.34 (7.55, 14.94)	1,021,806.94 (688,294.09, 1,354,551.52)	12.20 (8.24, 16.15)	7.56(3.43, 11.71)	0.35(0.25, 0.44)
	YLLs	3,231,620.92 (2,773,120.23, 3,726,870.91)	66.44 (57.21, 76.80)	5,937,950.71 (5,092,348.25, 6,921,329.79)	72.27 (62.22, 84.18)	8.78(−0.50, 18.50)	0.27(0.23, 0.31)
China	Prevalence	1,253,432.50 (1,126,822.99, 1,398,840.86)	109.57 (98.58, 121.60)	1,610,288.47 (1,424,197.47, 1,795,854.07)	94.21 (83.71, 104.86)	−14.02(−17.31, −10.29)	−0.39(−0.48, −0.31)
	Incidence	44,053.75 (35,811.01, 53,620.98)	3.91 (3.18, 4.76)	37,892.75 (33,122.34, 43,563.66)	3.31 (2.71, 3.95)	−15.37(−20.03, −10.47)	−0.44(−0.52, −0.36)
	Death	4,939.77 (3,899.98, 6,248.73)	0.50 (0.40, 0.62)	5,320.07 (3,751.94, 7,179.95)	0.30 (0.22, 0.39)	−40.54(−53.94, −25.86)	−1.90(−2.01, −1.79)
	DALYs	352,371.16 (288,189.85, 434,453.85)	31.45 (25.92, 38.46)	307,228.28 (242,179.13, 384,925.65)	17.98 (14.33, 22.11)	−42.81(−51.02, −34.23)	−2.05(−2.15, −1.96)
	YLDs	97,212.73 (64,866.26, 133,117.73)	9.51 (6.27, 12.79)	149,013.03 (98,580.06, 202,551.86)	7.97 (5.35, 10.70)	−16.26(−22.04, −9.93)	−0.50(−0.67, −0.34)
	YLLs	255,158.44 (195,369.38, 317,939.81)	21.93 (17.35, 27.28)	158,215.25(115,164.88, 208,411.93)	10.01 (7.37, 12.84)	−54.34(−64.19, −42.93)	−2.95(−3.12, −2.78)
Male							
Global	Prevalence	3,726,093.49 (3,484,081.01, 3,986,629.96)	150.90 (141.25, 160.98)	6,204,548.27 (5,774,781.93, 6,630,225.85)	151.85 (141.58, 162.39)	0.63(−1.12, 2.51)	0.04(0.02, 0.06)
	Incidence	156,993.79 (141,222.28, 173,481.47)	5.48 (4.98, 6.01)	236,634.13 (218,786.58, 254,835.80)	6.28 (5.77, 6.78)	14.61(10.36, 19.50)	0.45(0.41, 0.49)
	Death	46,028.44 (37,747.68, 54,972.33)	2.50 (2.05, 3.01)	104,975.08 (87,280.39, 123,437.70)	2.78 (2.30, 3.26)	11.20(−2.45, 21.96)	0.43(0.39, 0.47)
	DALYs	2,095,511.81 (1,783,982.41, 2,409,287.84)	90.23 (77.22, 104.71)	3,890,822.11 (3,322,946.41, 4,499,291.46)	97.57 (83.48, 112.80)	8.13(−0.81, 18.57)	0.29(0.27, 0.31)
	YLDs	290,366.00 (194,529.77, 388,108.30)	13.15 (8.77, 17.26)	575,620.09 (389,705.82, 758,506.70)	14.23 (9.66, 18.71)	8.25(3.72, 12.68)	0.35(0.25, 0.45)
	YLLs	1,805,145.80 (1,496,945.56, 2,115,239.78)	77.08 (64.01, 90.97)	3,315,202.02 (2,797,762.01, 3,932,576.43)	83.34 (70.41, 98.28)	8.11(−2.25, 20.07)	0.28(0.25, 0.30)
China	Prevalence	716,659.61 (657,864.26, 787,287.42)	121.78 (111.90, 133.19)	876,650.17 (793,247.69, 957,887.65)	102.94 (93.51, 112.64)	−15.47(−18.42, −12.43)	−0.48(−0.56, −0.40)
	Incidence	28,496.64 (23,555.87, 33,823.73)	4.84 (4.01, 5.75)	25,649.66 (22,570.18, 29,101.81)	4.26 (3.57, 4.99)	−11.85(−15.96, −7.57)	−0.31(−0.37, −0.24)
	Death	2,580.10 (1,873.64, 3,330.85)	0.54 (0.40, 0.71)	2,968.53 (1,950.54, 4,217.70)	0.36 (0.24, 0.50)	−33.23(−54.85, −9.25)	−1.42(−1.51, −1.33)
	DALYs	188,151.69 (145,680.49, 233,040.78)	33.15 (26.17, 40.64)	170,861.25 (132,378.91, 220,096.47)	20.48 (16.03, 25.89)	−38.20(−49.59, −24.75)	−1.76(−1.84, −1.68)
	YLDs	51,886.71 (34,670.62, 71,034.07)	10.26 (6.90, 13.67)	79,758.88 (52,770.06, 109,255.72)	8.81 (5.89, 11.82)	−14.07(−20.90, −6.38)	−0.43(−0.60, −0.26)
	YLLs	136,264.98 (94,298.96, 175,838.70)	22.89 (16.30, 29.31)	91,102.37 (62,566.37, 129,746.81)	11.67 (8.15, 16.28)	−49.02(−62.85, −29.78)	−2.52(−2.67, −2.38)
Female							
Global	Prevalence	2,644,788.80 (2,434,846.89, 2,883,624.05)	106.92 (98.49, 116.09)	4,531,260.82 (4,169,272.46, 4,956,553.10)	108.50 (99.78, 118.56)	1.47(−0.79, 3.58)	0.09(0.06, 0.11)
	Incidence	87,235.45 (77,100.18, 99,306.40)	3.10 (2.75, 3.50)	120,653.87 (109,595.62,133,046.22)	3.38 (3.04, 3.77)	9.05(3.56, 14.75)	0.28(0.24, 0.32)
	Death	37,141.31 (31,113.79, 43,461.83)	1.67 (1.38, 1.96)	89,022.32 (73,524.03, 105,883.36)	1.99 (1.66, 2.36)	19.19(6.07, 29.67)	0.60(0.54, 0.66)
	DALYs	1,655,576.61 (1,429,752.07, 1,885,529.42)	67.19 (57.78, 76.73)	3,068,935.54 (2,640,502.08, 3,521,005.63)	72.47 (62.80, 82.98)	7.86(−6.02, 17.28)	0.23(0.19, 0.28)
	YLDs	229,101.49 (152,352.26, 302,800.14)	9.77 (6.47, 12.90)	446,186.85 (299,118.79, 591,612.39)	10.37 (6.95, 13.84)	6.13(1.91, 10.41)	0.33(0.24, 0.41)
	YLLs	1,426,475.12 (1,208,253.42, 1,646,129.07)	57.43 (48.77, 66.11)	2,622,748.69 (2,233,804.83, 3,101,473.28)	62.10 (53.19, 72.88)	8.15(−7.83, 18.90)	0.22(0.16, 0.27)
China	Prevalence	536,772.89 (467,547.38, 608,802.37)	97.35 (84.98, 110.60)	733,638.30 (628,063.00, 851,344.07)	85.37 (72.91, 98.94)	−12.30(−16.53, −7.53)	−0.28(−0.39, −0.18)
	Incidence	15,557.11 (12,271.80, 19,714.16)	2.91 (2.29, 3.67)	12,243.10 (10,261.45, 14,483.79)	2.27 (1.79, 2.80)	−22.10(−27.93, −15.62)	−0.70(−0.82, −0.58)
	Death	2,359.67 (1,820.25, 3,115.00)	0.48 (0.37, 0.62)	2,351.55 (1,533.50, 3,292.22)	0.25 (0.17, 0.35)	−47.81(−62.72, −30.87)	−2.42(−2.56, −2.27)
	DALYs	164,219.48 (133,751.34, 203,895.74)	30.05 (24.77, 37.26)	136,367.03 (104,392.62, 170,179.45)	15.70 (12.16, 19.37)	−47.73(−57.12, −37.39)	−2.38(−2.49, −2.27)
	YLDs	45,326.02 (30,151.66, 62,799.98)	8.94 (5.82, 12.05)	69,254.15 (45,973.35, 93,208.82)	7.28 (4.85, 9.80)	−18.50(−24.35, −11.82)	−0.58(−0.75, −0.40)
	YLLs	118,893.46 (89,335.02, 151,300.77)	21.11 (16.42, 26.65)	67,112.88 (45,389.72, 93,621.33)	8.42 (5.91, 11.61)	−60.11(−71.20, −46.42)	−3.45(−3.65, −3.26)

CKD: chronic kidney disease; ASR: age-standardized rate; DALYs: disability-adjusted life years; YLDs: years lived with disability, YLLs: years of life lost; EAPC: estimated annual percent change

The total number of DALYs for glomerulonephritis-induced CKD in 2021 was 307,228 person-years, with age-standardized DALYs rate of 20.48/100,000 for males (95% UI: 16.03, 25.89), 15.70/100,000 for females (95% UI: 12.16, 19.37), and 17.98/100,000 overall (95% UI: 14.33, 22.11) (Table 1). Males experienced the highest number of DALYs in the 50 to 54 and 55- to 59-year age groups, with the age-standardized DALYs rate increasing with age, peaking in the 90- to 94-year group (Supplementary Figure 1B). YLDs totaled 149,013 person-years, with a higher contribution from males (79,759 person-years). The age-standardized YLDs rate was 8.81/100,000 for males (95% UI: 5.89, 11.82) and 7.28/100,000 for females (95% UI: 4.85, 9.80) (Supplementary Figure 2A, Table 1). YLLs accounted for 158,215 person-years and were higher in males than in females (Supplementary Figure 2B, Table 1).

**Figure 2.**
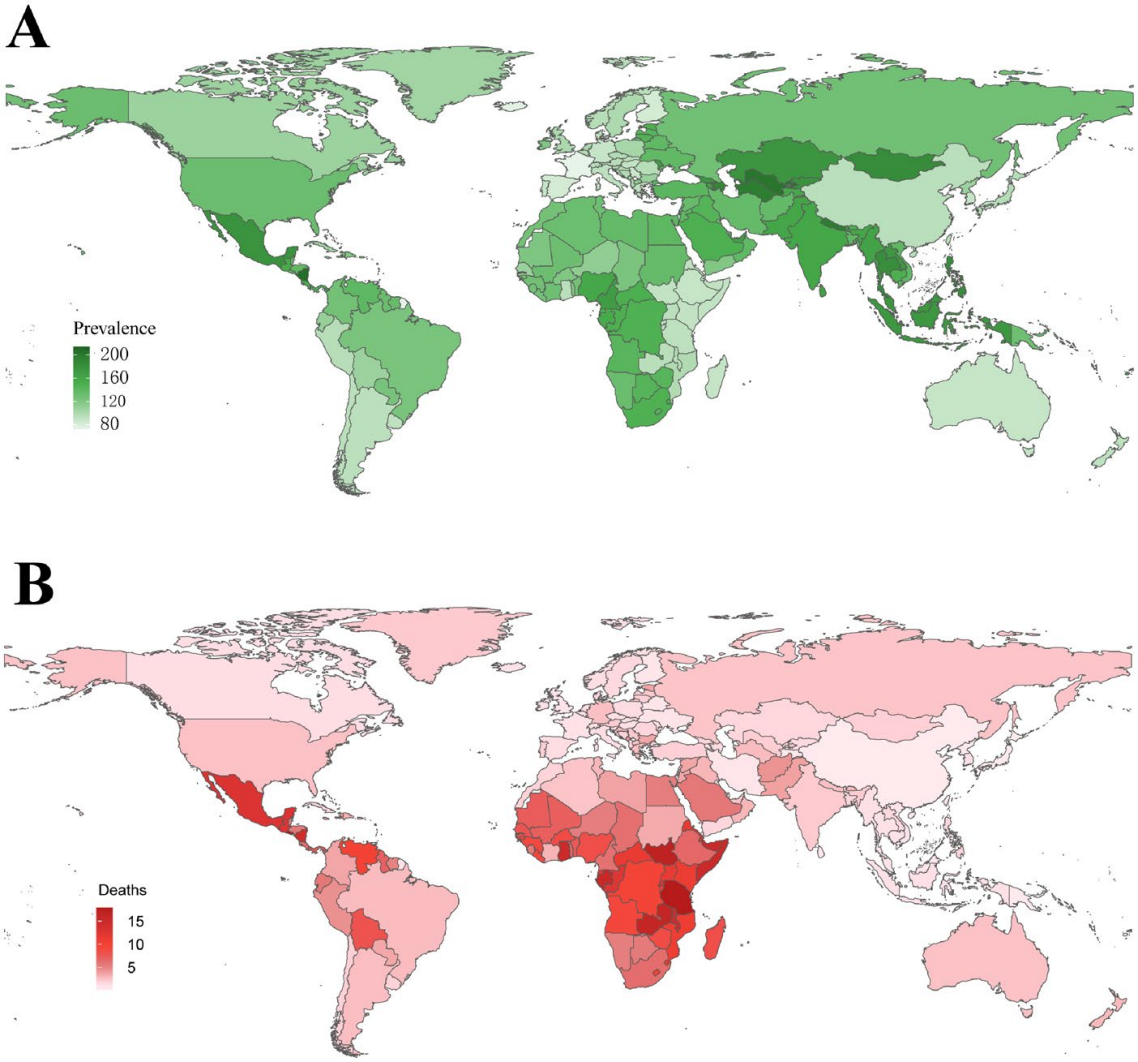
Global glomerulonephritis-induced CKD burden in 204 countries and regions in 2021. (A) ASPR in the general population. (B) ASDR in the total population. ASPR: age-standardized prevalence rate; ASDR: age-standardized death rate.

Figure 2 and Supplementary Figure 3 present the ASPR, ASIR, ASDR, and age-standardized DALY rate resulting from glomerulonephritis-induced CKD in 2021 for 204 countries and regions. These data were ranked for each country and region and categorized as quartiles. Globally, China’s total population exhibited relatively low levels of ASPR, ASIR, ASDR and age-standardized DALYs rate.

**Figure 3.**
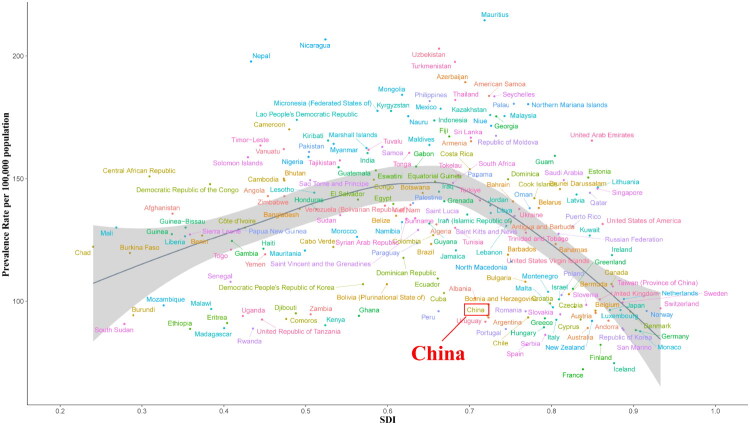
ASPR attributable to glomerulonephritis-induced CKD across 204 countries and regions by sociodemographic index, 1990–2021. The black line indicates an adaptive association fitted with adaptive Loess regression based on all data points.

Figure 3 and Supplementary Figures 4, 5, 6 illustrate the correlation between the SDI and the ASPR, ASIR, ASDR, and age-standardized DALYs rate. The ASPR and ASIR initially increased and subsequently declined with an increase in SDI. In contrast, both the ASDR and age-standardized DALY rates exhibited a declining trend with increasing SDI. Notably, China’s rates were lower than those anticipated across all these metrics.

### Age-specific disease burden trends in China (1990–2021)

**Figure 4.**
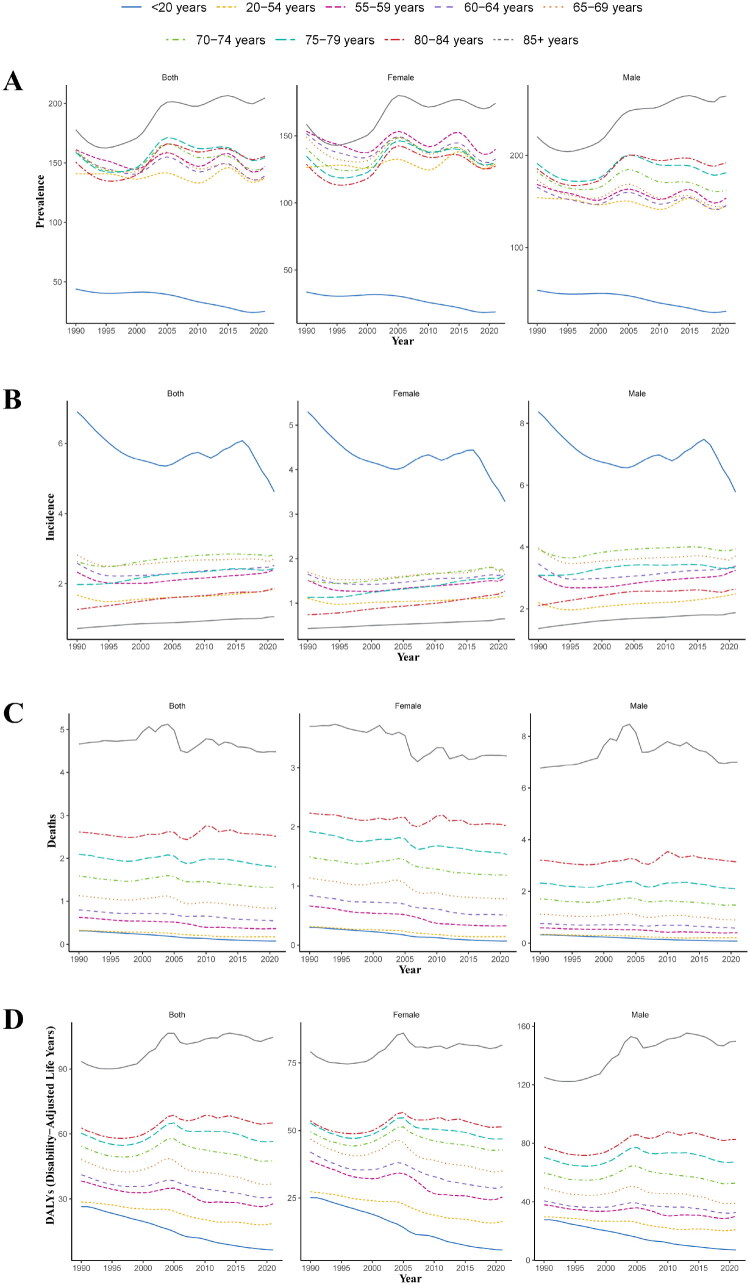
Trends in age-stratified ASPR, ASIR, ASDR, and age-standardized DALYs rate for glomerulonephritis-induced CKD in males and females in China from 1990 to 2021. (A) ASPR. (B) ASIR. (C) ASDR. (D) Age-standardized DALYs rate. ASPR: age-standardized prevalence rate; ASIR: age-standardized incidence rate; ASDR: age-standardized death rate; DALYs: disability-adjusted life years.

Analysis of 1990–2021 trends revealed significant shifts in the glomerulonephritis-induced CKD burden, with marked sexual dimorphism. Specifically, the ASPR showed a consistent decline in individuals younger than 20 years. Conversely, other age groups exhibited more erratic temporal patterns, culminating in peak ASPR values among those aged ≥85 years. This burden uniformly demonstrated male predominance throughout all age strata, a pattern corroborated by Figure 4(A). The ASIR among younger populations declined sharply from 1990 to 2005, subsequently rose gradually until 2016, then exhibited a steep decline. Conversely, all other age groups demonstrated steady incidence growth throughout the study period, with male subjects consistently maintaining disproportionately elevated rates relative to females during the entire observation timeframe (Figure 4(B)). ASDR declined for females, reflecting improved survival, whereas male mortality increased after the age of 85 years, peaking in 2005 (Figure 4(C)). Age-standardized DALYs rate followed a similar trend, with males aged 85 years and older showing the heaviest burden (Figure 4(D)).

### Comparison between China and global disease burden trends (1990–2021)

Between 1990 and 2021, China observed a decline in ASPR, from 109.57 to 94.21 per 100,000, whereas globally, ASPR increased from 128.55 to 129.94 per 100,000. In addition, China experienced a slight decrease in ASIR from 3.91 to 3.31 per 100,000, contrasting with a global increase. ASDR in China dropped from 0.50 to 0.30 per 100,000, in contrast to the global upward trend. Age-standardized DALYs rate in China decreased from 31.45 to 17.98 per 100,000, as opposed to the global increase (Table 1 and Supplementary Figure 7). These trends indicated a significant improvement in CKD burden in China compared to a worsening global impact.

The annual percentage change (EAPC) from 1990 to 2021 revealed that China’s ASPR, ASIR, ASDR, and age-standardized DALYs rate decreased significantly, with rates of −0.39, −0.44, −1.90, and −2.05, respectively (Table 1, Supplementary Table 2). In contrast, global rates increased in all categories, particularly for ASIR and ASDR.

### APC analysis of CKD due to glomerulonephritis in China

The APC analysis of CKD incidence and mortality due to glomerulonephritis in China reveals distinct trends. The incidence demonstrated a net drift of 0.51% (*p* < 0.05), indicating a gradual increase (Supplementary Figure 8A). It peaked in the 1–4 year age group, declined during childhood, increased again around the age of 45 years, and peaked between 70 and 75 years before decreasing (Supplementary Figure 8B, Supplementary Table 3). A slight upward trend in the incidence was observed over time (Supplementary Figure 8C). Cohort analysis revealed that the relative risk (RR) for developing CKD increased from 0.67 (95% CI: 0.43–1.04) in the 1902–1907 cohort to 1.17 (95% CI: 1.12–1.22) in the 1997–2002 cohort, and subsequently declined to 0.81 (95% CI: 0.78–0.85) in the 2017 cohort (Supplementary Figure 8D and Supplementary Table 3). However, age-specific incidence analysis demonstrated a decline in younger groups (<5 and 5–9 years) along with an increase in middle-aged and elderly groups (45–75 years) (Supplementary Figure 8E). Later birth cohorts exhibited a steady decline in incidence within each age group (Supplementary Figure 8F).

**Figure 5.**
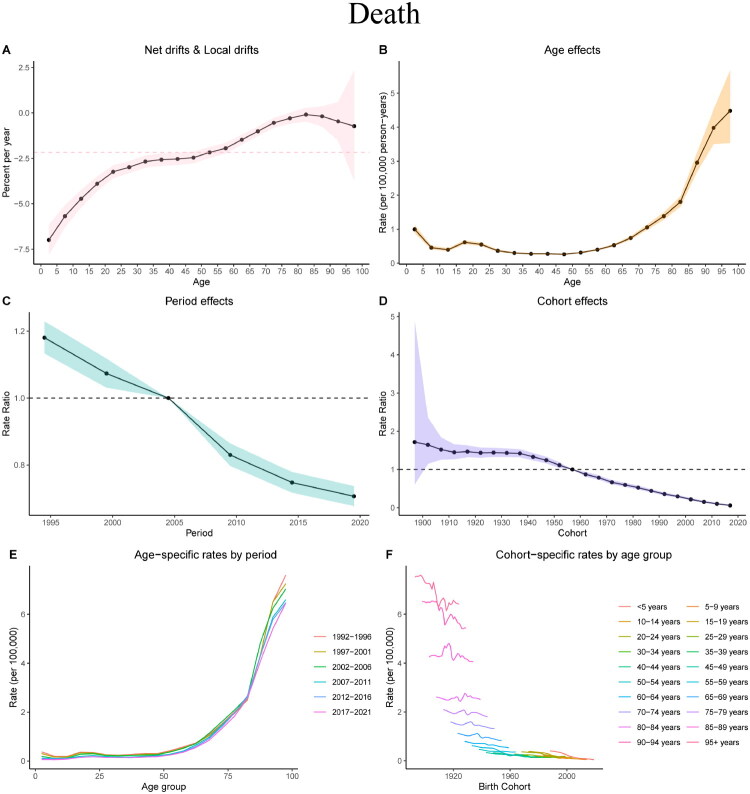
Age, period, and cohort effects on death from glomerulonephritis-induced CKD in China from 1990 to 2021. (A) Local drift and net drift values. (B) Longitudinal age curves. (C) Period effects. (D) Cohort effects. (E) Age-specific death rates according to period. Each line connects the age-specific death rate for the 5-year period group. (F) The cohort-specific death rate according to age groups. Each line connects the cohort-specific death rates for the 5-year age group.

Mortality analysis revealed a significant decrease, indicating reduced mortality rates, with a total drift of −2.17% (*p* < 0.05) (Figure 5(A)). Mortality was low and stable for individuals under 45 years of age, increasing progressively with age and peaking in the 95+ age group (Figure 5(B)). The rates decreased from 1995 to 2020 (Figure 5(C)). Previous birth cohorts (e.g. 1910) had higher mortality risks, which declined in subsequent cohorts (Figure 5(D)). Age-specific mortality analysis revealed decreases across all age groups with each passing cohort (Figure 5(E)). The cohort-specific analysis demonstrated reduced mortality rates within the same age groups in later cohorts (Figure 5(F)). Wald tests confirmed significant differences among age, period, and cohort effects (*p* < 0.05), highlighting these factors as major determinants of glomerulonephritis-induced CKD incidence and mortality (Supplementary Tables 3 and 4).

### Decomposition analysis of factors affecting the incidence and mortality of glomerulonephritis-induced CKD in China

**Figure 6.**
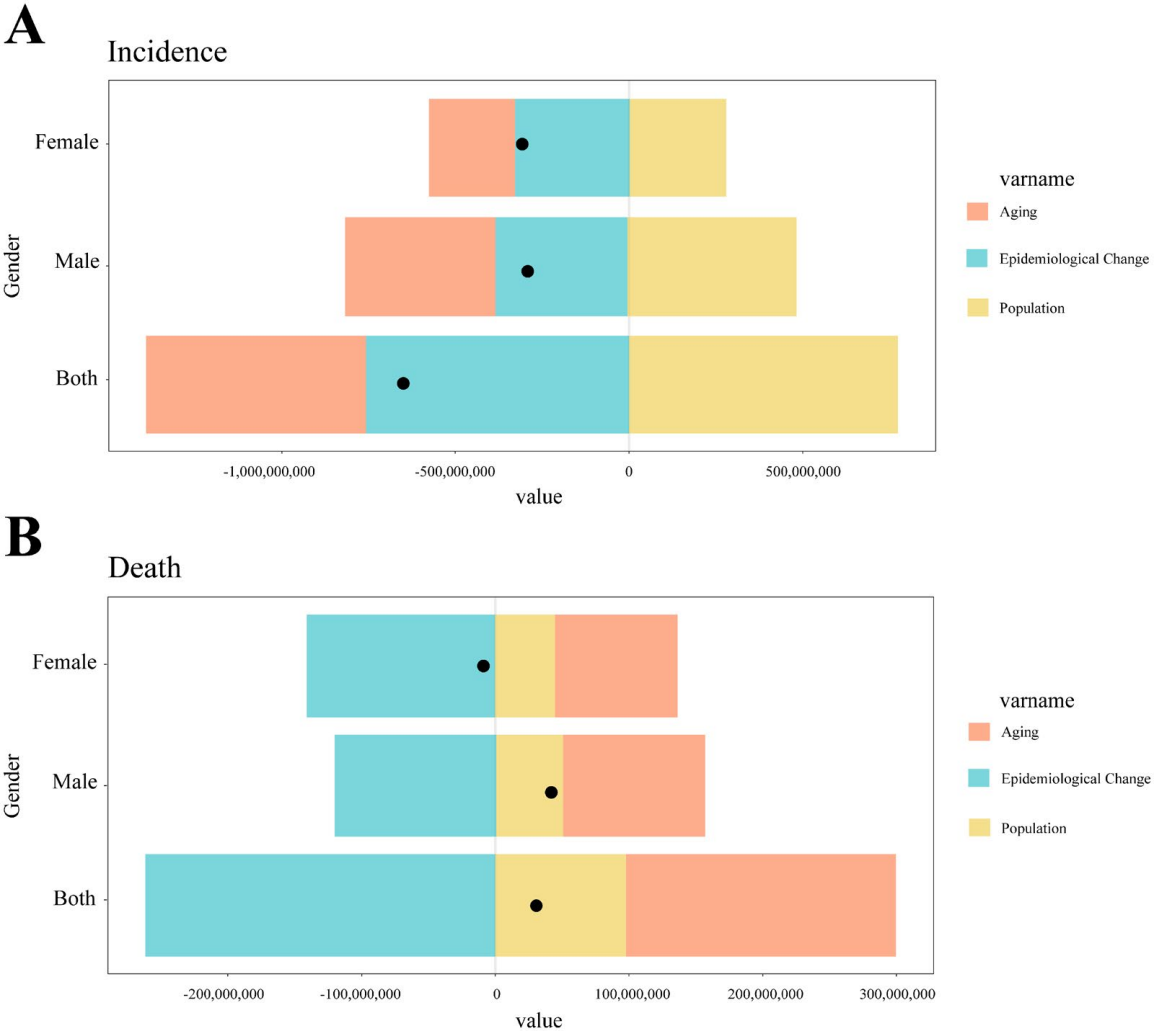
A decomposition analysis of contributions of aging, epidemiological changes, and population factors to the incidence and deaths of glomerulonephritis-induced CKD in China, stratified by sex. Positive values on the x-axis indicate an increase in incidence or deaths, whereas negative values indicate a decrease. The black dots represent the total impact of these three factors. (A) Contributions of aging, epidemiological changes, and population factors to the incidence for both sexes, males, and females. (B) Contributions of aging, epidemiological changes, and population factors to the deaths for both sexes, males, and females.

Our analysis revealed the complex interplay of population aging, growth, and epidemiological shifts on the incidence and mortality of glomerulonephritis-induced CKD in China. Aging and epidemiological changes primarily caused the decline in the incidence, whereas population growth mitigated this effect (Figure 6(A)). Mortality was influenced by aging and growth, with epidemiological changes causing a more significant reduction in female mortality (Figure 6(B)).

Between 1990 and 2021, aging significantly reduced the CKD incidence in China (total: 102.6%, male: 125.29%, female: 78.37%) and an increase in mortality (total: 530.30%, male: 275.32%). Population growth mitigated these effects, reducing the decline in the incidence (total: −125.58%, male: −172.17%) and increasing mortality rates (total: 257.74%, male: 129.55%). Epidemiological trends, notably a decrease in female mortality, offset the increase attributed to aging and growth (Table 2 and Figure 6(B)). These results highlight the complex interplay of aging, population growth, and epidemiological trends that influence the CKD burden in China.

**Table 2.** Impact of aging, population growth, and epidemiological trends on changes in glomerulonephritis-induced CKD incidence and death rates in China in 1990 and 2021.

Location	Measure	Sex	Overall difference	Change due to population-level determinants (% contribution to total changes)		
				Aging	Population	Epidemiological change
China	Incidence	Both	−616,099,546.16	−632,108,032.72 (102.60%)	773,715,923.53 (−125.58%)	−757,707,436.97 (122.98%)
		Male	−284,698,245.70	−356,699,168.59 (125.29%)	490,159,743.57 (−172.17%)	−418,158,820.67 (146.88%)
		Female	−331,401,300.46	−259,732,267.80 (78.37%)	273,487,258.53(−82.52%)	−345,156,291.19 (104.15%)
	Death	Both	38,030,561.59	201,676,394.63 (530.30%)	98,020,518.61 (257.74%)	−261,666,351.65 (−688.04%)
		Male	38,842,608.24	106,941,684.64 (275.32%)	50,320,615.20 (129.55%)	−118,419,691.60 (−304.87%)
		Female	−812,046.65	97,069,980.03 (−11,953.74%)	47,598,829.38 (−5,861.59%)	−145,480,856.06 (17,915.33%)

### Projections of disease burden of glomerulonephritis-induced CKD in China (2021–2050)

**Figure 7.**
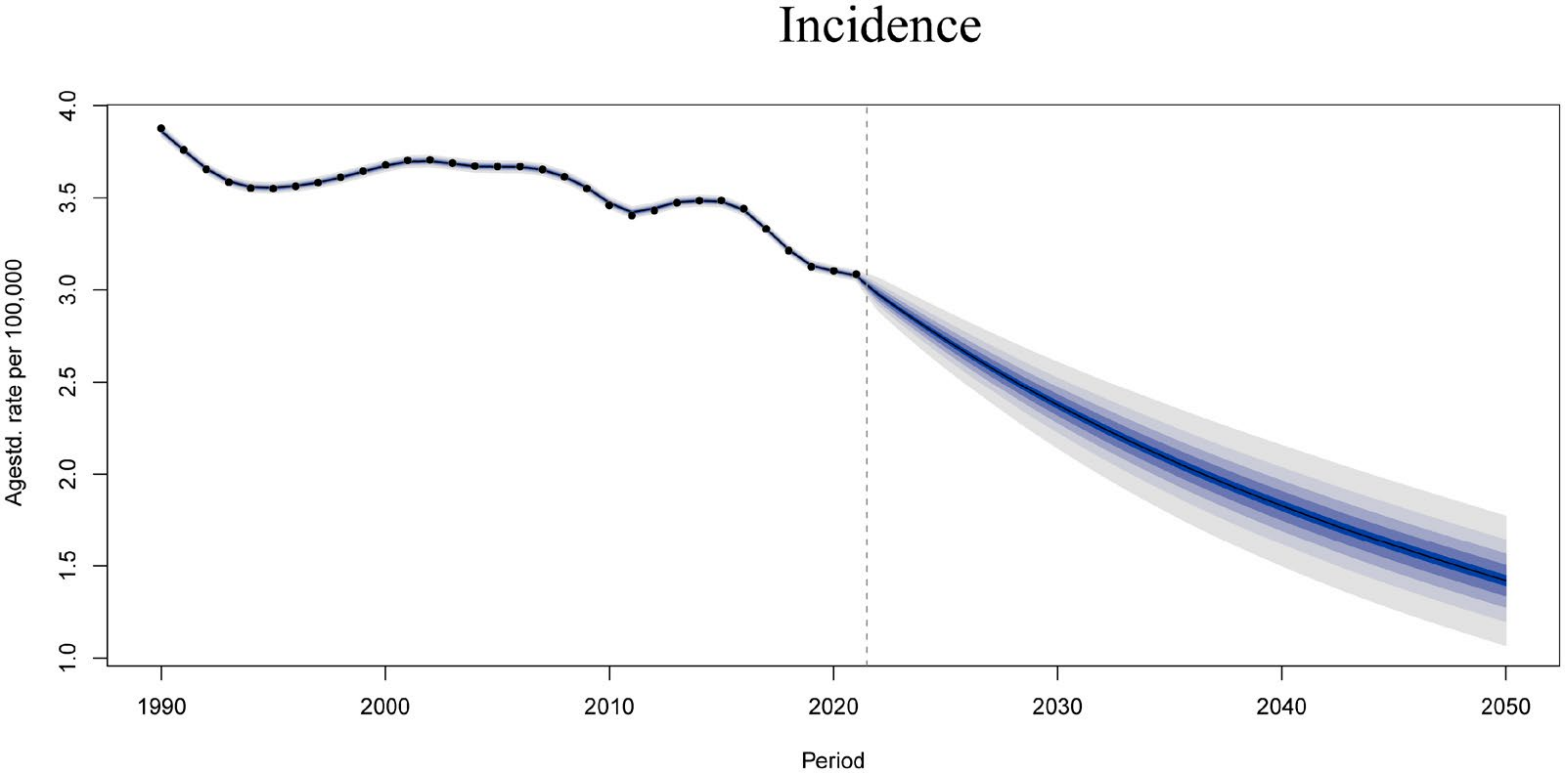
BAPC analysis predicting the ASIR of glomerulonephritis-induced CKD in China by 2050: predicted ASIR for the total population. BAPC: Bayesian age-period-cohort.

From 2021 to 2050, the BAPC model predicts declines in ASPR and ASIR for glomerulonephritis-induced CKD in China, with ASPR reducing from 94.43 to 58.99 per 100,000 and ASIR, respectively, from 3.08 to 1.42 per 100,000 (Supplementary Figures 9 and Figure 7, Supplementary Table 5). Despite an overall decrease, the incidence among those aged 45 years and older is expected to increase (Supplementary Figure 10). Similarly, ASDR and age-standardized DALY rates are estimated to decrease (Supplementary Figures 11 and 12, Supplementary Table 5). These findings underscore the imperative for targeted screening among China’s aging population.

### Disease burden attributable to risk factors for glomerulonephritis-induced CKD in China and globally

**Figure 8.**
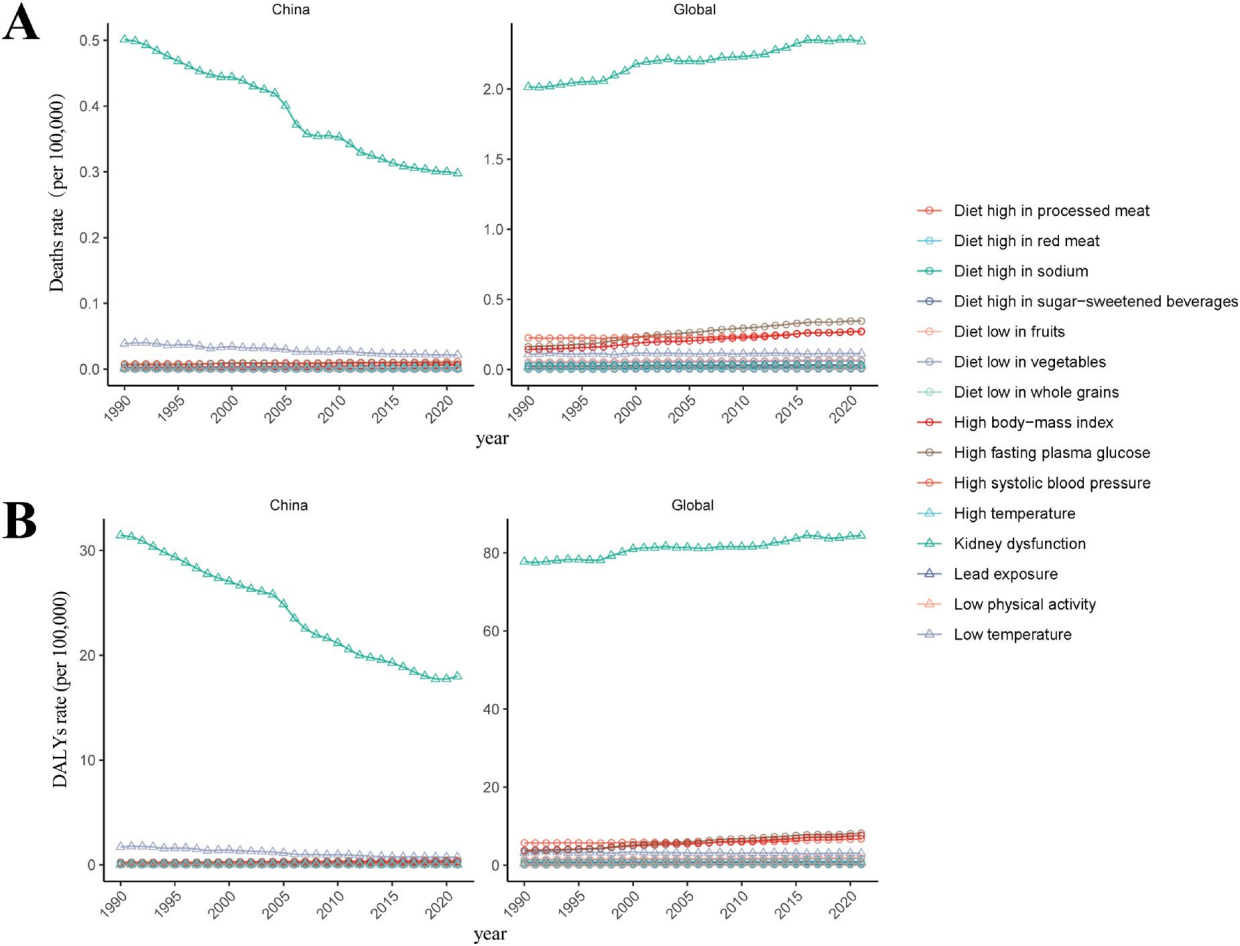
Trends in ASDR and age-standardized DALYs rate attributable to different risk factors for glomerulonephritis-induced CKD in China and globally from 1990 to 2021. (A) ASDR attributable to different risk factors. (B) Age-standardized DALYs rate attributable to different risk factors.

We evaluated the impact of 15 risk factors on glomerulonephritis-induced CKD in China and globally. In 2021, the primary contributors to CKD burden were kidney dysfunction, high fasting plasma glucose, high BMI, and high systolic blood pressure in both China and globally (Supplementary Figure 13 and Supplementary Table 6). From 1990 to 2021, a decline in CKD ASDR and age-standardized DALYs rate associated with these factors was observed in China, contrasting with a global increase. Other risk factors exerted a minimal impact on disease burden and remained largely stable over time (Figure 8 and Supplementary Table 6).

### Global health inequality in glomerulonephritis-induced CKD

**Figure 9.**
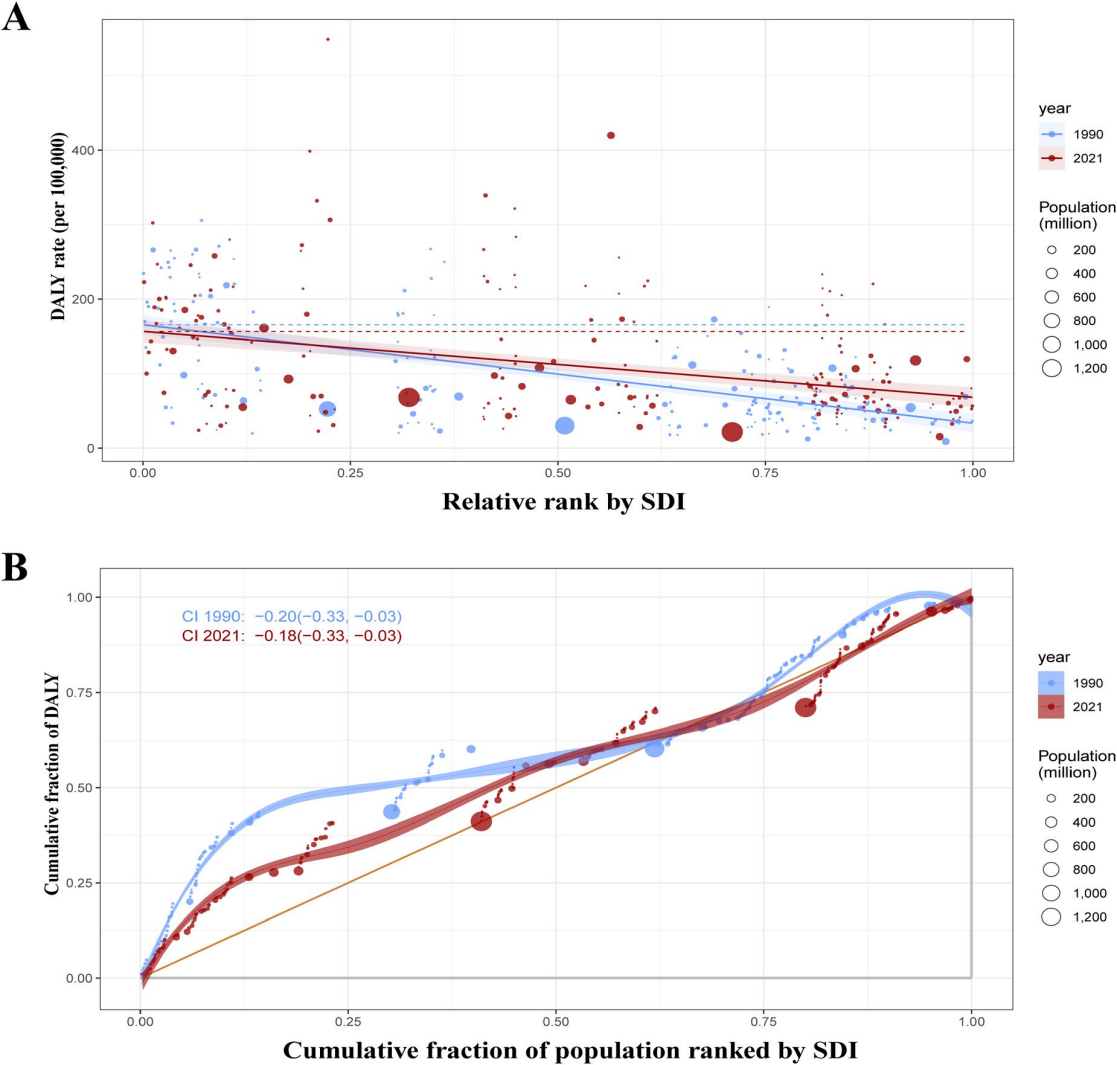
Health inequality regression curves and concentration curves for DALY due to glomerulonephritis-induced CKD worldwide. DALY: disability adjusted life year. SDI: socio-demographic index.

From 1990 to 2021, global health disparities in glomerulonephritis-induced CKD, as quantified by DALYs, displayed a marginal decrease. The disparity between nations with high and low SDI scores narrowed, with the gap reducing from −132.21 to −88.40 (Figure 9 and Table 3). Despite this modest improvement, significant inequalities remain, particularly for lower SDI countries, which continue to encounter a disproportionately higher CKD burden.

**Table 3.** Inequality slope Index and concentration Index for disability-adjusted life years of glomerulonephritis-induced CKD in 1990 and 2021.

	Slope index of inequality	Concentration index
1990	−132.21 (−156.17, −108.24)	−0.20 (−0.33, −0.03)
2021	−88.40 (−116.36, −60.44)	−0.18 (−0.33, −0.03)

## Discussion

In the present study, we analyzed the disease burden and risk factors associated with glomerulonephritis-induced CKD in China and globally from 1990 to 2021. In addition, we have projected its development over the next 29 years. The results indicate that, between 1990 and 2021, the disease burden of glomerulonephritis-induced CKD in China was significantly lower than the global average. China’s ASPR, ASIR, ASDR and ASR of DALYs, YLDs, YLLs displayed a declining trend, which is strikingly different from the global increase in glomerulonephritis-induced CKD burden. Period effect analysis revealed that mortality rates in the overall population have gradually decreased over time, possibly due to advances in medical technology and the introduction of novel immunosuppressive therapies. These advancements have led to delayed disease progression and prolonged survival [34]. Similarly, enhancements in public health infrastructure have immensely contributed to alleviating the CKD burden by strengthening environmental health standards, ensuring safe drinking water, and reducing pollutants in the living environment. They have contributed to mitigating kidney damage caused by heavy metals and chemicals, thus lowering the incidence and mortality rates of glomerulonephritis-induced CKD [35]. Furthermore, decomposition analysis identified epidemiological transitions as the predominant driver of China’s declining incidence and mortality. Intriguingly, population aging was associated with reduced CKD incidence in older age groups, reflecting China’s unique demographic-epidemiological transition: despite an expanding elderly population from 1990 to 2021, nationwide strengthening of prevention systems, lifestyle modifications, and improved renal care accessibility [36] drove downward age-specific incidence trends. Conversely, aging exacerbated mortality burdens, as elderly CKD patients exhibited higher case-fatality rates due to elevated comorbidity prevalence and progression risks, aligning with global patterns [37]. While decomposition analysis quantified static factor contributions, it inadequately captured dynamic interactions – for instance, the synergistic interactions between social determinants and environmental changes. Future investigations should integrate time-series decomposition methods to track the synergistic evolution of demographic shifts, healthcare interventions, and socioeconomic determinants. Such approaches will elucidate temporally varying contributions of multifaceted factors, providing critical evidence for precision prevention strategies.

Glomerulonephritis-induced CKD burden was found to be significantly higher in the male population in China than in females, underscoring the ongoing sex disparity in the disease burden. This phenomenon could be partly attributed to sex differences in the progression of kidney disease. Research suggests that testosterone in males may adversely affect renal function and structure, whereas estrogen in females plays a protective role in kidney health [38]. In addition, differences in specific autosomal gene expression [39] and glomerular hemodynamics [40] between males and females may contribute to variations in the extent of GFR decline. Moreover, unhealthy behaviors, such as smoking and alcohol consumption, are more commonly observed in males, which may accelerate glomerulonephritis-induced CKD progression and increase mortality risk [41,42]. In contrast, females tend to adopt healthier lifestyles and exhibit more proactive health-seeking behaviors [43]. For example, compared to females, males with lupus nephritis are less likely to make outpatient appointments or visit the emergency department [44]. Swartling et al. reported that glomerulonephritis-induced CKD progression is slower in females than in males, including the risk of complications, such as cardiovascular diseases (CVD). Thus, the all-cause mortality and cardiovascular mortality rates of glomerulonephritis-induced CKD are lower in females [45,46]. Other studies have highlighted that sex-related differences in the control of adverse risk factors (e.g. higher BMI, blood phosphorus, low-density lipoprotein (LDL) cholesterol, and inflammatory markers) and nitric oxide metabolism may influence the final outcomes of glomerulonephritis-induced CKD in males and females [47]. Therefore, sex-specific risk factors should be incorporated into disease screening and prevention strategies to reduce disease burden and improve overall public health.

We further analyzed glomerulonephritis-induced CKD burden in different age groups in China and identified distinct age-related distribution patterns. The highest incidence was observed in those below 20 years of age, particularly in the 1–4 year age group, which aligned with global trends [9]. Comparable findings have also been documented in other studies, including that by Hiep et al. in Belgium, which identified the 0–4 year age range as the peak period for CKD detection [48]. This could be attributed to different factors, including renal developmental abnormalities or dysfunction during infancy, neonatal acute kidney injury, and complications arising from prematurity and low birth weight [49]. In addition, an immature immune system in children increases their susceptibility to infections by pathogens such as influenza viruses, Epstein–Barr virus, and streptococci, with post-streptococcal glomerulonephritis being particularly prevalent in this age group [50]. Inadequate treatment of these infections can cause CKD progression. Therefore, early screening, diagnosis, and intervention for CKD in children aged 1–4 years should be prioritized to prevent ESKD progression. Notably, a transient decline in the prevalence of glomerulonephritis-induced CKD was observed between the 55–59 and 60–64 age groups in China. This phenomenon may be closely linked to recent standardization of diagnostic protocols: for instance, the adoption of the unified CKD-EPI equation for eGFR calculation has not only improved data comparability but also standardized threshold settings, directly influencing the statistical outcomes in elderly populations [21]. Additionally, the implementation of comprehensive CKD prevention and management strategies, enhanced public health awareness, and improved self-management capabilities among patients may have collectively contributed to this trend [51,52]. Glomerulonephritis-induced CKD burden in China, as measured by mortality and DALYs, increases with age, with a particularly pronounced increase in mortality and DALYs in the 60+ age group. This trend is intricately linked to the aging population in China, with the proportion of elderly individuals steadily increasing [53], as well as a natural decline in organ function and the development of multi-organ comorbidities in older adults [54]. Moreover, decomposition analysis established population aging as the principal driver underpinning rising glomerulonephritis-induced CKD incidence and mortality in China. The National Bureau of Statistics data state that the proportion of elderly individuals in China was 18.9% in 2021, significantly higher than the 5.63% recorded in 1990(55). China’s population of individuals aged 65 years and older is estimated to increase to 400 million by 2050, with those aged 80 years and older accounting for 150 million, thus making China one of the countries with the highest proportion of elderly people worldwide [55]. This demographic shift will pose a significant challenge to CKD prevention and management in China. Thus, effective strategies for CKD prevention and management should consider differences across age groups. Special attention should be paid to the elderly population, with a focus on health education and the development of tailored follow-up plans. In particular, elderly men should be prioritized in CKD prevention efforts to delay disease progression and reduce mortality risk.

The APC effect displayed an overall increasing trend in incidence rates in China, which contrasted with the trend observed in the ASIR from 1990 to 2021. The relatively wide confidence interval for the net drift in the APC effect (95% UI: 0.15, 0.86) suggested a degree of uncertainty regarding this upward trend. This could be driven by factors such as demographic shifts (for instance, aging population) [56], unhealthy dietary patterns [57,58], and worsening environmental pollution [59,60], which could contribute to an increased incidence, causing fluctuations in the net drift value. Further analysis of age-specific incidence rates by period revealed an upward trend in the middle-aged and elderly groups (45–75 years), possibly influenced by factors such as aging, accumulation of chronic diseases, and renal function decline [61,62]. Conversely, the cohort-specific incidence rates showed a decreasing trend within the same age groups over time, suggesting the positive impact of social development, including improvements in medical testing, increased public health awareness, and the integration of CKD into national public health monitoring programs [63]. These collectively reduced the disease risk for subsequent birth cohorts at the same age. Effective prevention and management strategies that meet the unique needs of different populations are required for glomerulonephritis-induced CKD due to the complex interplay of multiple factors influencing disease burden highlighted by the age-period-cohort effects. For example, chronic disease management, regular health monitoring, and guidance on kidney-protective lifestyle choices should be prioritized for the elderly. Early screening, vaccination, and education on healthy lifestyle choices should be emphasized for younger populations. Personalized interventions may assist in effectively mitigate the disease burden, reduce incidence, and improve prognosis.

The BAPC model predicts that over the next 29 years, the glomerulonephritis-induced CKD burden in China will continue to decline across key indicators, including overall prevalence, incidence, mortality, and DALYs. This downward trend is attributed to improvements in healthcare, implementation of early diagnosis and treatment strategies, and public health initiatives aimed at mitigating risk factors. However, further projections for disease burden in different age groups displayed an upward trend in the incidence among those aged 45 years and above. This trend may be partially attributed to improved detection rates driven by the popularization of renal biopsy technology and the improvement of screening systems, rather than an actual increase in the true incidence of the disease; future studies should incorporate renal biopsy data to conduct in-depth verification of the true incidence in this age group. Nevertheless, although China’s rapid economic growth, continued improvement in healthcare infrastructure, and increasing public awareness of health have generally reduced the burden of glomerulonephritis-induced CKD, the absolute number of cases in middle-aged and elderly populations is expected to continue to increase. This increase can largely be attributed to the combined effects of population growth, aging demographics, screening-driven case ascertainment, and improved survival rates [64–67]. Consequently, sustained and adaptive healthcare plans addressing the evolving needs of these age groups are warranted. In particular, targeted public health intervention strategies for middle-aged and elderly populations are essential to mitigate the growing disease burden and improve health outcomes. While the BAPC model accounts for demographic changes, two healthcare limitations exist: 1) Diagnostic policies: The CKD Screening Guidelines (2022) [68] require universal adult screening with annual urine albumin-to-creatinine ratio (UACR) and serum creatinine testing, but GBD data lack granularity to quantify these protocol-driven changes. 2) Treatment access: Regional disparities persist in nephrology care [69]. Our model’s assumption of uniform CKD management may underestimate rural burdens.

Our analysis of 15 risk factors for glomerulonephritis-induced CKD revealed that metabolic factors – specifically, impaired kidney function, high fasting plasma glucose, high BMI, and high systolic blood pressure – primarily contribute to CKD burden in China. The following strategies can be implemented to effectively reduce the burden of CKD: First, high-risk individuals with these risk factors should receive targeted and efficient management strategies to reduce their exposure to these factors and prevent CKD progression. Second, multidisciplinary collaboration [70], with a strong emphasis on primary healthcare, should be implemented. This includes utilizing information technology to detect and prevent the disease, formulating policies for early screening, optimizing monitoring processes, and conducting regular tests for kidney function and urine routine analysis. Furthermore, public education on CKD should be strengthened, focusing on its severe consequences, and awareness should be raised about prevention among the general population. In addition, it is essential to promote healthy lifestyle choices and dietary habits such as smoking cessation [71], regular physical activity [72], maintaining a healthy weight [73], and following a diet low in salt [74], low in fat, and controlled in sugar. For the general population, personalized health plans should be developed to gradually cultivate good habits, whereas for patients with CKD along with hypertension or diabetes, comprehensive treatment strategies, tailored to individual patient needs, including precise medication regimens and combined dietary and exercise interventions, should be employed to maintain stable blood pressure and blood glucose levels, thereby delaying CKD progression. While population growth and aging drive mortality trends, our GBD analysis has risk factor gaps, omitting biologically plausible determinants of glomerulonephritis progression including recurrent infections (such as streptococcal, hepatitis B/C), malnutrition, and autoimmune diseases [75–79] – factors likely to disproportionately impact aging populations and regions with rapid demographic transitions. These omissions likely reflect challenges in standardizing global exposure estimates and establishing dose-response relationships across diverse populations. Furthermore, socioeconomic determinants like healthcare access inequities and environmental hygiene, though partially captured through the SDI, may interact with biological aging processes in complex ways not fully accounted for in our models. Future research should prioritize integrating region-specific epidemiological data through national health surveys and cohort studies to better characterize these under-investigated risk profiles, particularly in low-resource settings where infection-related glomerulonephritis remains prevalent.

We observed significant negative values for both SII and CI in patients with CKD, which are attributable to glomerulonephritis globally. These negative values demonstrate that countries with lower SDI have higher DALY rates and DALY number, signifying that glomerulonephritis-induced CKD is disproportionately concentrated in low-SDI countries. This finding is consistent with those of previous reports [9,15]. Potential factors contributing to this phenomenon include limited healthcare services in these countries, aging populations, and rapid population growth [80,81]. Furthermore, we found that infection-related glomerulonephritis is a major contributor to CKD in several economically disadvantaged regions [82], likely linked to poor healthcare infrastructure and suboptimal disease control in countries with lower socioeconomic status. In contrast, highly developed SDI countries can ensure the widespread availability of high-quality healthcare services, significantly improving health outcomes and reducing premature mortality risks [83,84]. This ongoing global imbalance underscores the critical need for focused public health interventions to gradually reduce the unequal disease burden caused by SDI disparities and promote global health equity.

This study had several limitations. First, the GBD database-derived estimates were based on statistical models constructed using real-world data, rather than direct observational data [85]. Thus, the reliability of our findings may be affected by data reporting systems and collection methods in certain countries, consequently limiting the accuracy and completeness of these estimates. As a public database managed by IHME, the GBD study integrates heterogeneous data sources through multi-level modeling; nevertheless, it may still underestimate the true disease burden in low-income settings – particularly where healthcare resources are scarce. This limitation may affect the applicability and result robustness of the disease burden calculation model based on GBD data in this study. Therefore, future work should prioritize strengthening primary data collection and quality-control protocols in these regions to improve the accuracy of global burden-of-disease estimates. It is particularly noteworthy that the risk attribution models within the CRA framework were primarily developed for glomerulonephritis-induced CKD as a composite endpoint, potentially overlooking etiology-specific risk profiles. For idiopathic glomerulonephritis, certain immune-mediated pathways may involve distinct genetic-environmental interactions that current metabolic and lifestyle risk factors fail to fully capture. Furthermore, ecological bias remains a concern when extrapolating population-level risk estimates to individual clinical outcomes, as unmeasured confounders such as genetic susceptibility and epigenetic modifications could modify the effects of risk factors. Second, the operational definition of glomerulonephritis-induced CKD (ICD-10: N02–N06.9) encompasses diagnostic categories including recurrent hematuria, chronic nephritic syndrome, nephrotic syndrome and other well-characterized entities. However, two critical issues require attention [1]: geographic variations in medical coding practices may lead to case underreporting, particularly in a subset of early-stage or asymptomatic patients who may be omitted from surveillance systems [2,86,87] insufficient granularity in pathological subtype classification (such as the lack of a dedicated category for IgA nephropathy) may result in underestimation of subtype-specific disease burdens. Due to the broad granularity of ICD-10 codes, distinct glomerular disease subtypes are often aggregated, highlighting the need for more refined classification systems to enhance the accuracy of burden-of-disease assessments. Taking IgA nephropathy as an exemplar – this condition represents the most prevalent primary glomerulonephritis globally, demonstrating distinctive clinical characteristics: peak incidence occurs between 20 and 40 years [88], with 30–40% of patients progressing to ESKD within 20–30 years [89]. However, current classification systems fail to designate IgA nephropathy as a distinct subtype, potentially merging its epidemiological data with broader categories such as recurrent hematuria or nephrotic syndrome, thereby systematically obscuring critical variations in pathogenic mechanisms and prognostic heterogeneity. Third, inequitable global distribution of healthcare resources – particularly the limited histopathological diagnostic capacity in low-income countries – constitutes a potential confounding factor. Fourth, the lack of provincial and urban-rural data in China did not allow us to conduct a more detailed analysis of the CKD burden across different regions of China. We only analyzed the overall disease burden for mainland China (excluding Taiwan). Future research should prioritize prospective cohort studies with detailed immunological profiling to elucidate causal pathways of specific glomerulonephritis subtypes, while establishing an international renal pathology registry that integrates biomarker data and electronic health records, developing multimodal validation frameworks using subnational datasets, and constructing subtype-specific refined assessment models, thereby enhancing diagnostic specificity and epidemiological precision.

## Conclusion

We employed the GBD 2021 repository, integrating core indicators such as incidence, prevalence, mortality, DALYs, YLDs, YLLs, and EAPC to conduct a systematic trends analysis of glomerulonephritis-induced CKD burden across global and Chinese populations during the 1990–2021 period. As the first study to compare global and Chinese CKD trends using age-period-cohort (APC) and decomposition analyses, our findings revealed that although the overall disease burden in China declined from 1990 to 2021, it remained substantial among the elderly. Notable results included a lower disease burden in China compared to the global average, significant sex disparities with higher rates in males, and an increasing burden among the elderly driven by population aging – insights further clarified by our decomposition analysis. Altogether, targeted prevention and management strategies, particularly for the elderly, are essential to reduce CKD incidence and mortality and mitigate its public health impact in China.

## Supplementary Material

Supplementary Table 5.xls

Supplementary Table 4.xls

Supplementary Table 7.docx

supplementary figures and sub supplementary figures.zip

figures and sub figures.zip

Supplementary Table 6.xls

Supplementary Table 1.docx

Supplementary Table 2.docx

Supplementary Table 3.xls

## Data Availability

Data were obtained from the GBD 2021 database *via* the IHME GBD Results Tool (https://ghdx.healthdata.org/gbd-results-tool), which provides estimates for 371 diseases and injuries across 204 countries from 1990 to 2021. The analysis code is available on GitHub (https://github.com/HTX2023/GlobalBurdenDisease).

## Data availability statement

All data that support the findings of this study are available from the corresponding author, upon reasonable request.
