# Supplementary material for: Global and China trends in glomerulonephritis-induced chronic kidney disease: health inequities, risk factors and projections to 2050
Source: Ren Fail. 2025 Oct 15;47(1):2564373. doi: 10.1080/0886022X.2025.2564373 (PMC12532362; doi:10.1080/0886022X.2025.2564373)
Supplement: Supplementary Table 7.docx [file IRNF_A_2564373_SM4376.docx]

STROBE Statement—Checklist of items that should be included in reports of ***cross-sectional studies***

|  | Item No | Recommendation |
| --- | --- | --- |
| **Title and abstract** | 1 | 1. Global Health Inequities in Glomerulonephritis-induced Chronic Kidney Disease: Comparative Analysis of Temporal Trends and Risk Factors between China and the World, with Decomposition and Age-Period-Cohort Effects Analysis (P1). |
|  |  | (b) This cross-sectional study analyzed 1990–2021 Global Burden of Disease data to compare glomerulonephritis-induced CKD burden between China and global populations. Age-standardized prevalence, incidence, mortality, and DALYs increased globally but decreased in China. Population aging and modifiable risks (hypertension, hyperglycemia) drove mortality trends. Despite projected declines in China’s overall burden by 2050, rising incidence among adults >45 years highlights urgent needs for targeted interventions (P2-3). |
| Introduction | | |
| Background/rationale | 2 | CKD is a leading global cause of mortality and disability, with glomerulonephritis representing a major etiology. Despite affecting 700 million individuals worldwide (2021), CKD exhibits marked regional disparities: age-standardized prevalence rates in low-middle SDI countries exceed those in high-SDI regions by 40%. In China, where CKD prevalence reaches 10.6%, glomerulonephritis accounts for 55.3% of end-stage cases, significantly higher than the United States and other Western countries. Critical evidence gaps persist regarding temporal trends, age-sex stratification of risk, and modifiable drivers (including population aging and hypertension) in high-burden populations (P4-6). |
| Objectives | 3 | This study aims to: (1) compare the 1990–2021 disease burden (prevalence, incidence, mortality, DALYs) of glomerulonephritis-induced CKD between China and global populations; (2) quantify age-sex disparities and attributable risk factors through decomposition and age-period-cohort analyses; (3) project future trajectories using Bayesian forecasting to prioritize interventions for high-risk subgroups (P6-7). |
| Methods | | |
| Study design | 4 | This cross-sectional comparative study analyzed 1990–2021 data from the GBD 2021 database to assess temporal trends and risk factors of glomerulonephritis-induced CKD in China versus global populations (P6-7). |
| Setting | 5 | Data spanned 204 countries/regions globally, with China as the primary focus. Data collection periods: 1990–2021 (recruitment/exposure follow-up ended in December 2021) (P6-7). |
| Participants | 6 | Aggregated population-level data were sourced from GBD 2021, including cohort studies, national surveys, hospital records, and vital registries. Individual participant selection criteria are described in original GBD studies (P6-7). |
| Variables | 7 | Outcomes: Incidence, prevalence, mortality, DALYs, YLDs, YLLs (P7).  Exposures: SDI, dietary/behavioral/metabolic risk factors (15 variables, P8). ​  Confounders: Age, sex (P7). Diagnostic criteria detailed in Supplementary Table 2. |
| Data sources/ measurement | 8* | Data extracted from GBD 2021 (https://ghdx.healthdata.org). Age-standardized rates (ASRs) computed using GBD’s global reference population. Stratified analysis by sex and SDI. Measurement comparability ensured via standardized ICD-10 coding and GBD protocols (P6-8). |
| Bias | 9 | Uncertainty intervals (UIs) quantified data variability. Multiple data sources minimized selection bias. Residual biases may arise from heterogeneous diagnostic standards across regions and retrospective data limitations (P6-10). |
| Study size | 10 | Study size determined by GBD’s global coverage (204 countries/regions, 1990–2021). Sample size calculations not applicable to ecological analyses (P6). |
| Quantitative variables | 11 | Continuous variables (e.g., age) categorized into standard GBD age groups (e.g., <45, 45–64, ≥65 years). Risk factors classified as dichotomous (present/absent) based on GBD comparative risk assessment framework. Temporal trends analyzed via EAPC (P6-10). |
| Statistical methods | 12 | (*a*) EAPC with 95% UI for trend analysis (P7-8); |
|  |  | (*b*) Age-period-cohort (APC) Poisson models (P8); |
|  |  | (*c*) Gupta decomposition for demographic/epidemiological drivers (P9); |
|  |  | (*d*) Bayesian APC forecasting (2021–2050) (P9-10); ​ |
|  |  | (*e*) Slope Index of Inequality (SII) and Concentration Index (CI) for health disparities. Analyses performed in R 4.3.3 and JD_GBDR V2, with significance at p<0.05 (P10). |
| **Results** | | |
| Participants | 13* | (a) Stage-specific participant counts (P11-18): (1) 1.61 million prevalent cases of glomerulonephritis-induced CKD in China (2021), with 876,650 males and 733,638 females. (2) Mortality analysis included 5,320 deaths (2,968 males, 2,351 females). (3) Full cohort coverage: 204 countries/regions (1990–2021) (Table 1). |
|  |  | (b) Reasons for exclusion (P11-18): (1) Data gaps in <2% of regions addressed via multiple imputation. (2) Cases with inconsistent diagnostic criteria were recalibrated using ICD-10 codes (Supplementary Table 2). |
|  |  | (c) As this study analyzed secondary aggregated data from the Global Burden of Disease (GBD) database, a traditional participant flow diagram (e.g., enrollment, exclusion, or follow-up stages) is not applicable. GBD synthesizes data from population-level sources (e.g., surveys, registries, cohort studies) using standardized modeling frameworks, rather than recruiting individual participants. |
| Descriptive data | 14* | 1. Demographic characteristics (P11-18): (1) Higher ASPR in males (102.94 vs. 85.37/100,000, p<0.01). (2) Age stratification:   1) Peak incidence in 0–4 years (ASIR=4.26/100,000).   2) Mortality concentrated in 65–89 years (ASDR=0.30/100,000). |
|  |  | 1. Missing data: (1) No variable-specific missingness reported (GBD sources >95% complete). 2. Cross-region comparability validated via SDI stratification (Figure 2). |
| Outcome data | 15* | Key outcomes (P11-31): (1) Deaths: 5,320 (ASDR=0.30/100,000, 95% UI: 0.22–0.39). (2) DALYs: 307,228 person-years (male: 20.48/100,000; female: 15.70/100,000). (3) YLDs/YLLs ratio: 48.5%/51.5% (Table 1). |
| Main results | 16 | (a)Trends (P11-31): (1) ASPR declined by 14.02% in China (EAPC=−0.39) vs. 1.09% global increase (Table 1). (2) Adjusted male mortality risk: RR=1.17 (1997–2002 cohort, 95% CI: 1.12–1.22). |
|  |  | (b) Continuous variable categorization: (1) Age standardization using GBD global reference population. (2) SDI quartile boundaries defined in Methods. |
|  |  | 1. Absolute risk translation:  Lifetime CKD risk in 70–74 years: 2.1% (male) vs. 1.4% (female). |
| Other analyses | 17 | (1) Subgroup analyses: Sex disparity: 20.6% higher ASPR in males (p<0.001) (Table 1). SDI stratification: Low-SDI regions had 2.3× higher DALY rates (Figure 2). (2) Sensitivity analyses: BAPC model validation: <8% prediction error (Figure 7). Demographic decomposition: Population growth contributed −125.58% to incidence decline (Figure 6). |
| Discussion | | |
| Key results | 18 | (1)China's glomerulonephritis-induced CKD burden (ASPR 1.65 vs global 2.25 per 100,000) remained below global averages from 1990-2021. (2) Significant gender disparity: Higher male ASIR and ASDR (0.21 vs 0.14 per 100,000). (3) Bimodal age distribution: Peak incidence in 1-4-year-olds (5.63/100,000), with ≥60-year-olds contributing 62.4% of DALYs. (4) APC analysis revealed +0.51% annual net drift (95% CI:0.15-0.86) in 45-75-year-olds. (5) BAPC forecasts 2022-2050: 18.7% overall incidence decline but 23.5% rise in ≥45-year-olds. |
| Limitations | 19 | 1. GBD model-dependent estimates may undercount early-stage cases. 2. ICD-10 classification merges pathological subtypes (e.g., no dedicated IgA nephropathy code), masking etiological heterogeneity. 3. Lack of provincial/urban-rural stratification. 4. Unmeasured infection-related risks (streptococcal/HBV/HCV links) in aging populations. 5. Assumed care homogeneity contradicts real-world disparities. |
| Interpretation | 20 | China's success in reducing CKD burden reflects tiered prevention systems and universal dialysis coverage. However, aging demographics demand recalibrated strategies. Gender disparities justify male-specific interventions (e.g., annual ACR  screening for smoking males >40). Childhood incidence peaks necessitate perinatal renal monitoring protocols. Metabolic dominance supports integrating CKD into national NCD control priorities. |
| Generalisability | 21 | Applicable to middle-income countries undergoing similar demographic transitions |
| Other information | | |
| Funding | 22 | Specify the funding source: National Natural Science Foundation of China (Grant number: 82360153) Identify the grant recipient: Dr Tian |

**Note:** An Explanation and Elaboration article discusses each checklist item and gives methodological background and published examples of transparent reporting. The STROBE checklist is best used in conjunction with this article (freely available on the Web sites of PLoS Medicine at http://www.plosmedicine.org/, Annals of Internal Medicine at http://www.annals.org/, and Epidemiology at http://www.epidem.com/). Information on the STROBE Initiative is available at www.strobe-statement.org.
