# Supplementary figures and images for: Global and China trends in glomerulonephritis-induced chronic kidney disease: health inequities, risk factors and projections to 2050
Source: Ren Fail. 2025 Oct 15;47(1):2564373. doi: 10.1080/0886022X.2025.2564373 (PMC12532362; doi:10.1080/0886022X.2025.2564373)

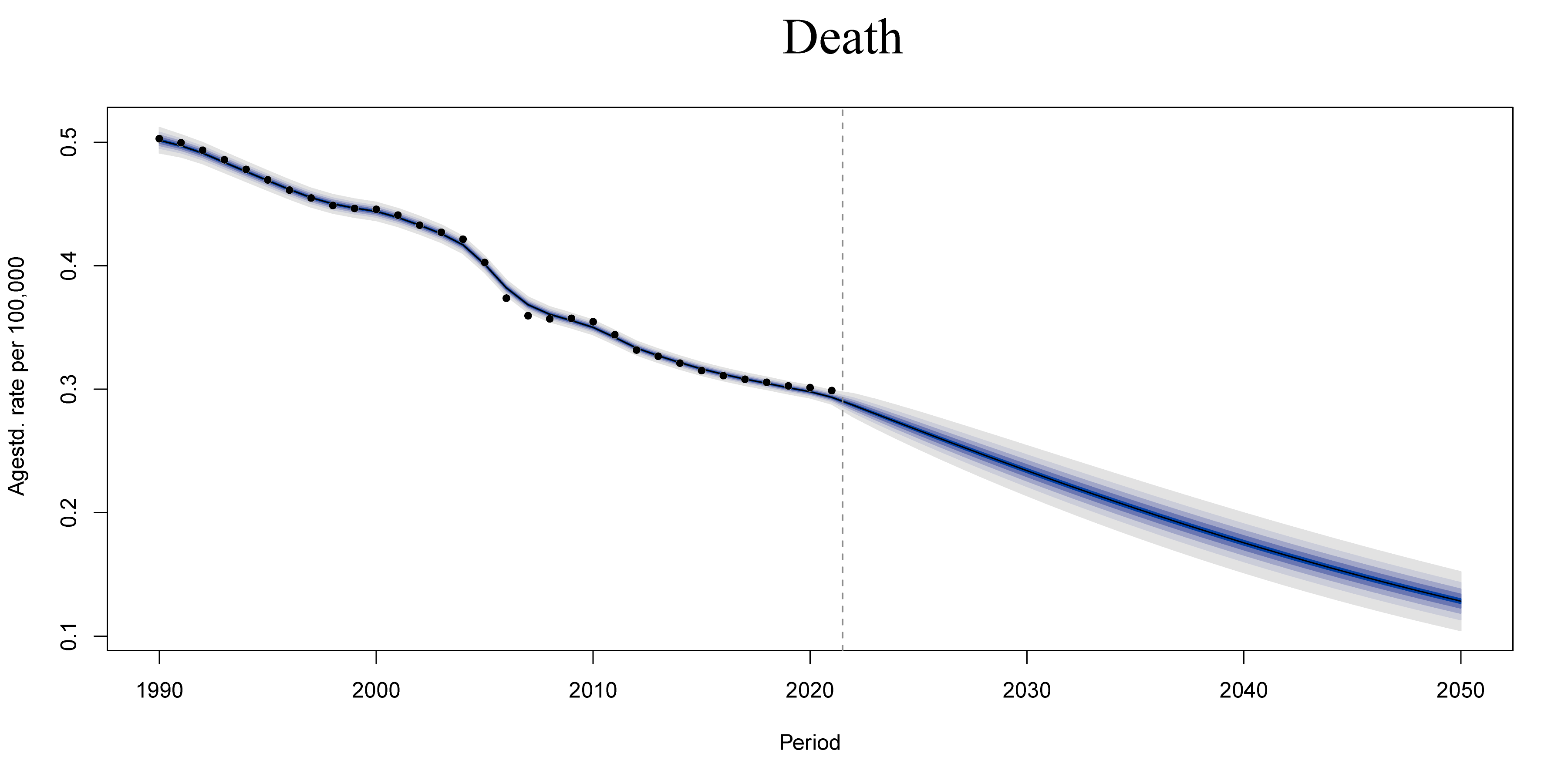

Supplement: supplementary figures and sub supplementary figures.zip [file IRNF_A_2564373_SM4375.zip › supplementary figures and sub supplementary figures/sub supplementary figures/supplementary figure11A.tif]

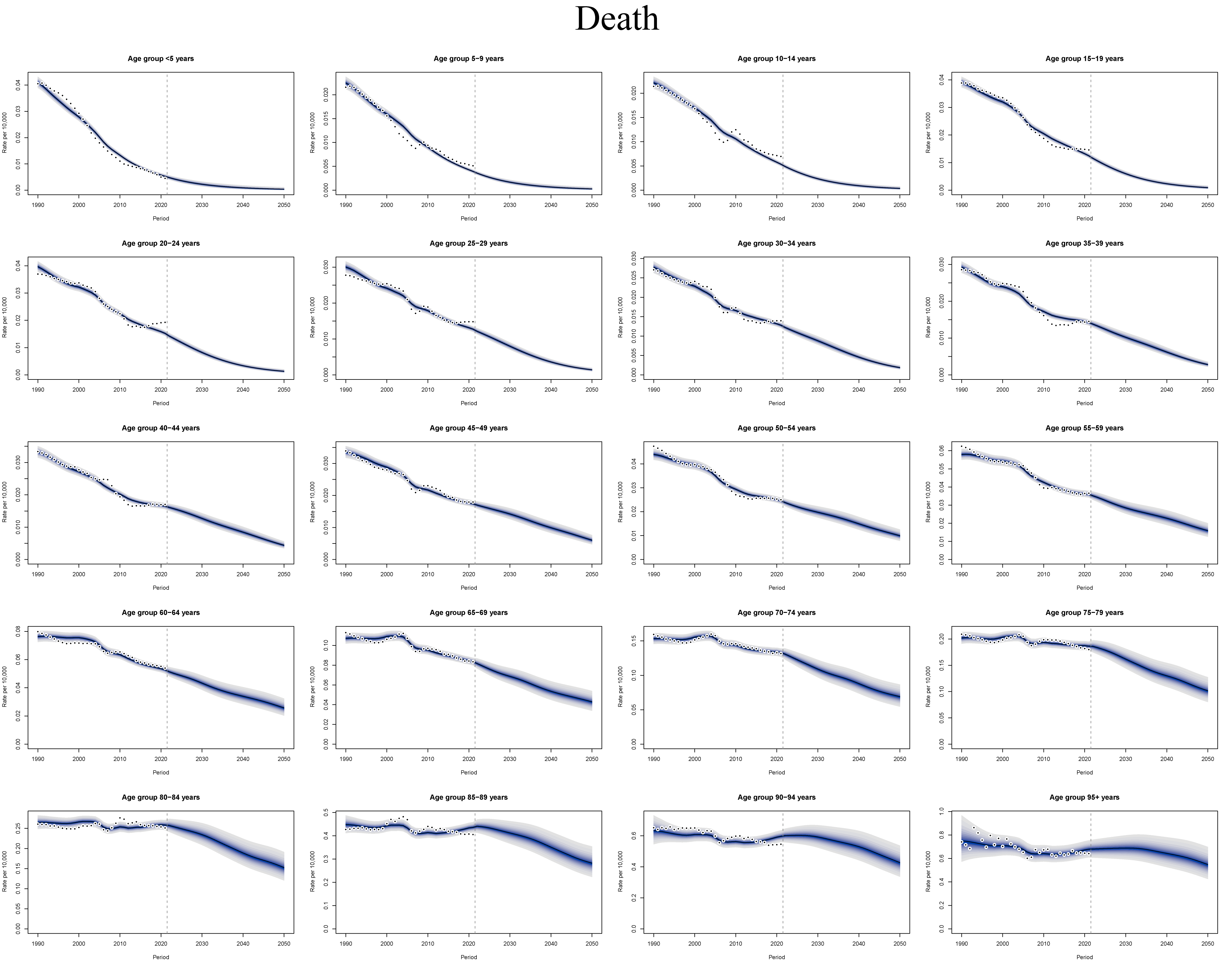

Supplement: supplementary figures and sub supplementary figures.zip [file IRNF_A_2564373_SM4375.zip › supplementary figures and sub supplementary figures/sub supplementary figures/supplementary figure11B.tif]

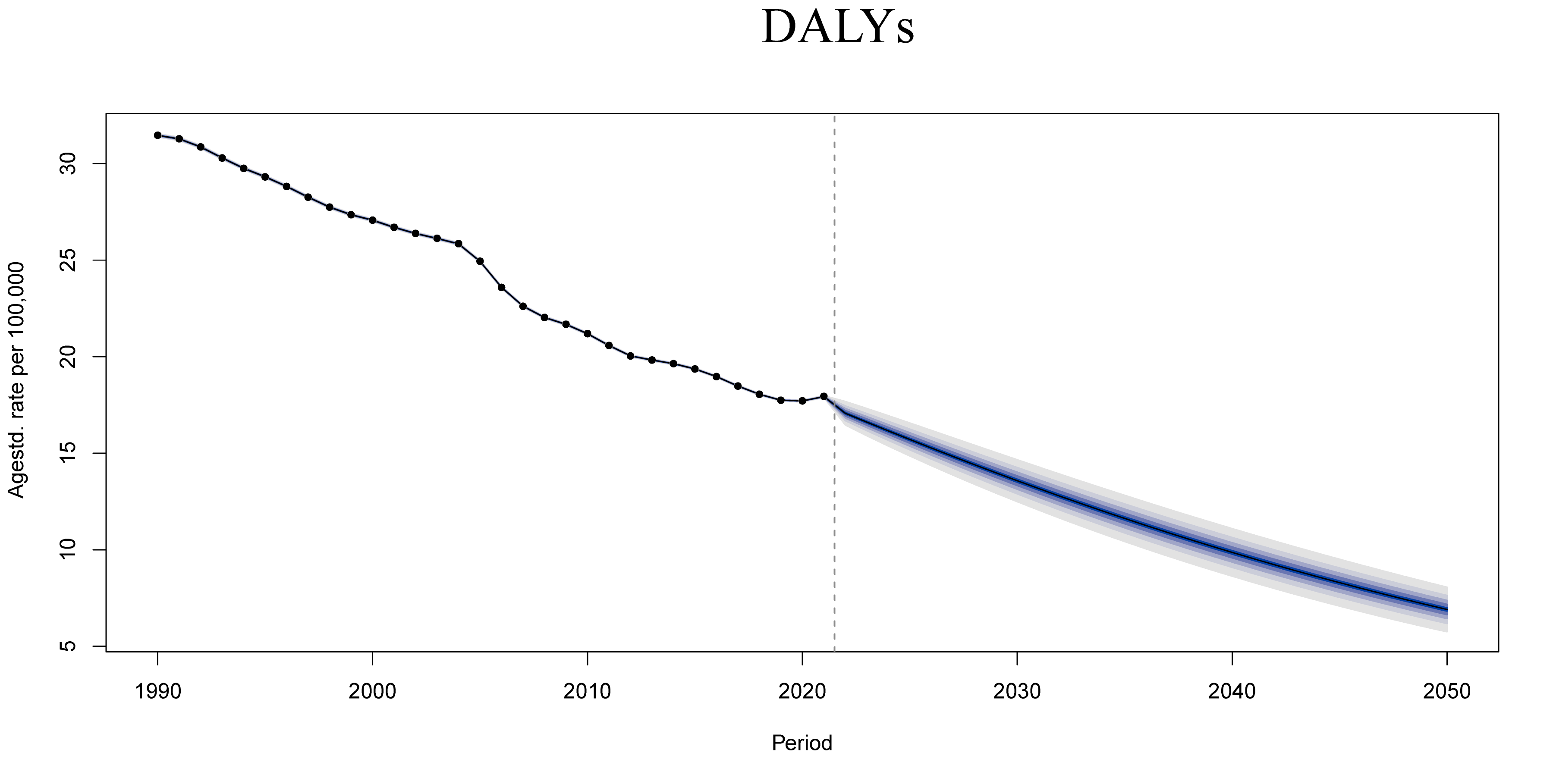

Supplement: supplementary figures and sub supplementary figures.zip [file IRNF_A_2564373_SM4375.zip › supplementary figures and sub supplementary figures/sub supplementary figures/supplementary figure12A.tif]

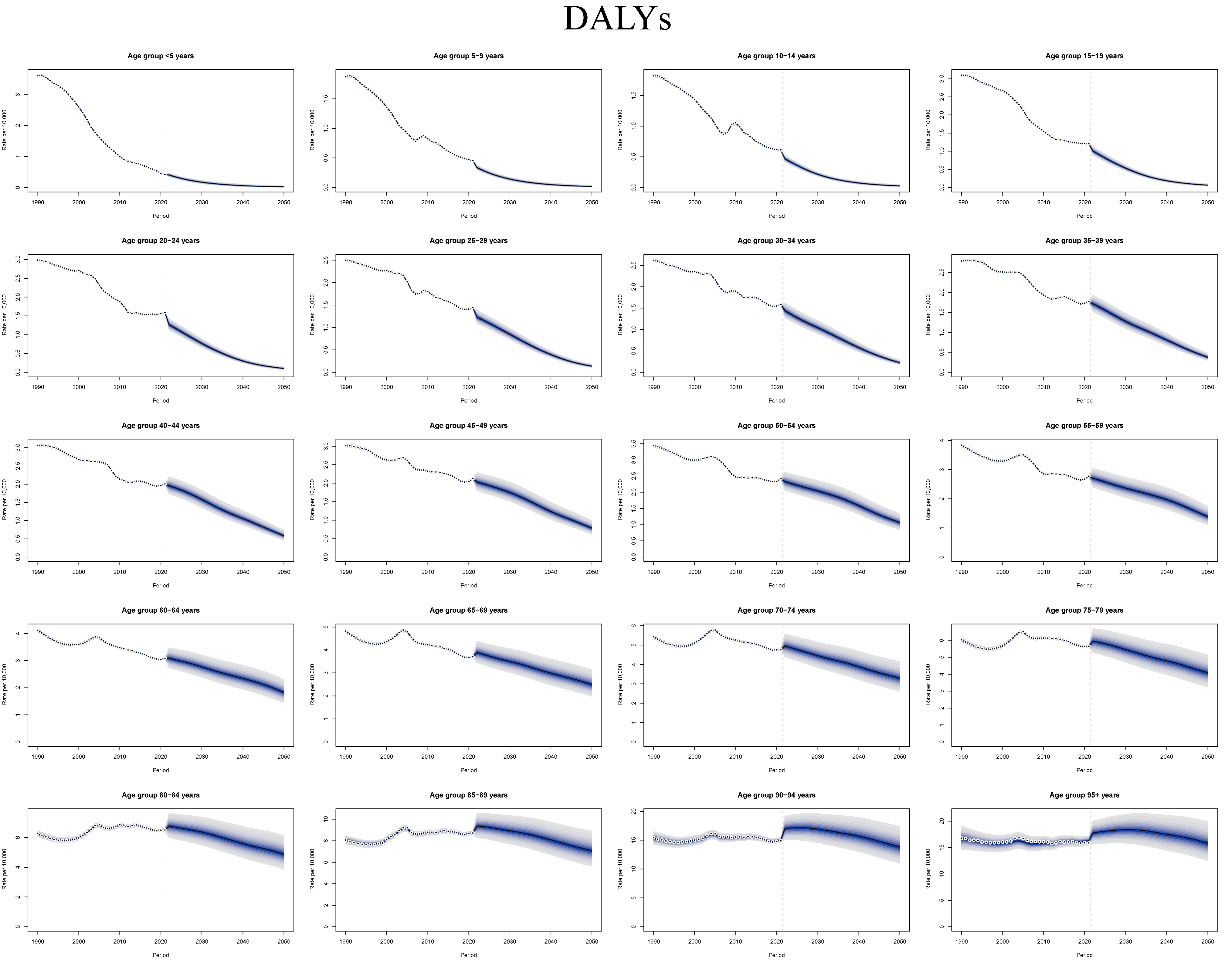

Supplement: supplementary figures and sub supplementary figures.zip [file IRNF_A_2564373_SM4375.zip › supplementary figures and sub supplementary figures/sub supplementary figures/supplementary figure12B.tif]

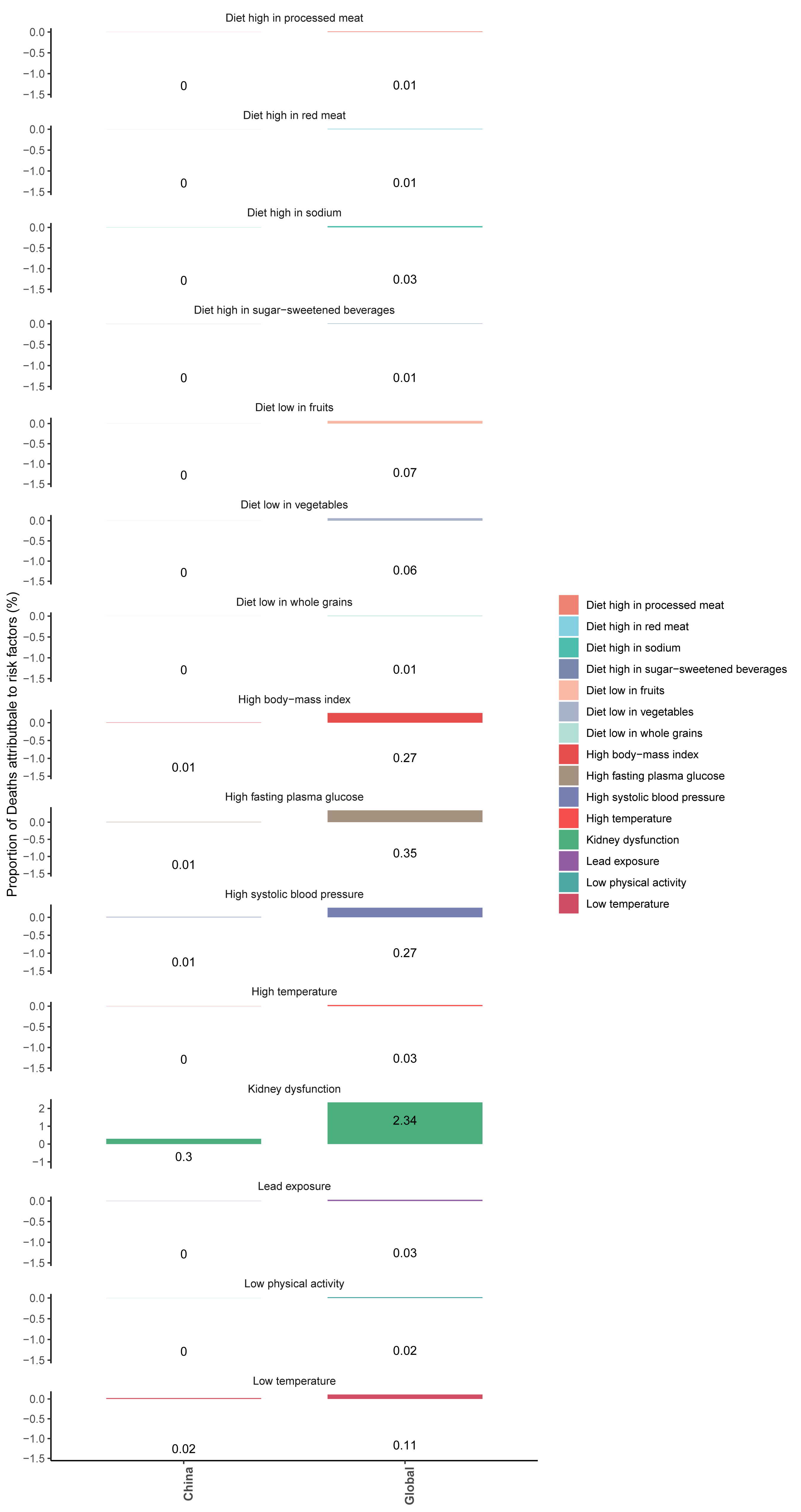

Supplement: supplementary figures and sub supplementary figures.zip [file IRNF_A_2564373_SM4375.zip › supplementary figures and sub supplementary figures/sub supplementary figures/supplementary figure13A.tif]

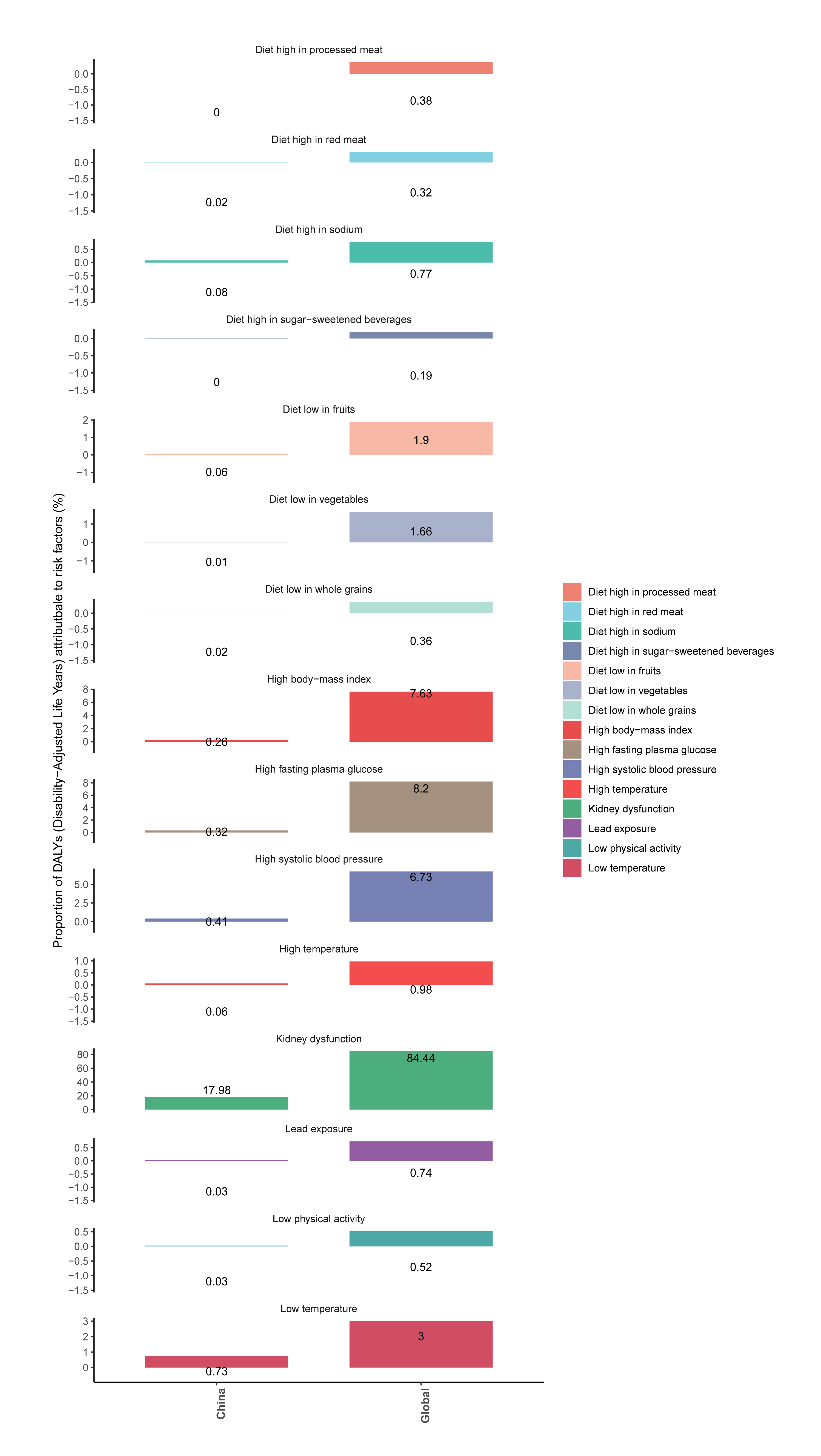

Supplement: supplementary figures and sub supplementary figures.zip [file IRNF_A_2564373_SM4375.zip › supplementary figures and sub supplementary figures/sub supplementary figures/supplementary figure13B.tif]

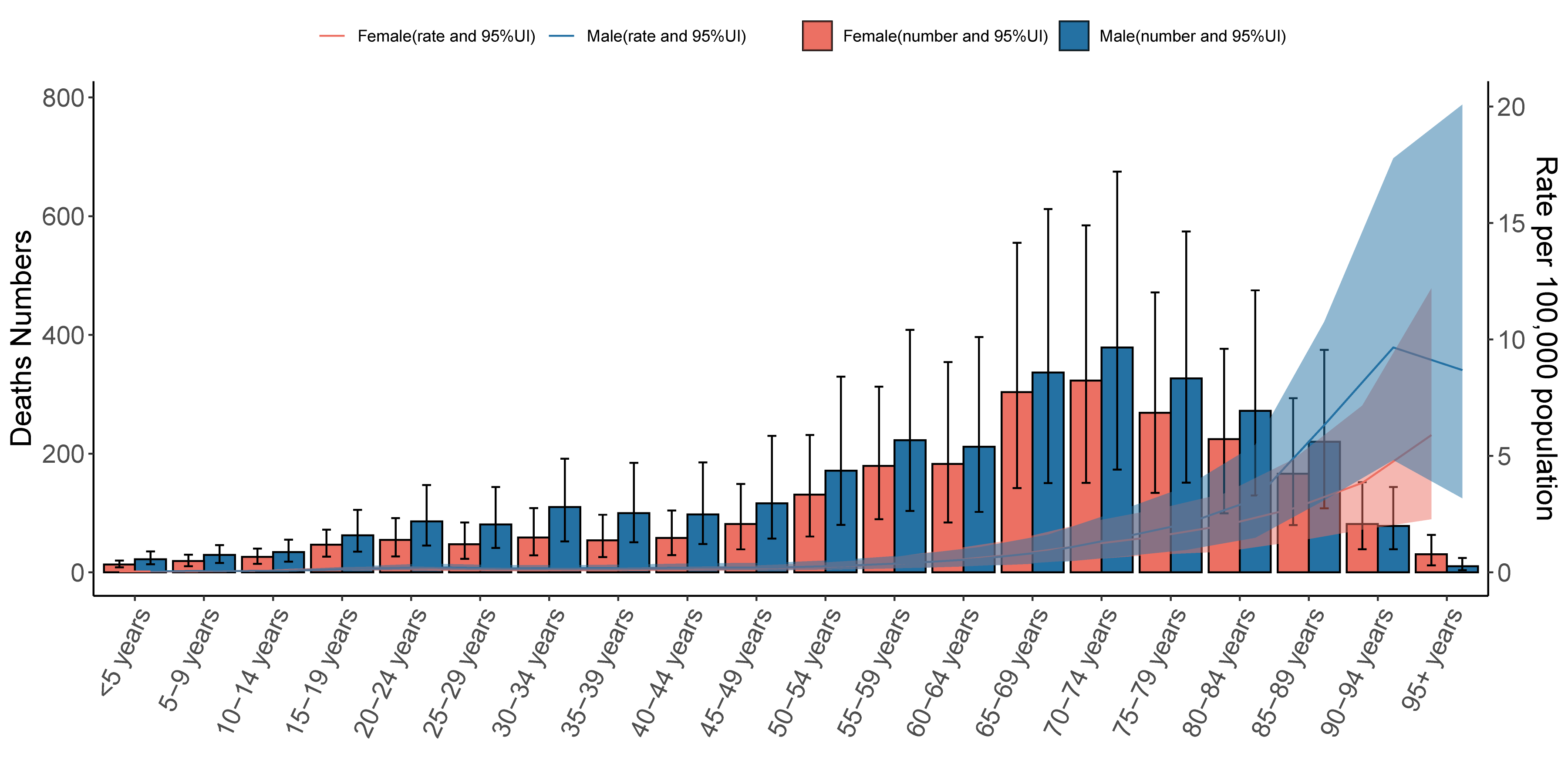

Supplement: supplementary figures and sub supplementary figures.zip [file IRNF_A_2564373_SM4375.zip › supplementary figures and sub supplementary figures/sub supplementary figures/supplementary figure1A.tif]

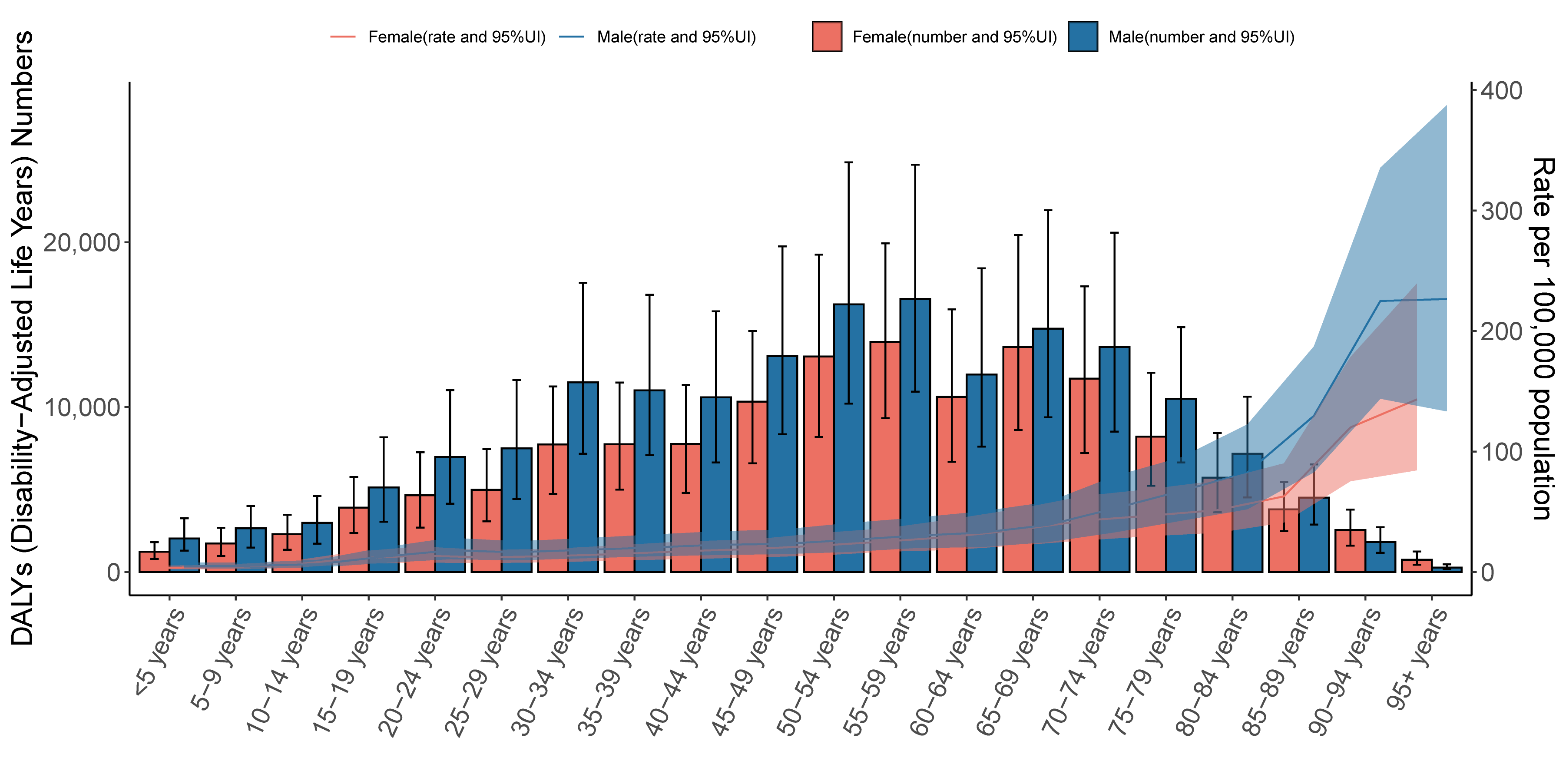

Supplement: supplementary figures and sub supplementary figures.zip [file IRNF_A_2564373_SM4375.zip › supplementary figures and sub supplementary figures/sub supplementary figures/supplementary figure1B.tif]

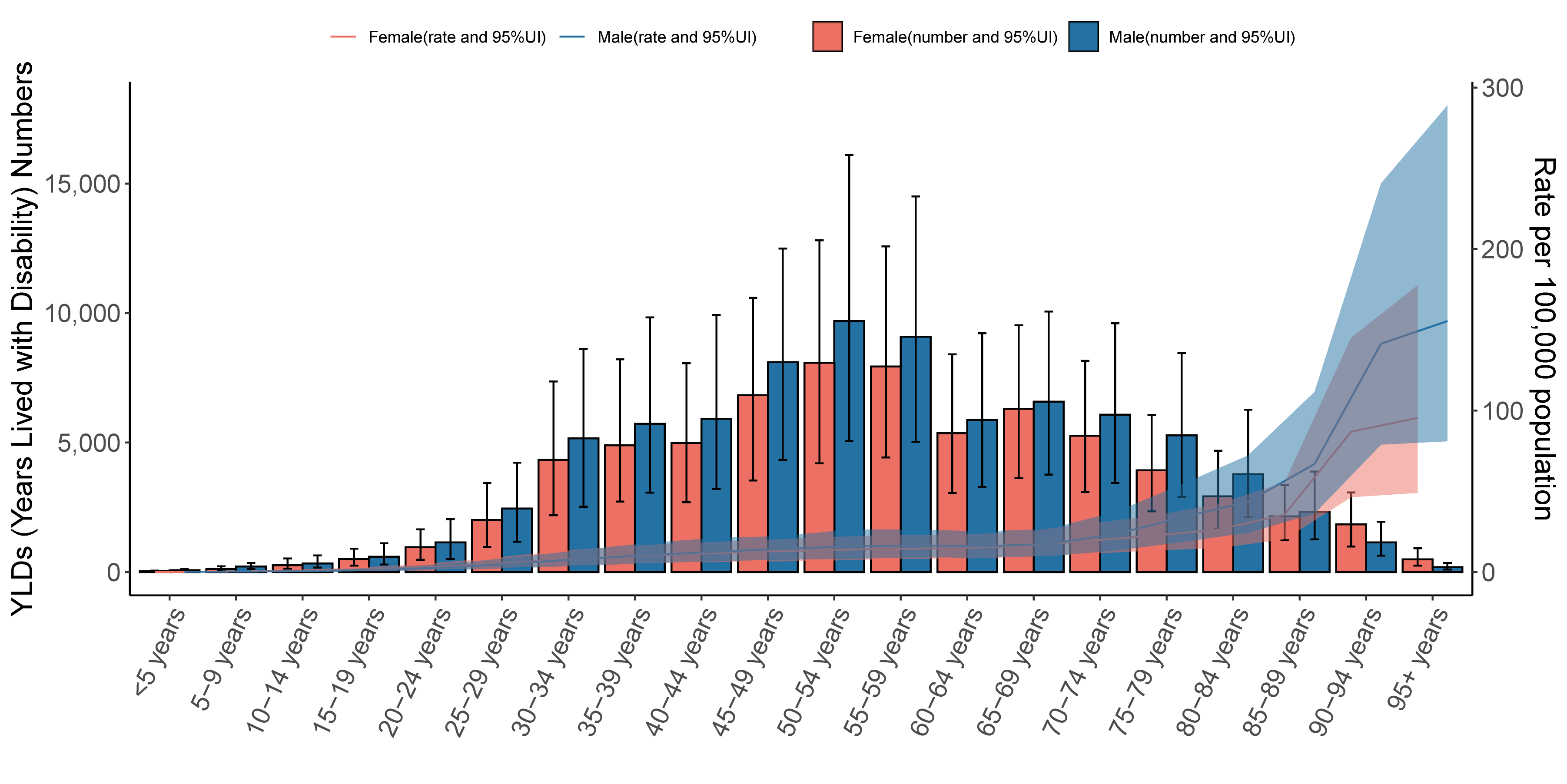

Supplement: supplementary figures and sub supplementary figures.zip [file IRNF_A_2564373_SM4375.zip › supplementary figures and sub supplementary figures/sub supplementary figures/supplementary figure2A.tif]

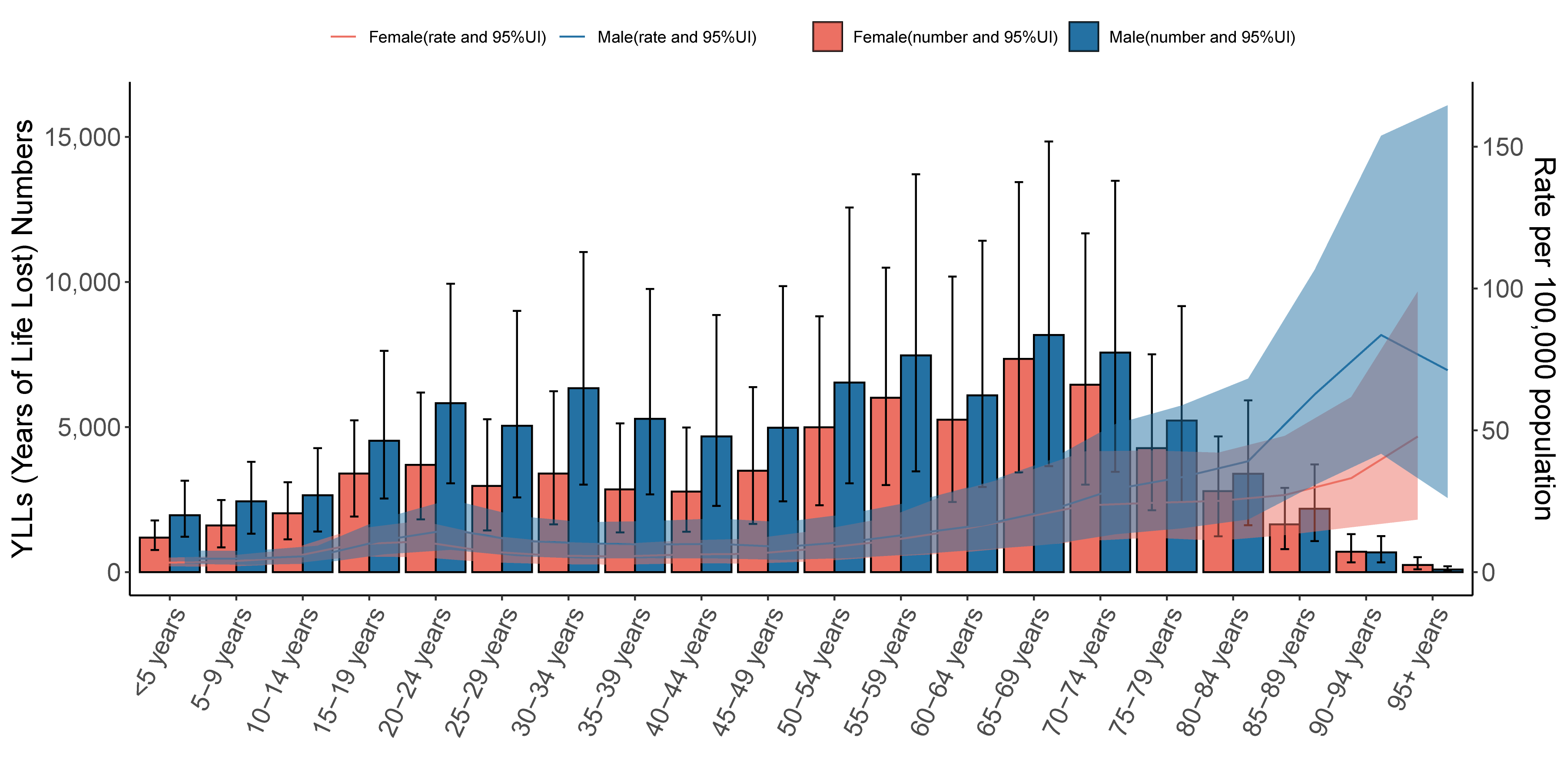

Supplement: supplementary figures and sub supplementary figures.zip [file IRNF_A_2564373_SM4375.zip › supplementary figures and sub supplementary figures/sub supplementary figures/supplementary figure2B.tif]

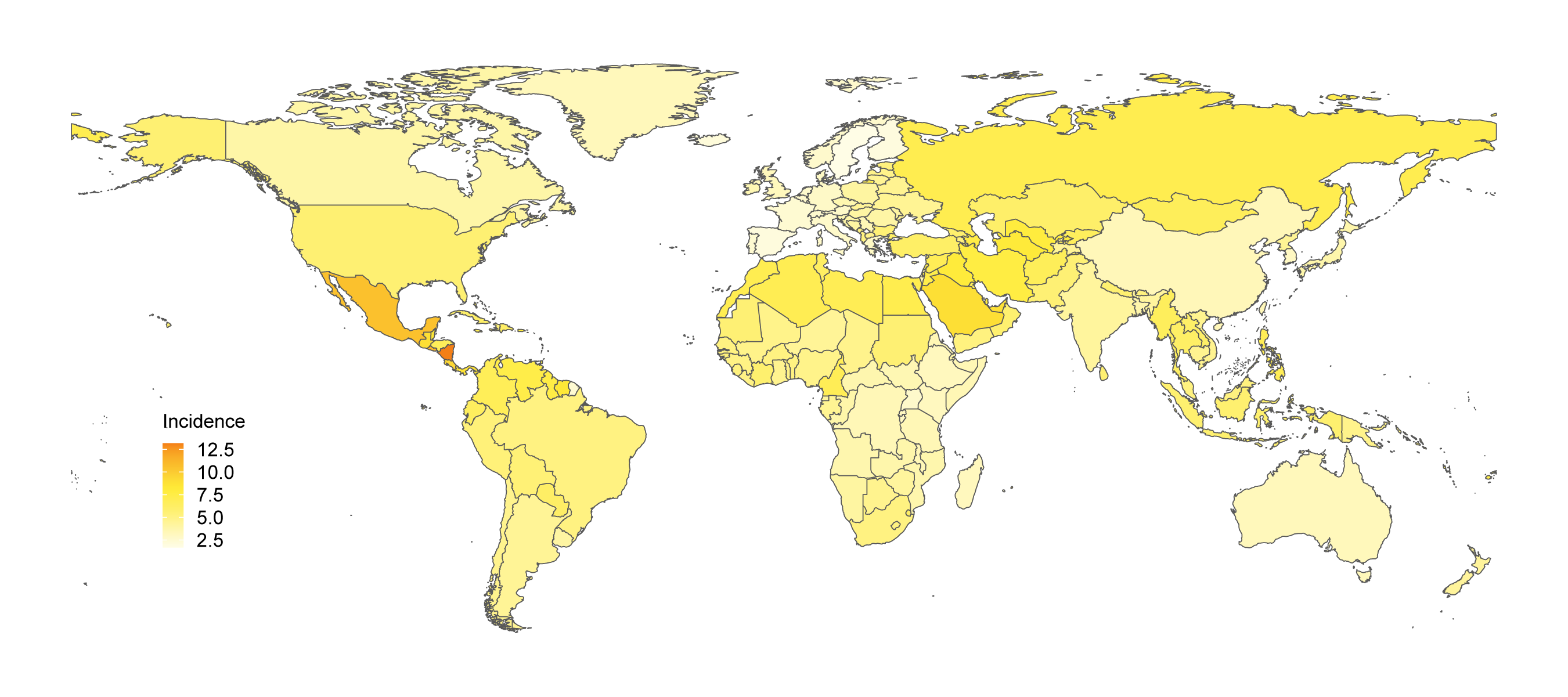

Supplement: supplementary figures and sub supplementary figures.zip [file IRNF_A_2564373_SM4375.zip › supplementary figures and sub supplementary figures/sub supplementary figures/supplementary figure3A.tif]

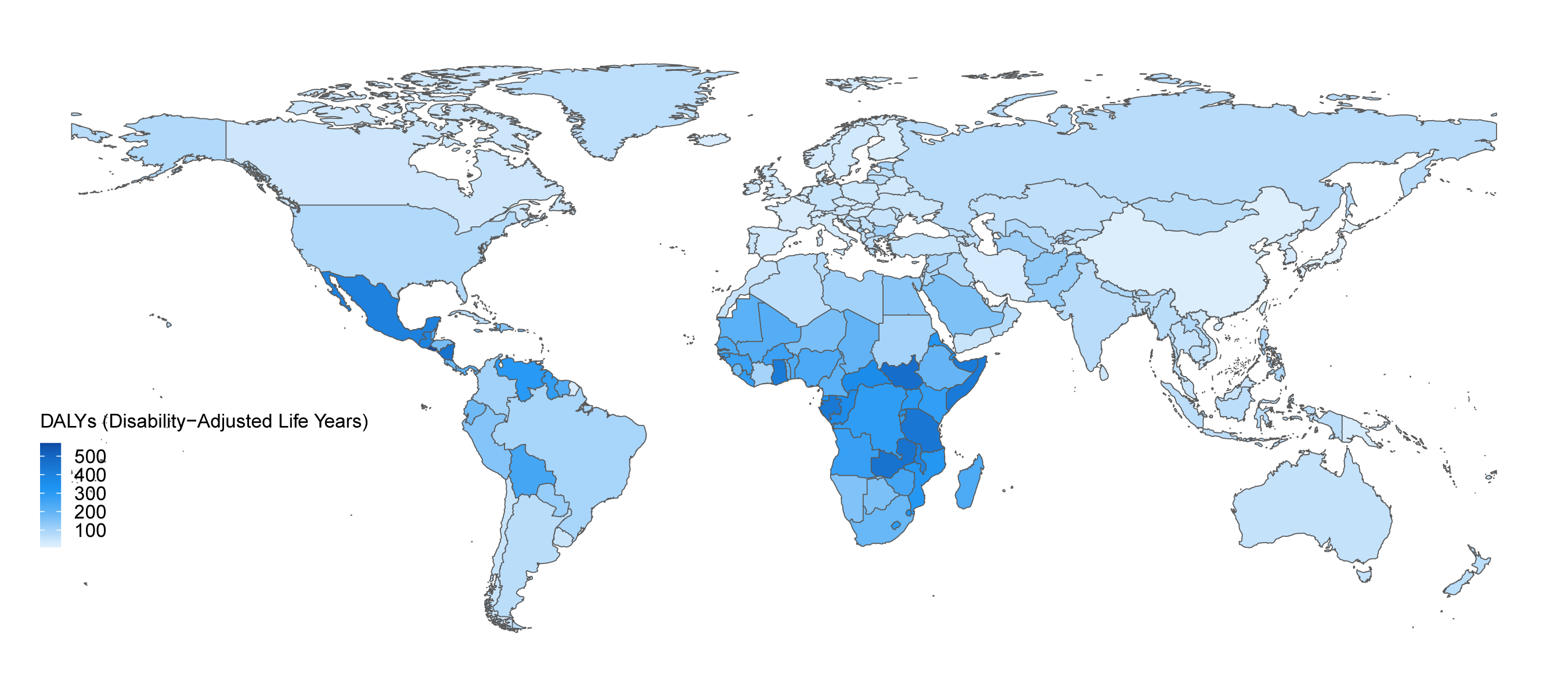

Supplement: supplementary figures and sub supplementary figures.zip [file IRNF_A_2564373_SM4375.zip › supplementary figures and sub supplementary figures/sub supplementary figures/supplementary figure3B.tif]

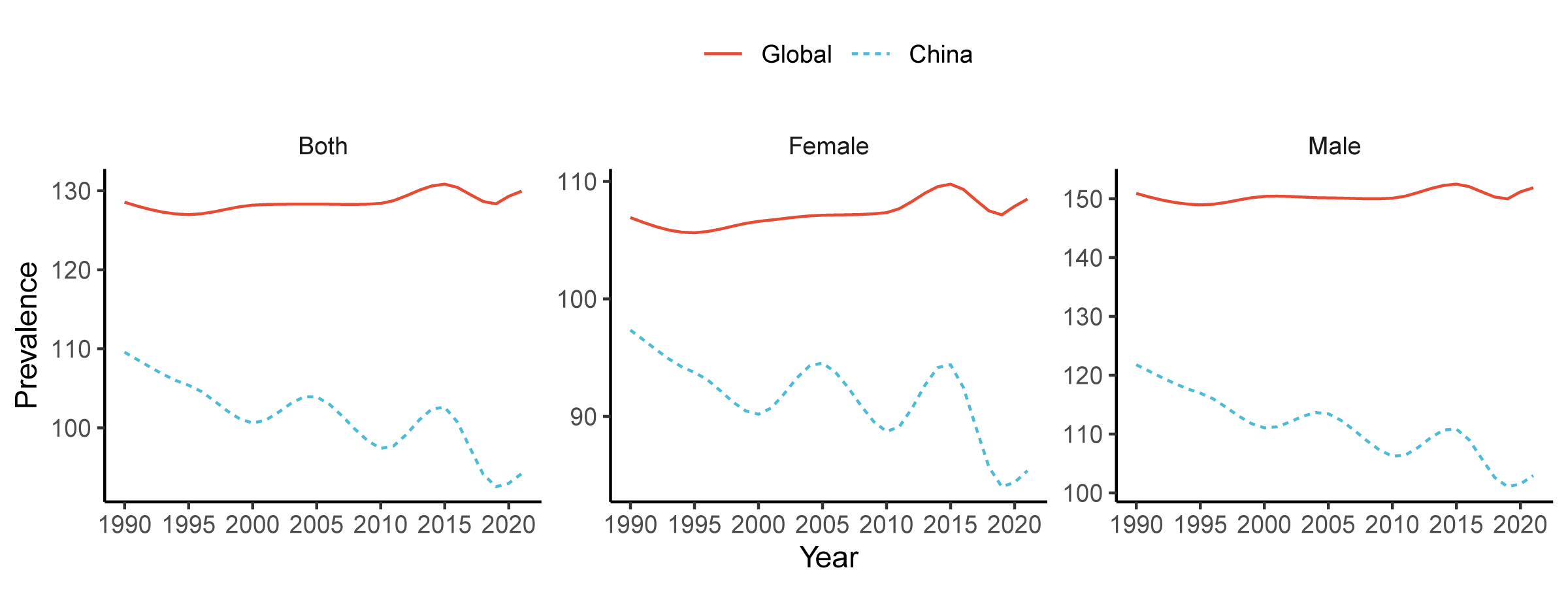

Supplement: supplementary figures and sub supplementary figures.zip [file IRNF_A_2564373_SM4375.zip › supplementary figures and sub supplementary figures/sub supplementary figures/supplementary figure7A.tif]

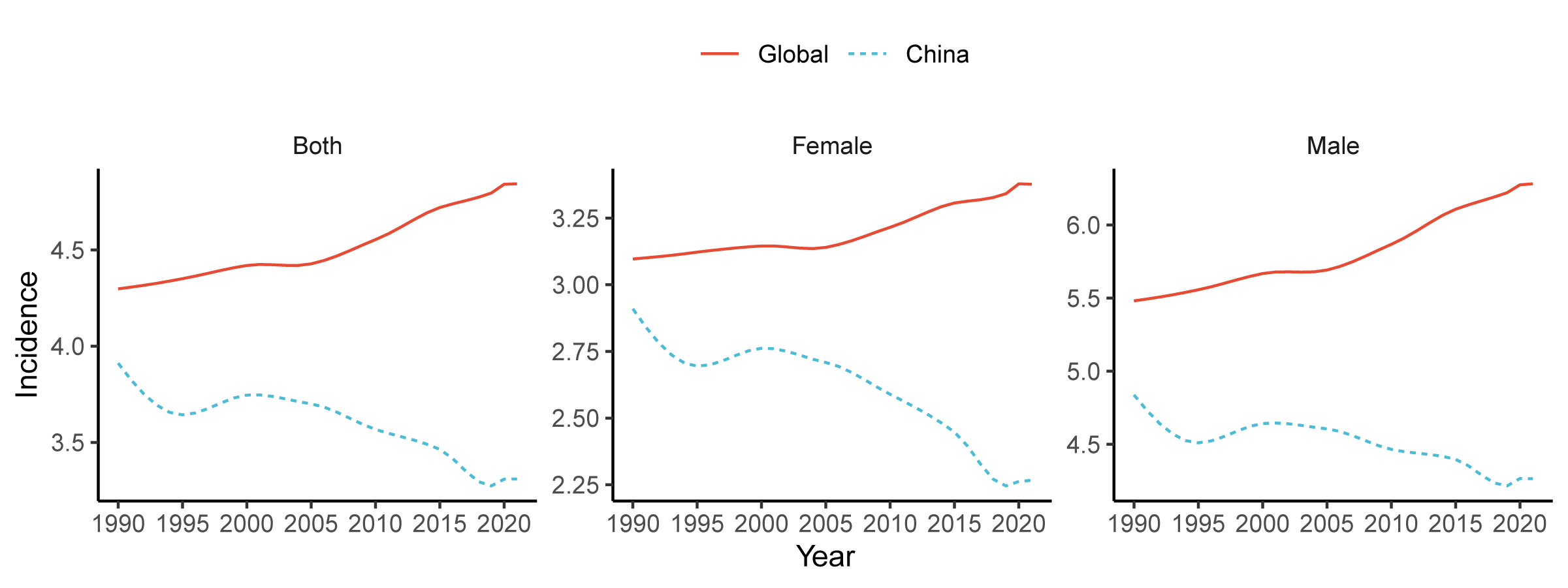

Supplement: supplementary figures and sub supplementary figures.zip [file IRNF_A_2564373_SM4375.zip › supplementary figures and sub supplementary figures/sub supplementary figures/supplementary figure7B.tif]

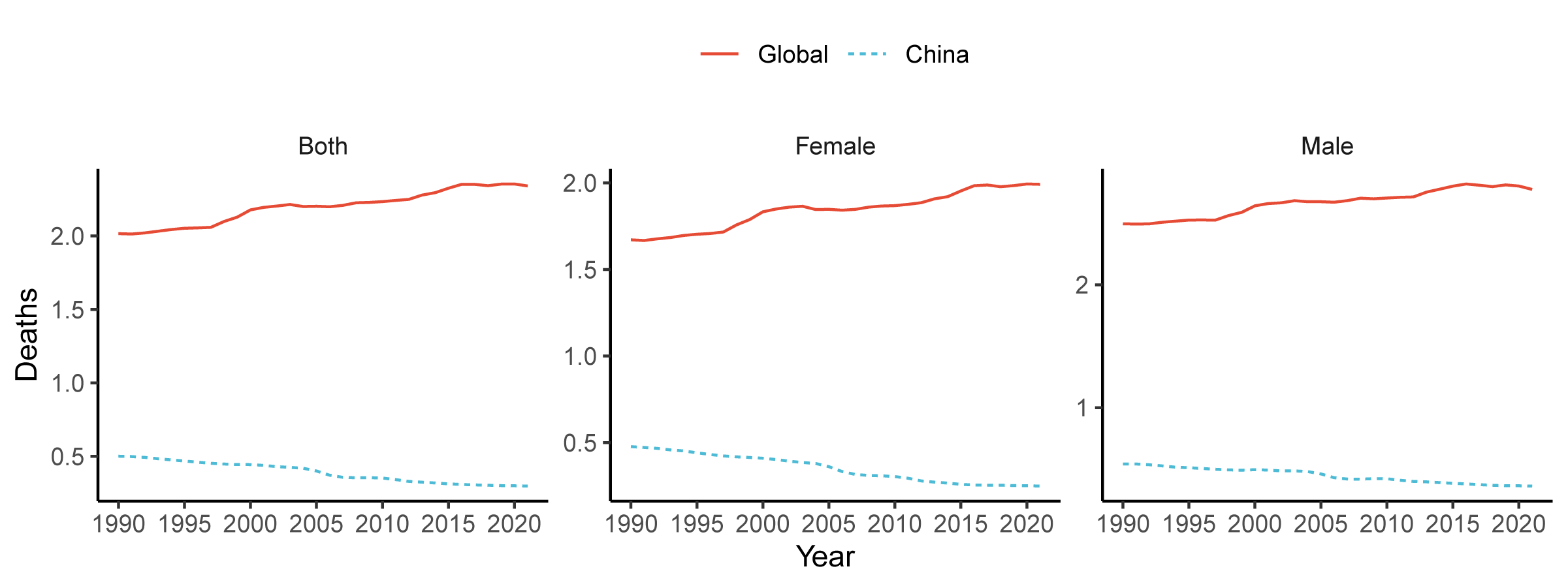

Supplement: supplementary figures and sub supplementary figures.zip [file IRNF_A_2564373_SM4375.zip › supplementary figures and sub supplementary figures/sub supplementary figures/supplementary figure7C.tif]

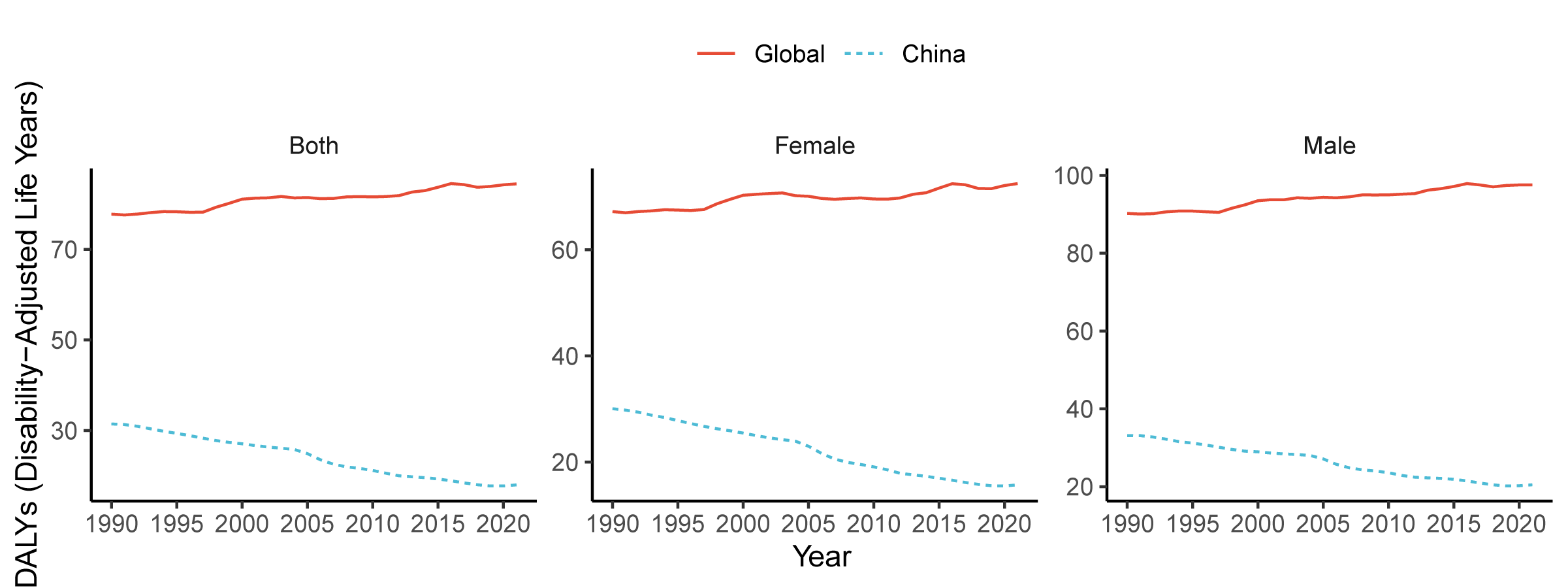

Supplement: supplementary figures and sub supplementary figures.zip [file IRNF_A_2564373_SM4375.zip › supplementary figures and sub supplementary figures/sub supplementary figures/supplementary figure7D.tif]

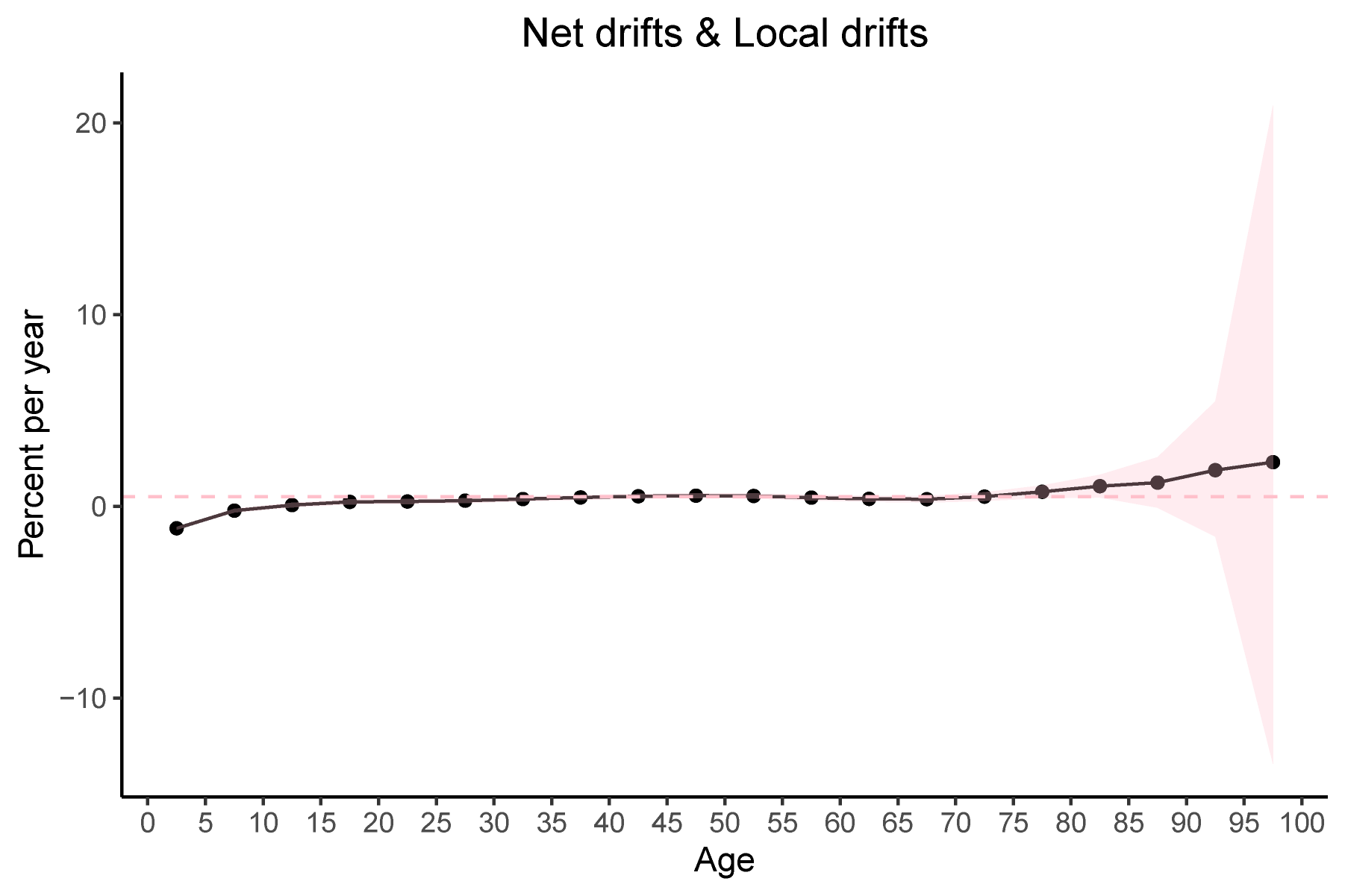

Supplement: supplementary figures and sub supplementary figures.zip [file IRNF_A_2564373_SM4375.zip › supplementary figures and sub supplementary figures/sub supplementary figures/supplementary figure8A.tif]

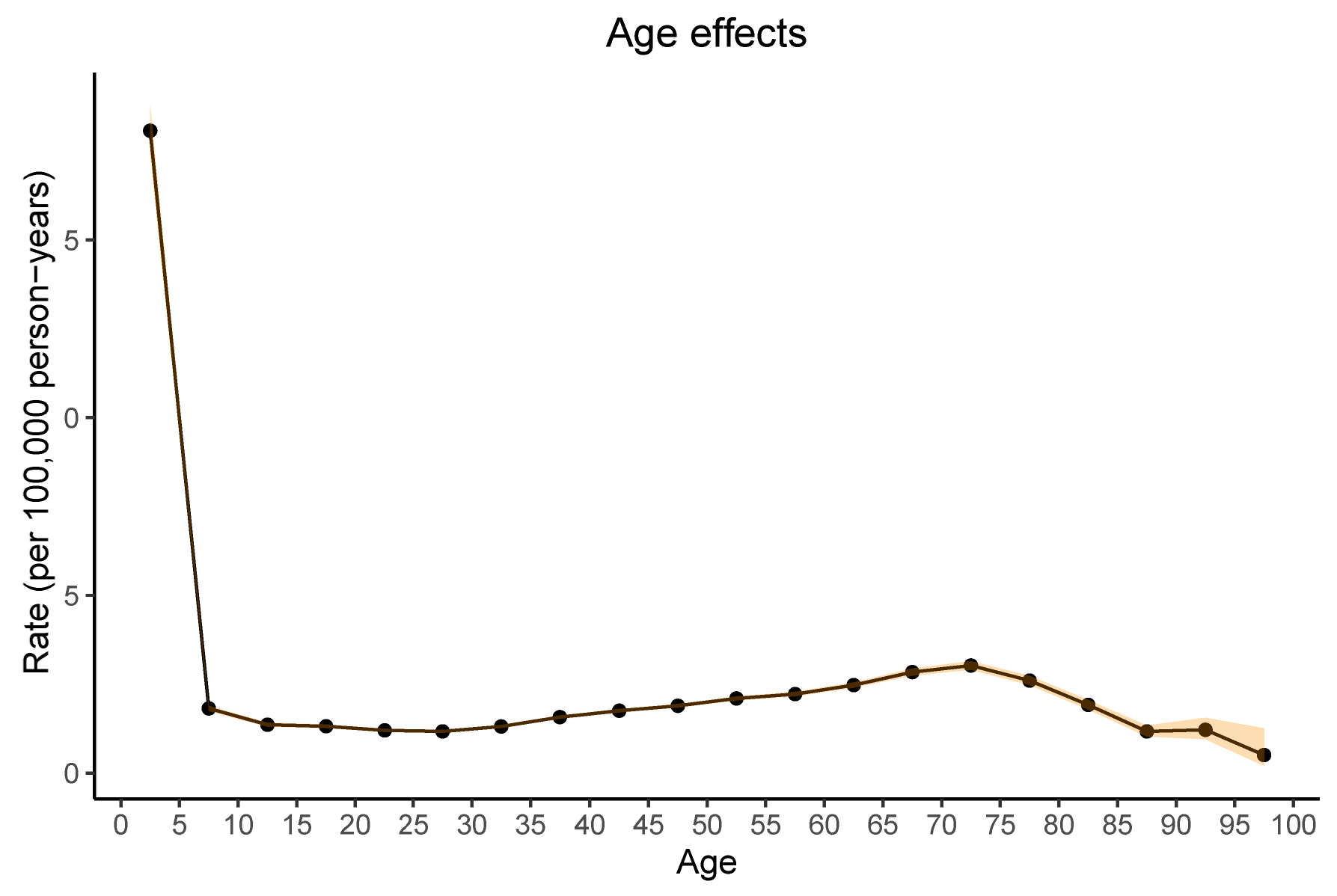

Supplement: supplementary figures and sub supplementary figures.zip [file IRNF_A_2564373_SM4375.zip › supplementary figures and sub supplementary figures/sub supplementary figures/supplementary figure8B.tif]

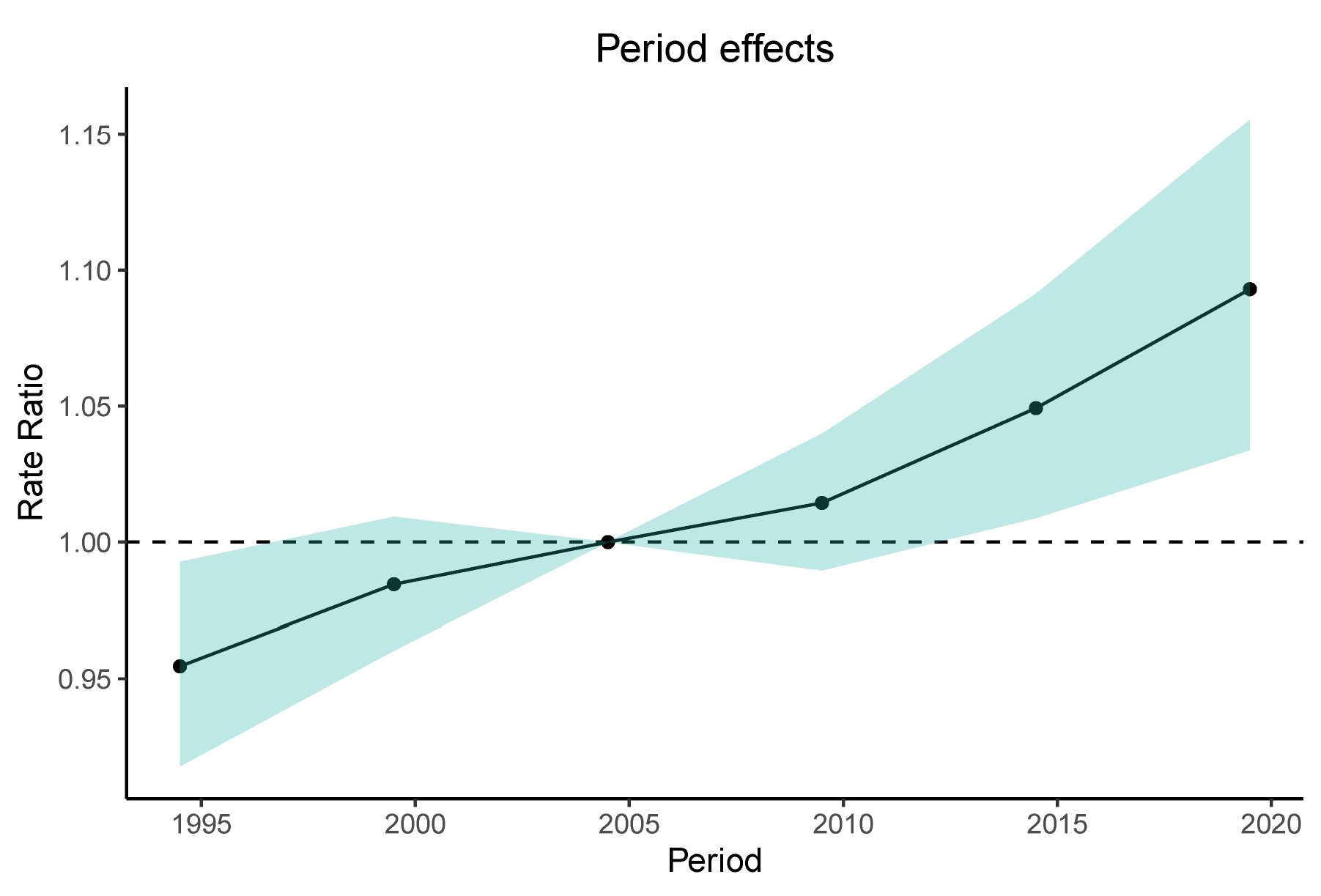

Supplement: supplementary figures and sub supplementary figures.zip [file IRNF_A_2564373_SM4375.zip › supplementary figures and sub supplementary figures/sub supplementary figures/supplementary figure8C.tif]

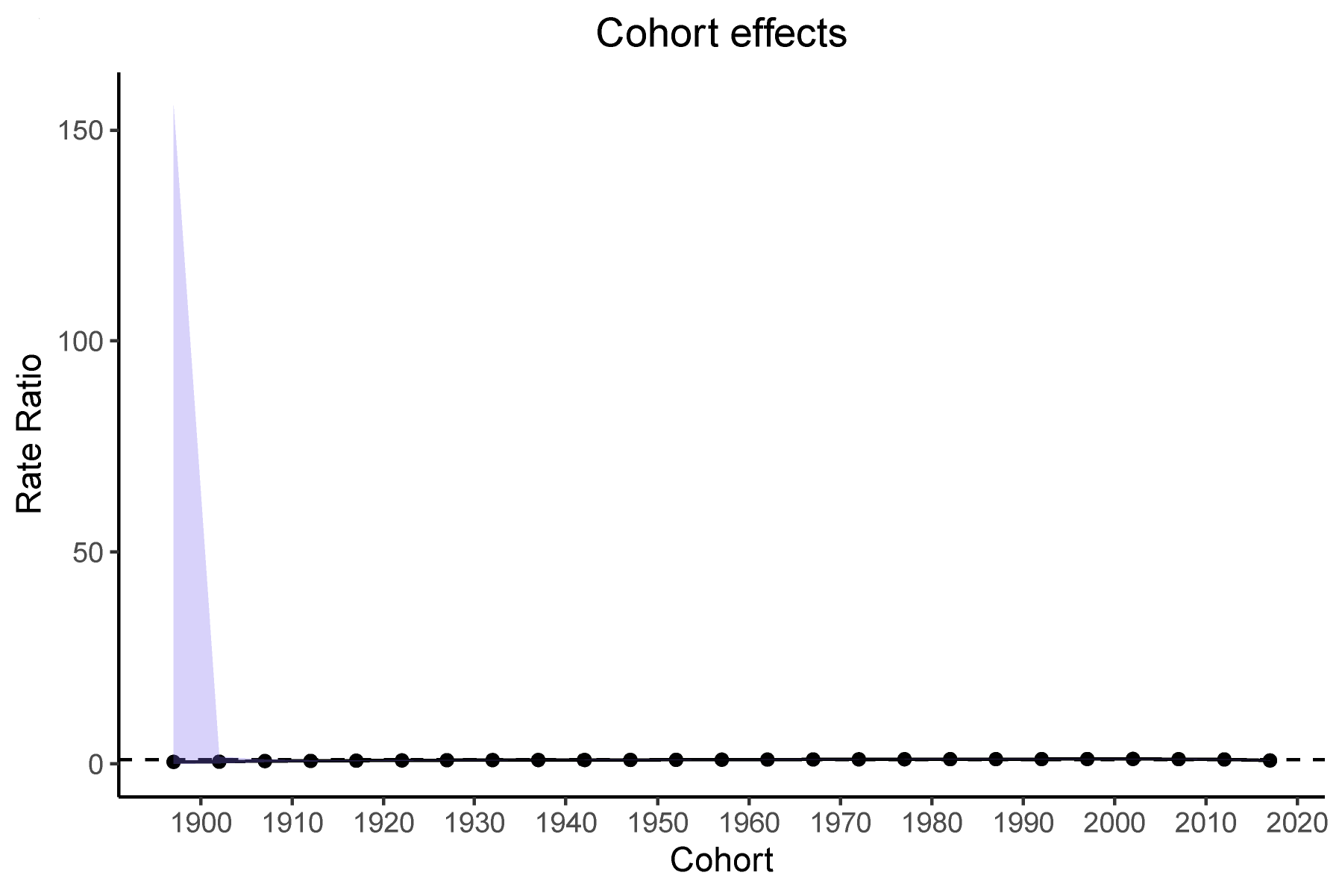

Supplement: supplementary figures and sub supplementary figures.zip [file IRNF_A_2564373_SM4375.zip › supplementary figures and sub supplementary figures/sub supplementary figures/supplementary figure8D.tif]

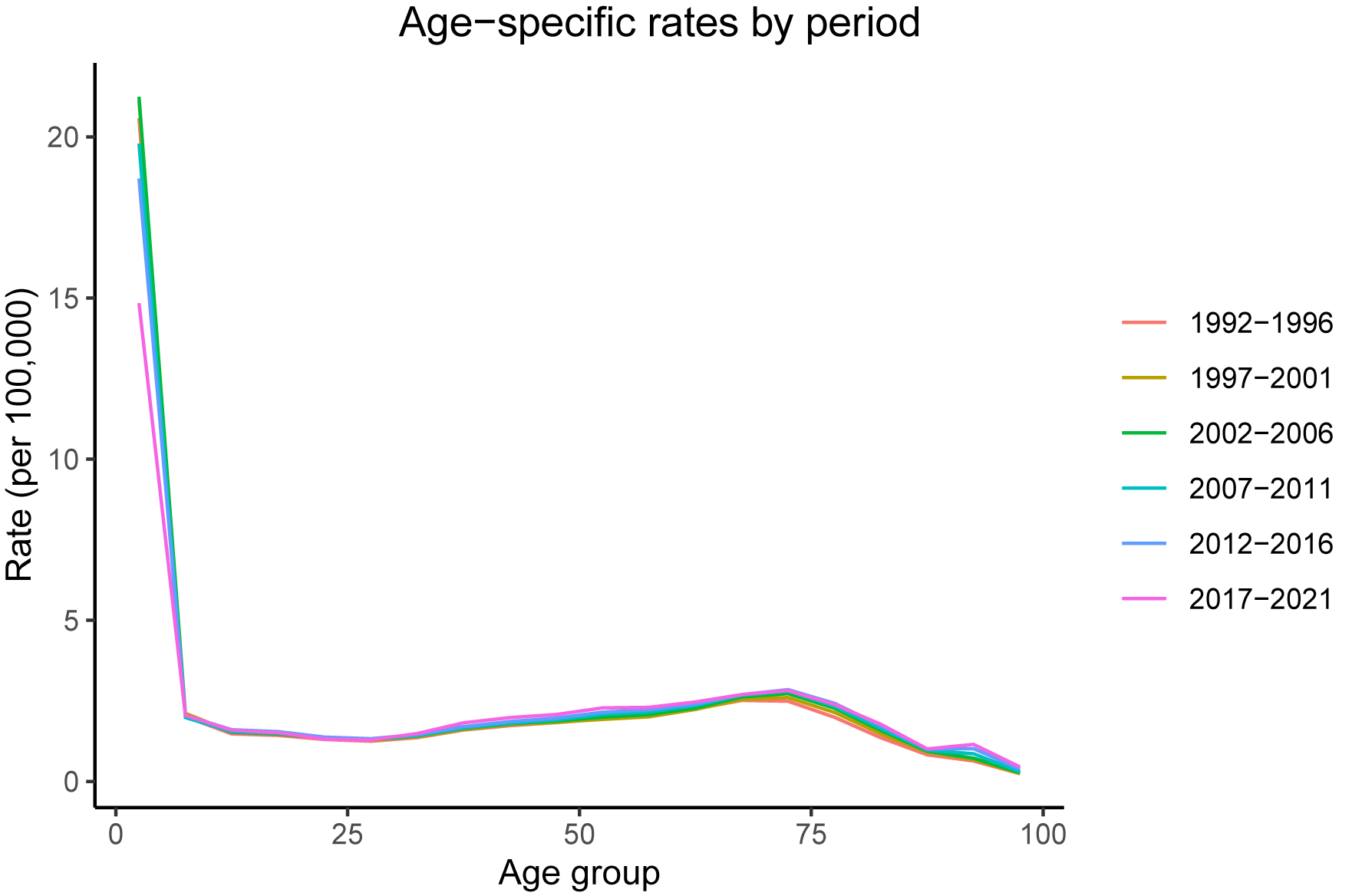

Supplement: supplementary figures and sub supplementary figures.zip [file IRNF_A_2564373_SM4375.zip › supplementary figures and sub supplementary figures/sub supplementary figures/supplementary figure8E.tif]

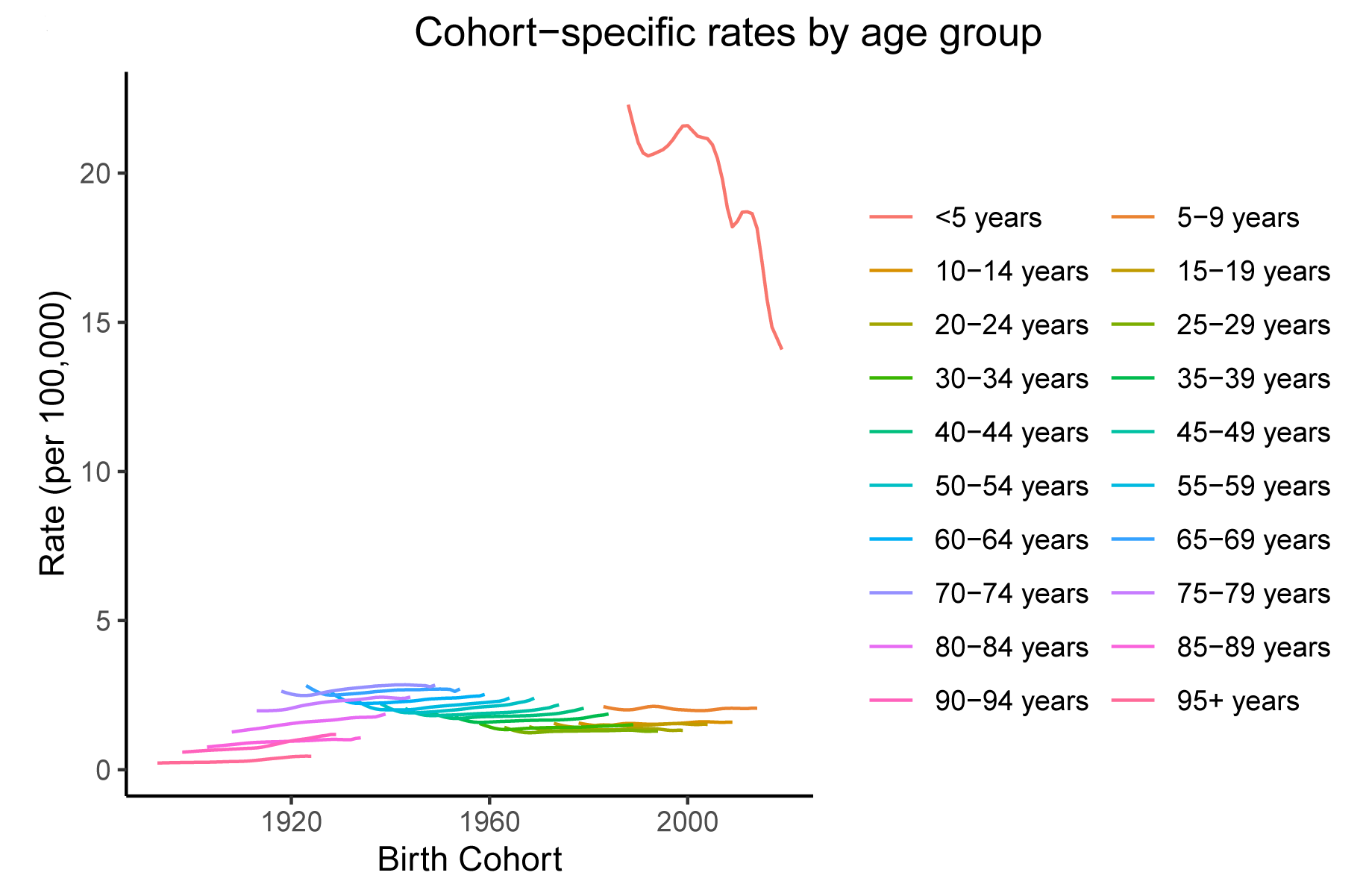

Supplement: supplementary figures and sub supplementary figures.zip [file IRNF_A_2564373_SM4375.zip › supplementary figures and sub supplementary figures/sub supplementary figures/supplementary figure8F.tif]

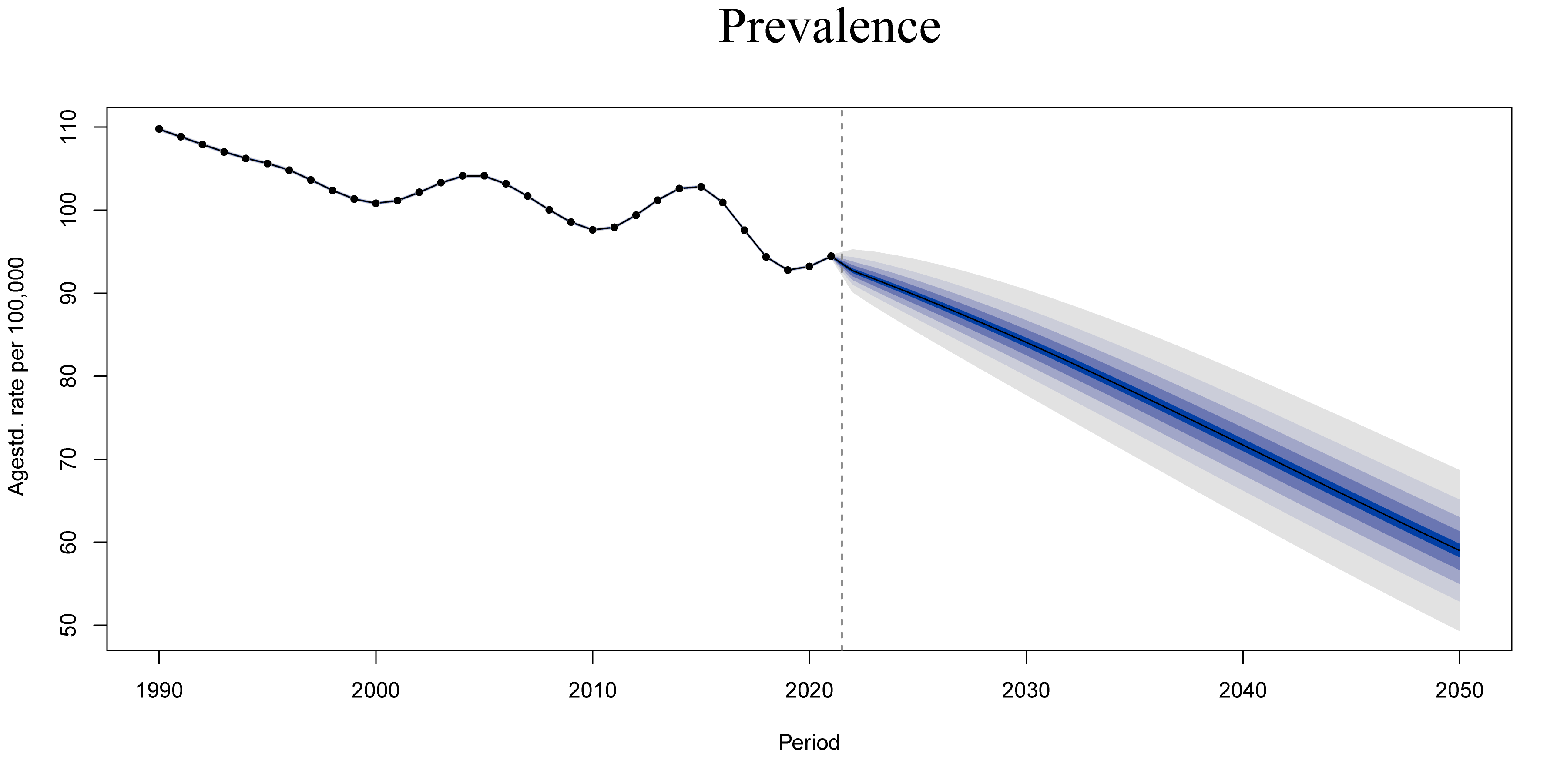

Supplement: supplementary figures and sub supplementary figures.zip [file IRNF_A_2564373_SM4375.zip › supplementary figures and sub supplementary figures/sub supplementary figures/supplementary figure9A.tif]

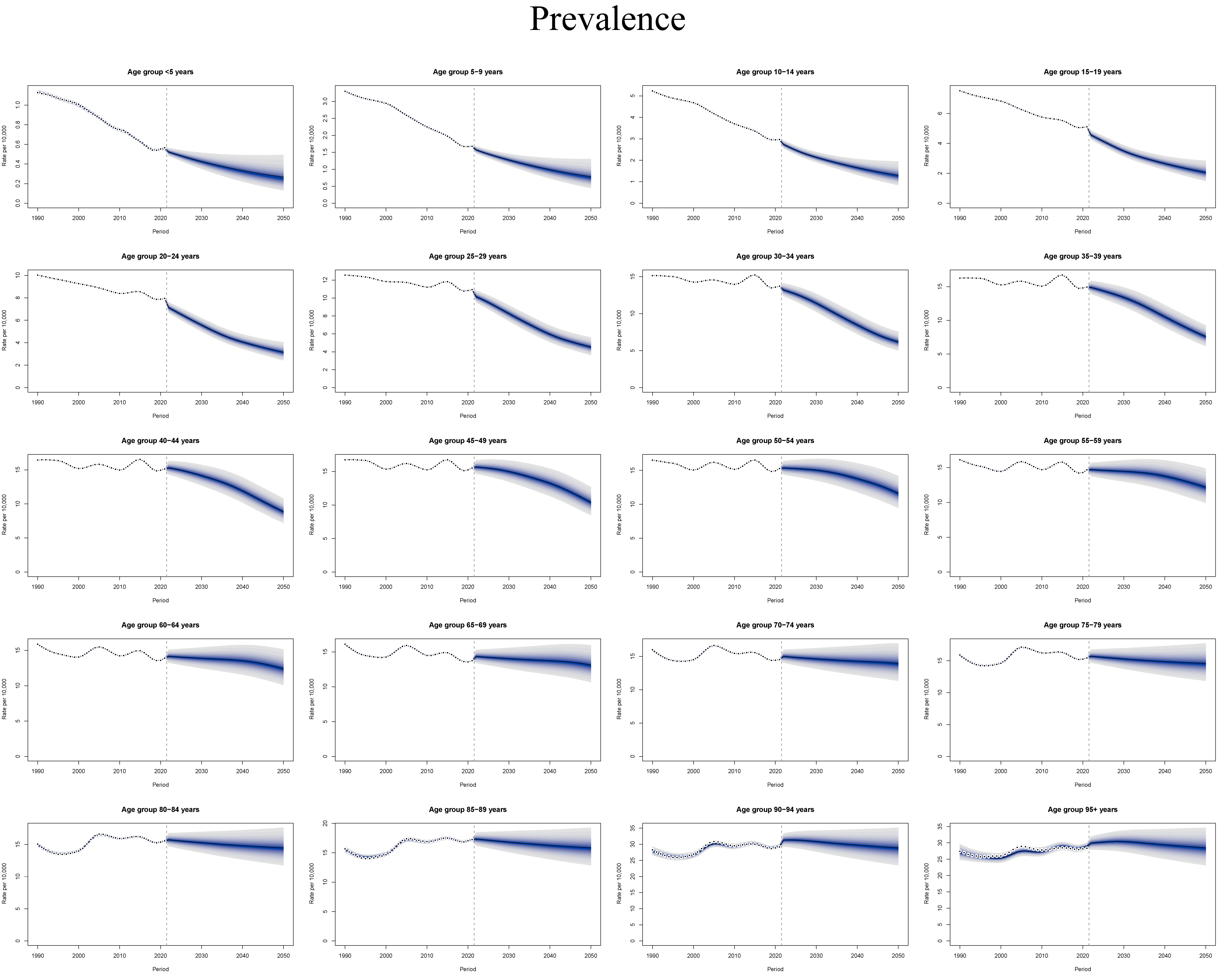

Supplement: supplementary figures and sub supplementary figures.zip [file IRNF_A_2564373_SM4375.zip › supplementary figures and sub supplementary figures/sub supplementary figures/supplementary figure9B.tif]

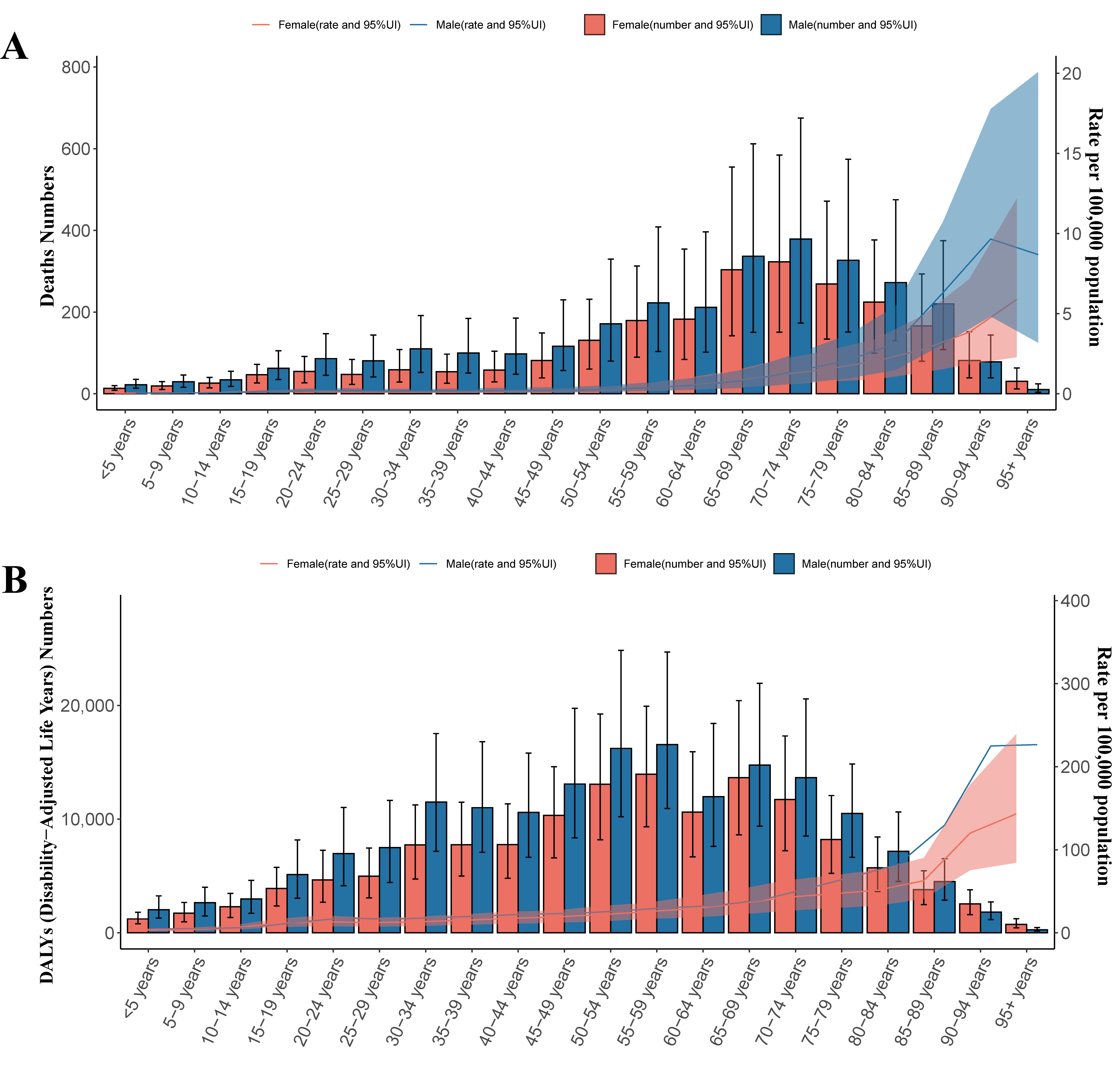

Supplement: supplementary figures and sub supplementary figures.zip [file IRNF_A_2564373_SM4375.zip › supplementary figures and sub supplementary figures/supplementary figures/supplementary figure1.tif]

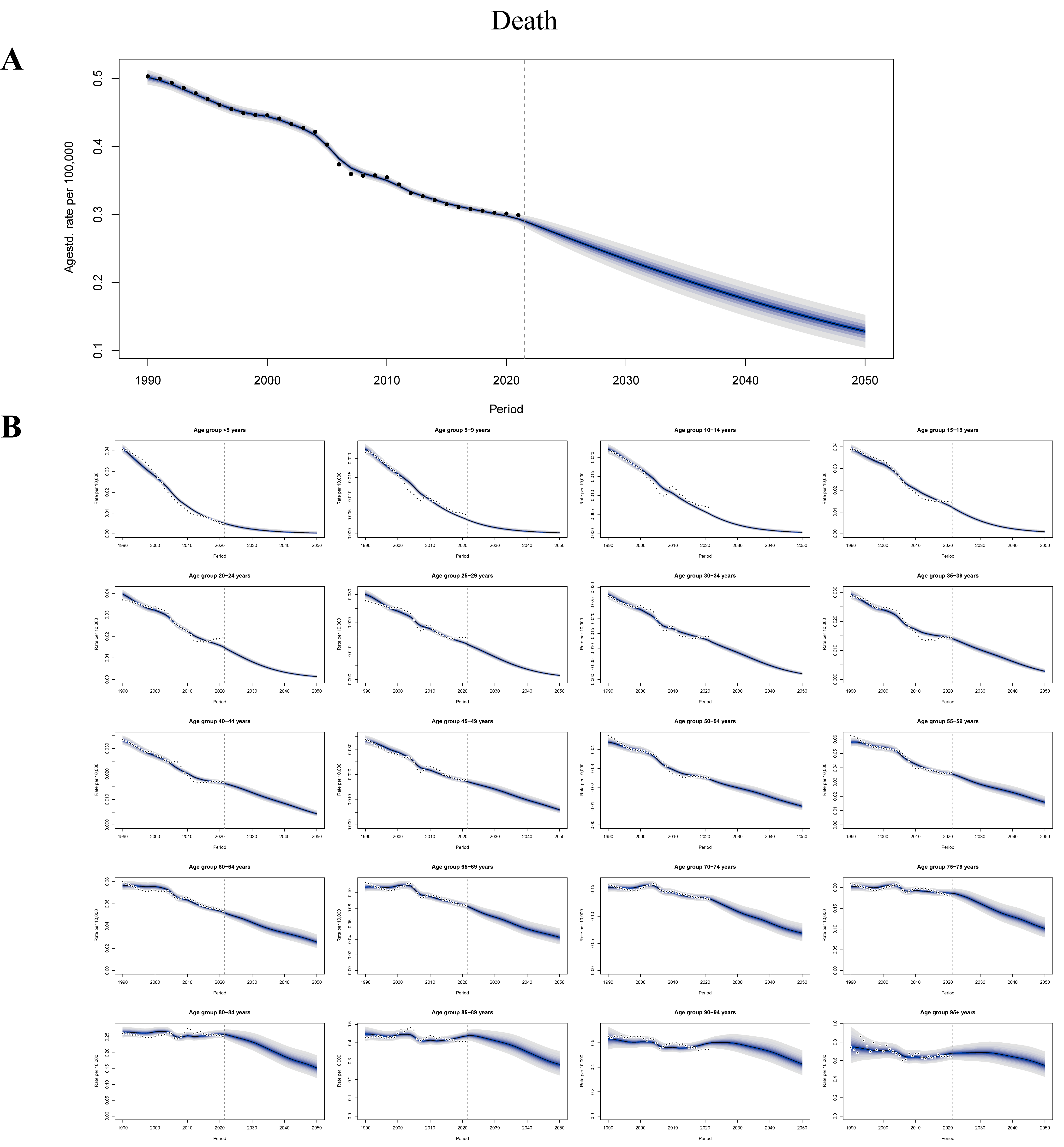

Supplement: supplementary figures and sub supplementary figures.zip [file IRNF_A_2564373_SM4375.zip › supplementary figures and sub supplementary figures/supplementary figures/supplementary figure11.tif]

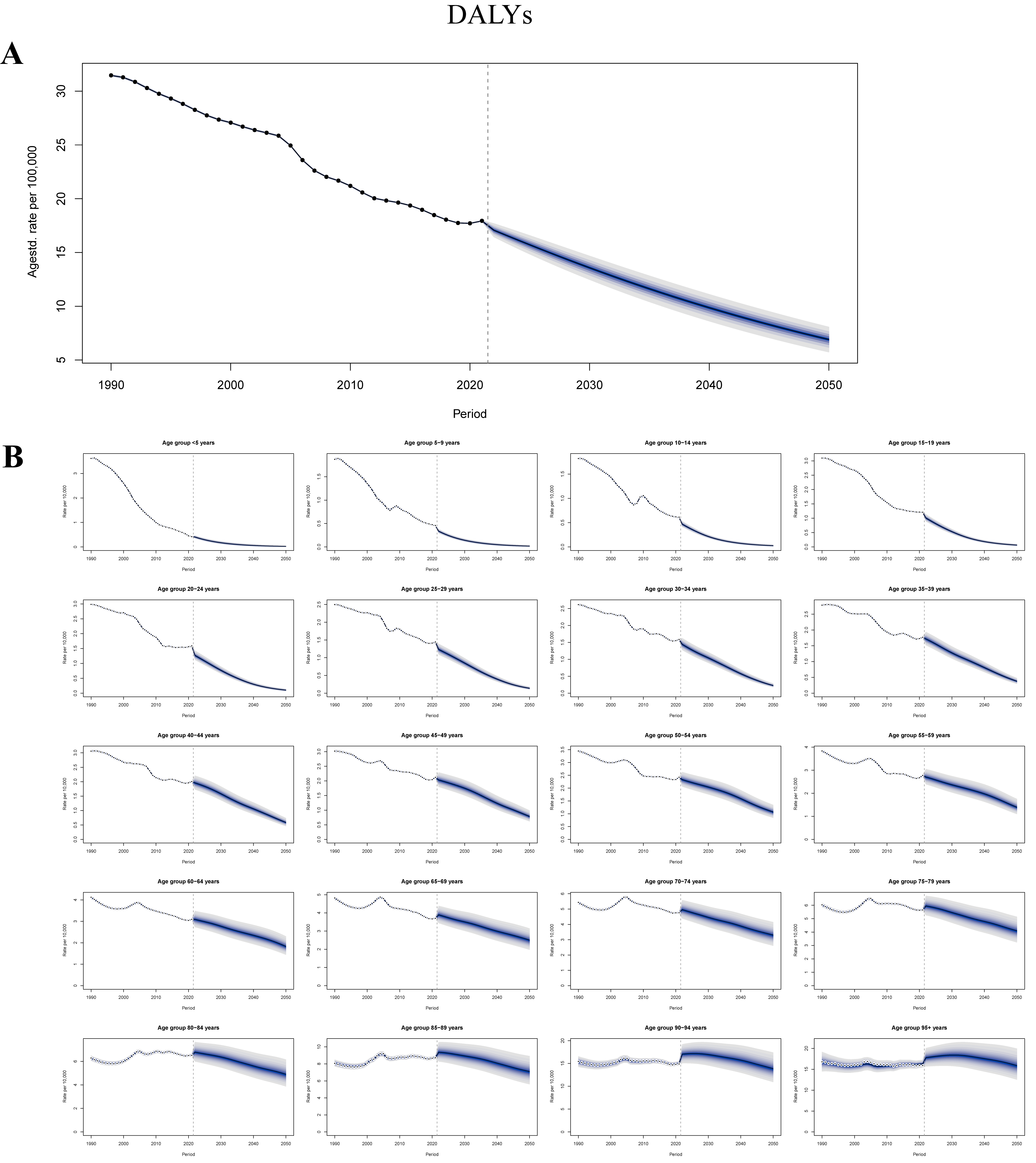

Supplement: supplementary figures and sub supplementary figures.zip [file IRNF_A_2564373_SM4375.zip › supplementary figures and sub supplementary figures/supplementary figures/supplementary figure12.tif]

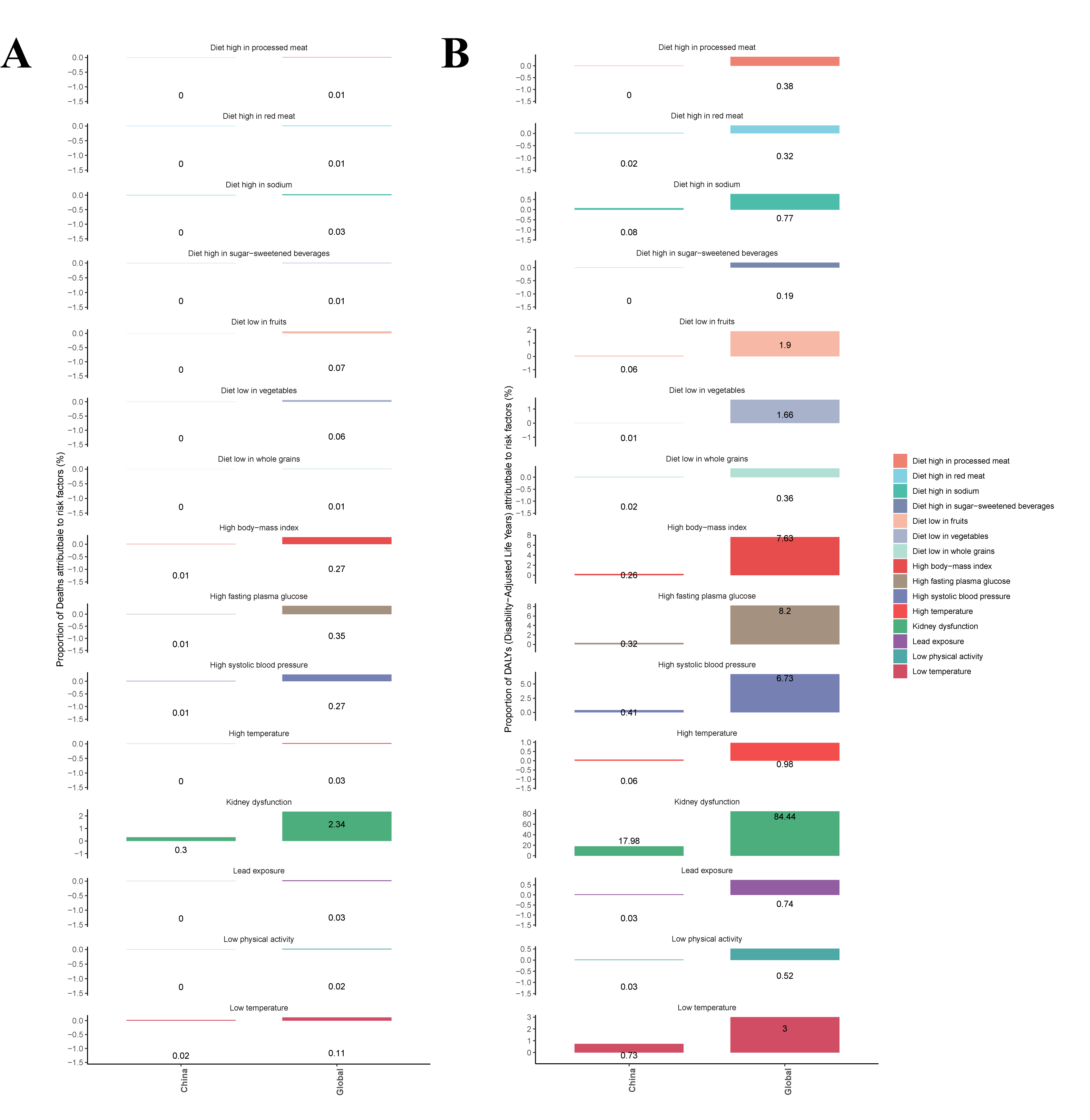

Supplement: supplementary figures and sub supplementary figures.zip [file IRNF_A_2564373_SM4375.zip › supplementary figures and sub supplementary figures/supplementary figures/supplementary figure13.tif]

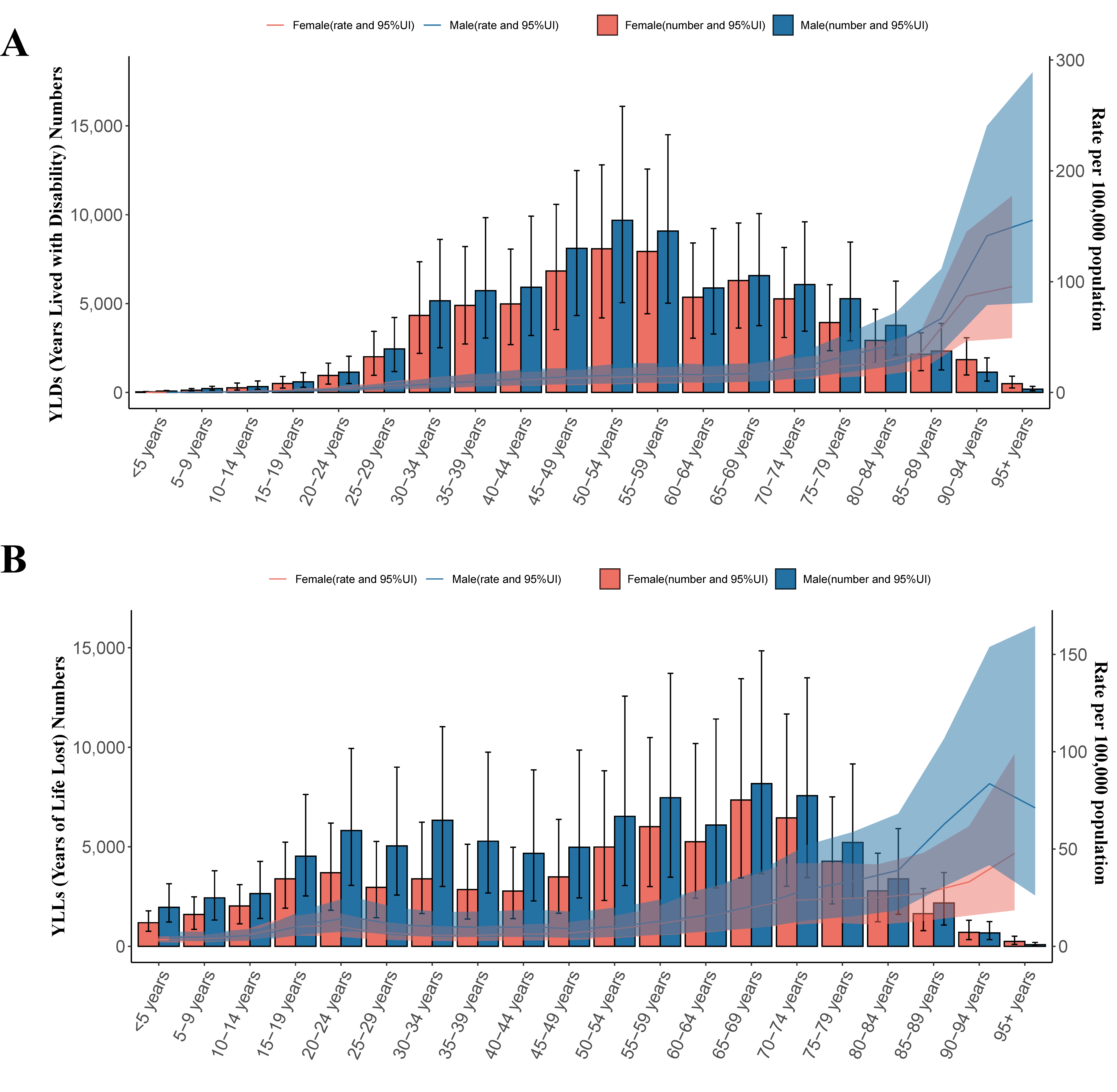

Supplement: supplementary figures and sub supplementary figures.zip [file IRNF_A_2564373_SM4375.zip › supplementary figures and sub supplementary figures/supplementary figures/supplementary figure2.tif]

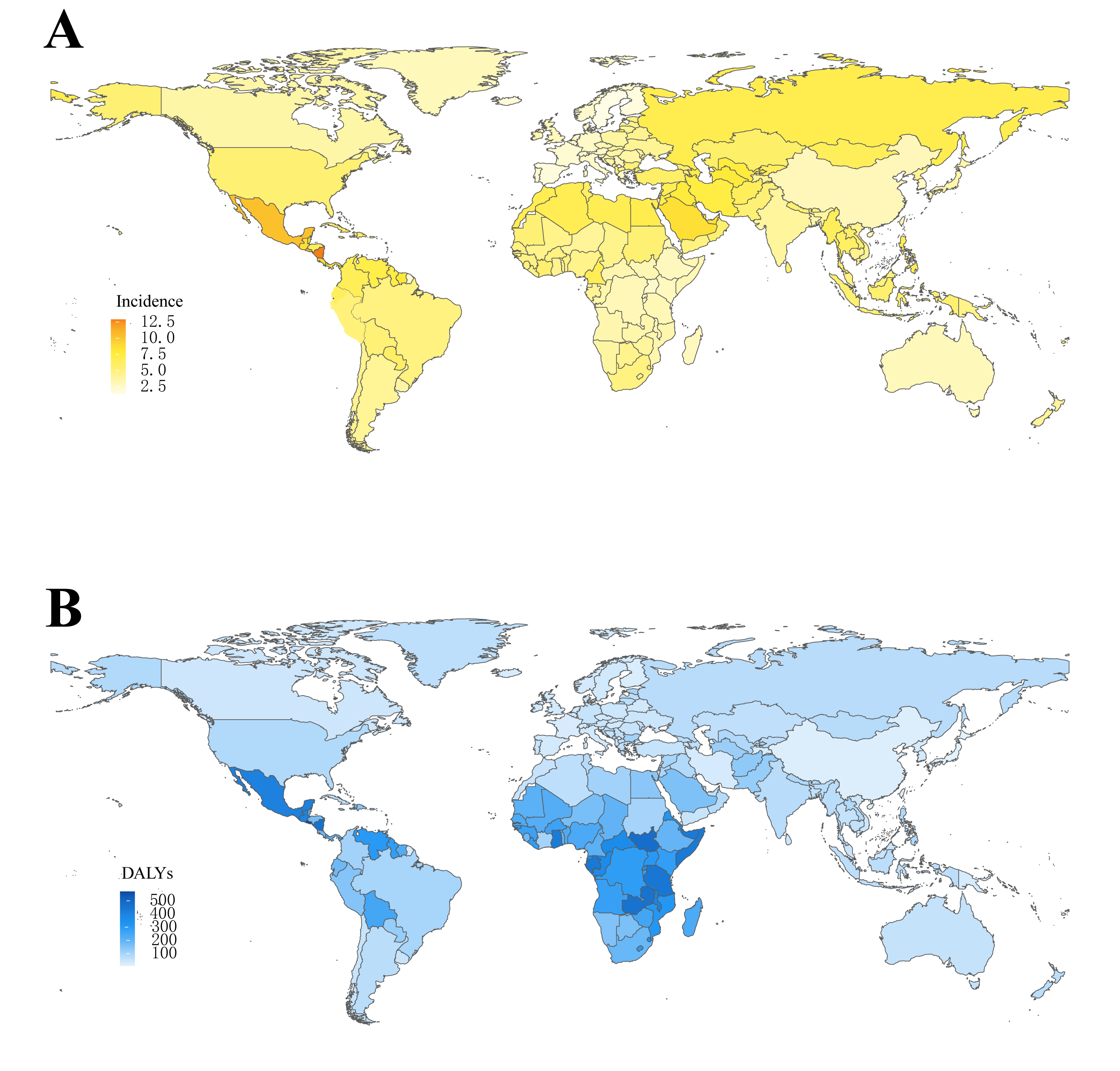

Supplement: supplementary figures and sub supplementary figures.zip [file IRNF_A_2564373_SM4375.zip › supplementary figures and sub supplementary figures/supplementary figures/supplementary figure3.tif]

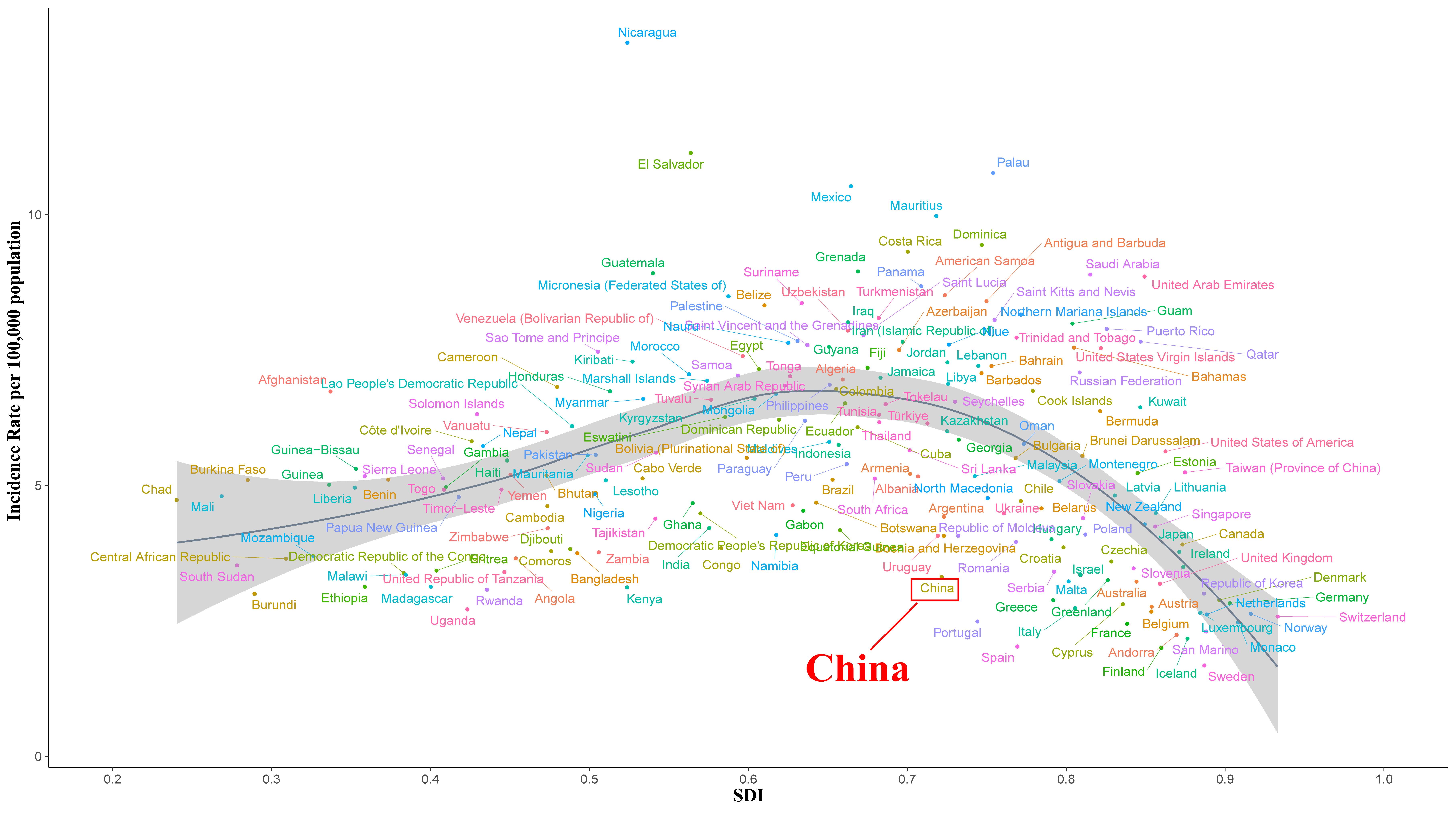

Supplement: supplementary figures and sub supplementary figures.zip [file IRNF_A_2564373_SM4375.zip › supplementary figures and sub supplementary figures/supplementary figures/supplementary figure4.tif]

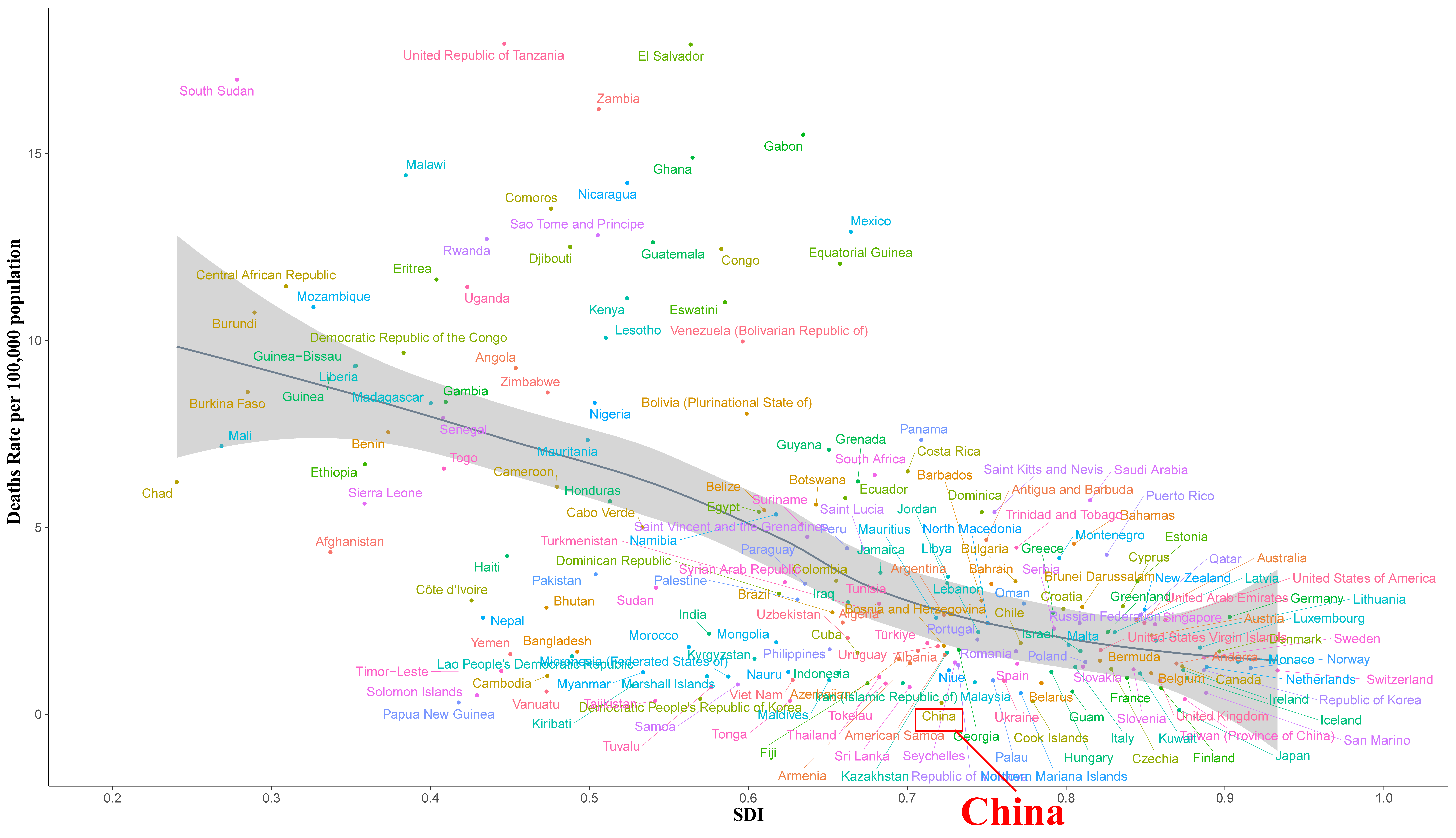

Supplement: supplementary figures and sub supplementary figures.zip [file IRNF_A_2564373_SM4375.zip › supplementary figures and sub supplementary figures/supplementary figures/supplementary figure5.tif]

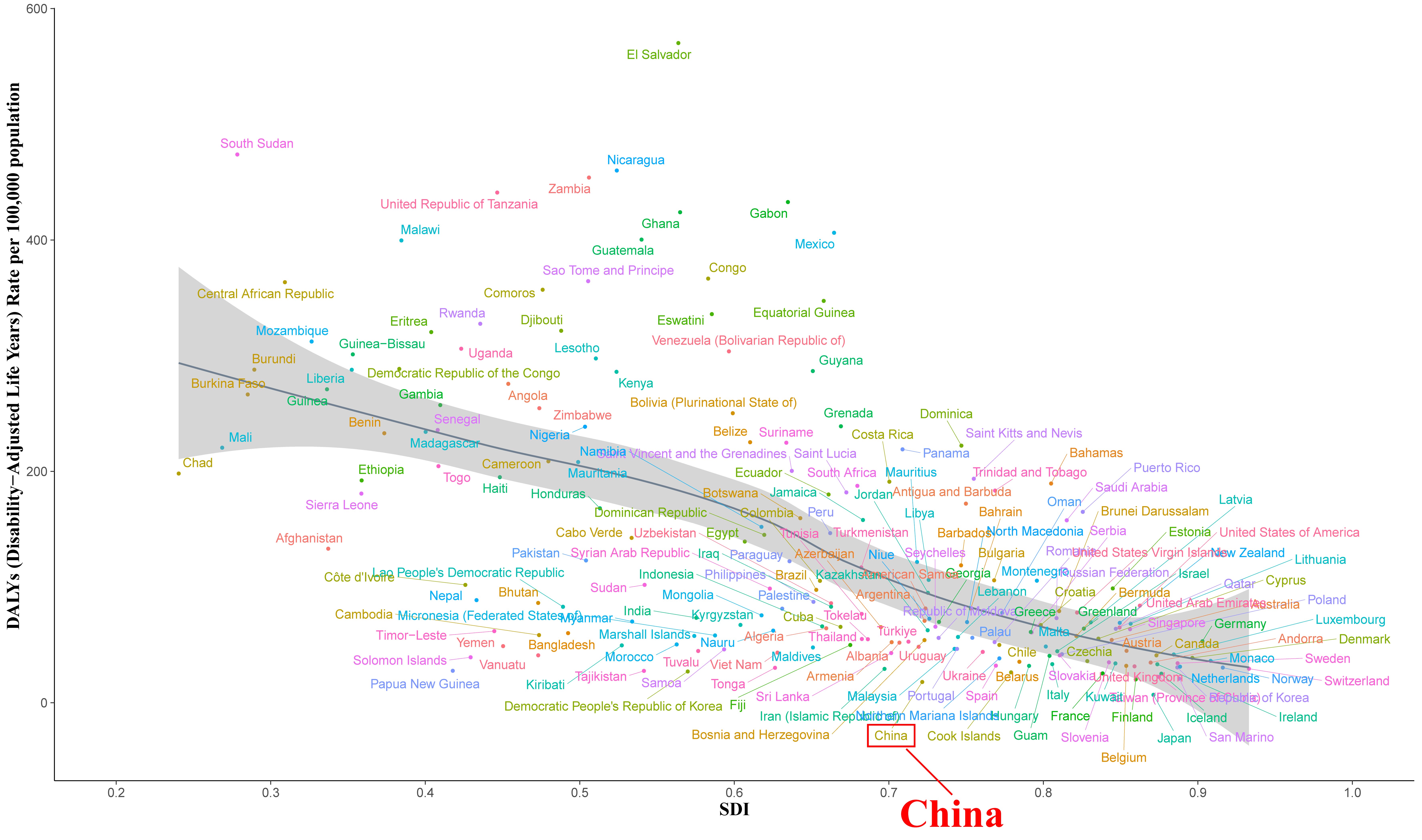

Supplement: supplementary figures and sub supplementary figures.zip [file IRNF_A_2564373_SM4375.zip › supplementary figures and sub supplementary figures/supplementary figures/supplementary figure6.tif]

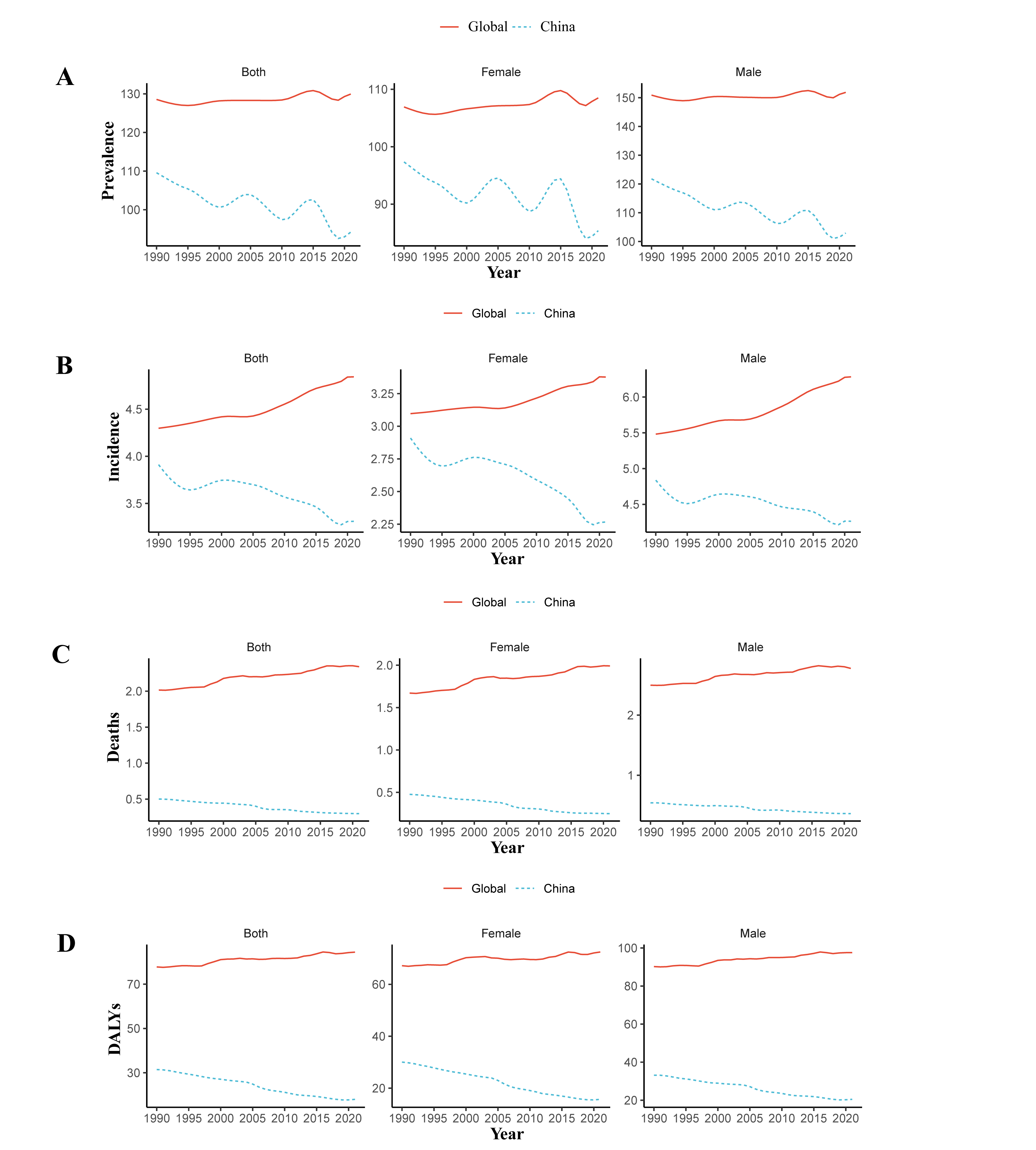

Supplement: supplementary figures and sub supplementary figures.zip [file IRNF_A_2564373_SM4375.zip › supplementary figures and sub supplementary figures/supplementary figures/supplementary figure7.tif]

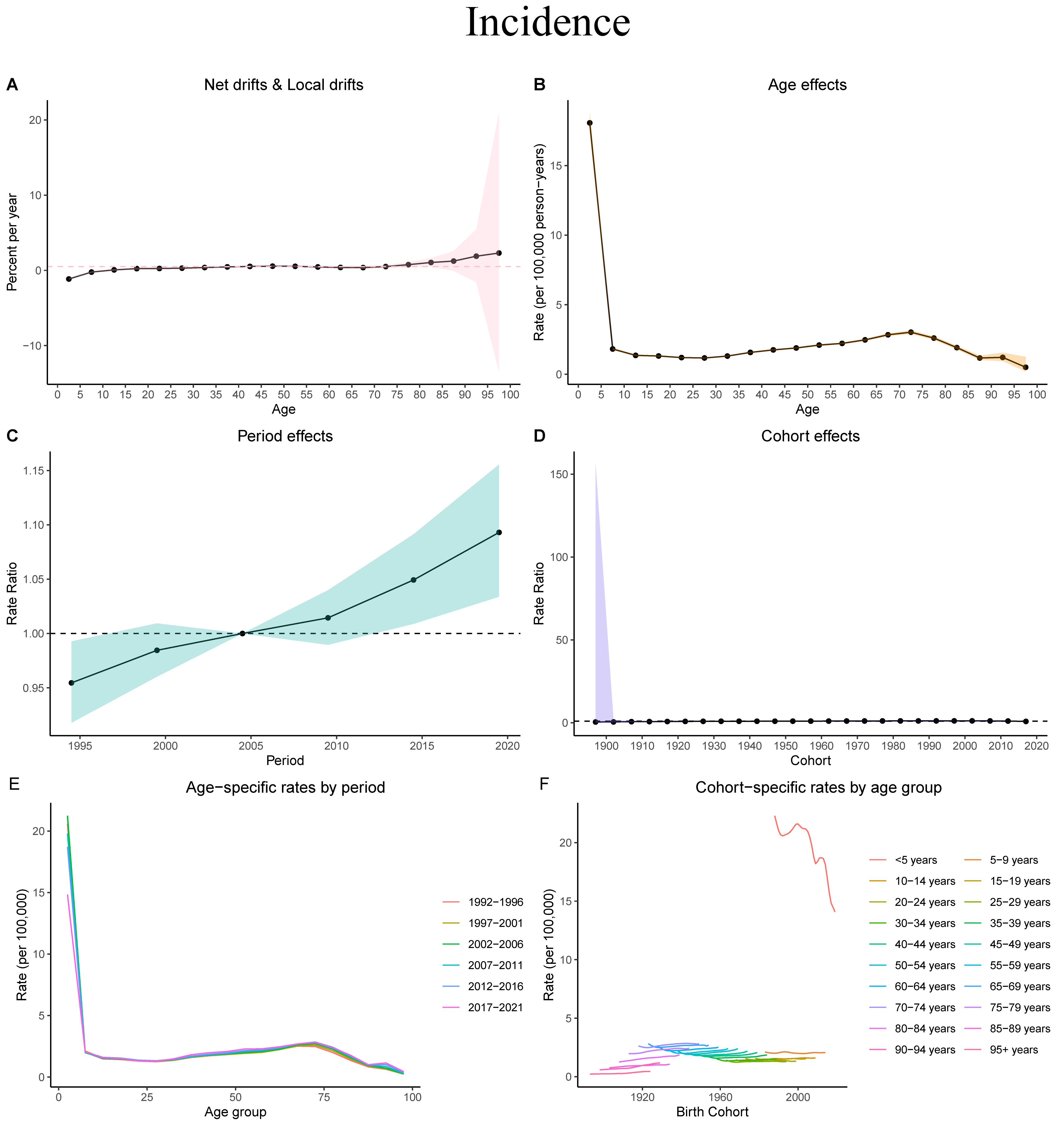

Supplement: supplementary figures and sub supplementary figures.zip [file IRNF_A_2564373_SM4375.zip › supplementary figures and sub supplementary figures/supplementary figures/supplementary figure8.tif]

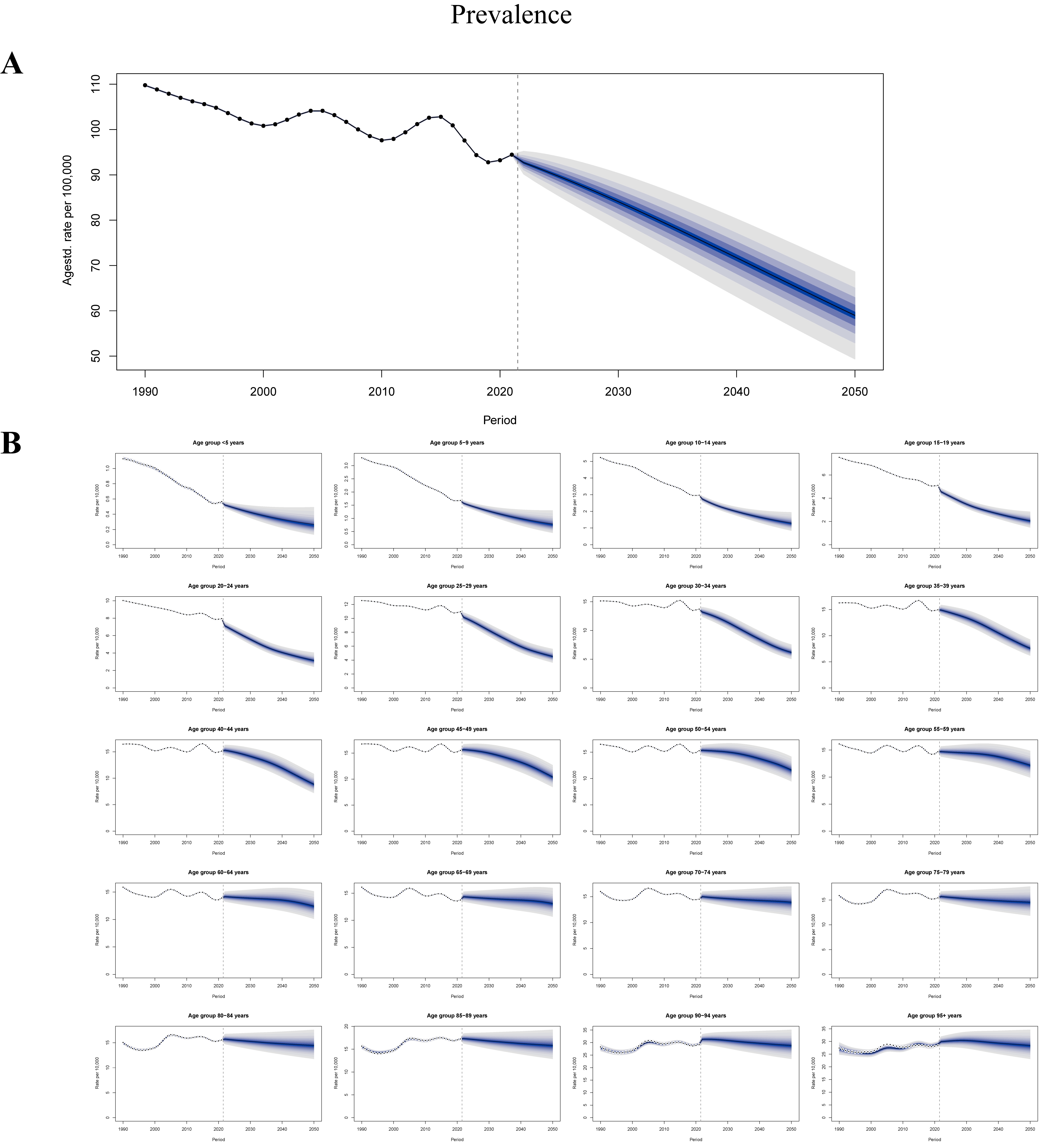

Supplement: supplementary figures and sub supplementary figures.zip [file IRNF_A_2564373_SM4375.zip › supplementary figures and sub supplementary figures/supplementary figures/supplementary figure9.tif]

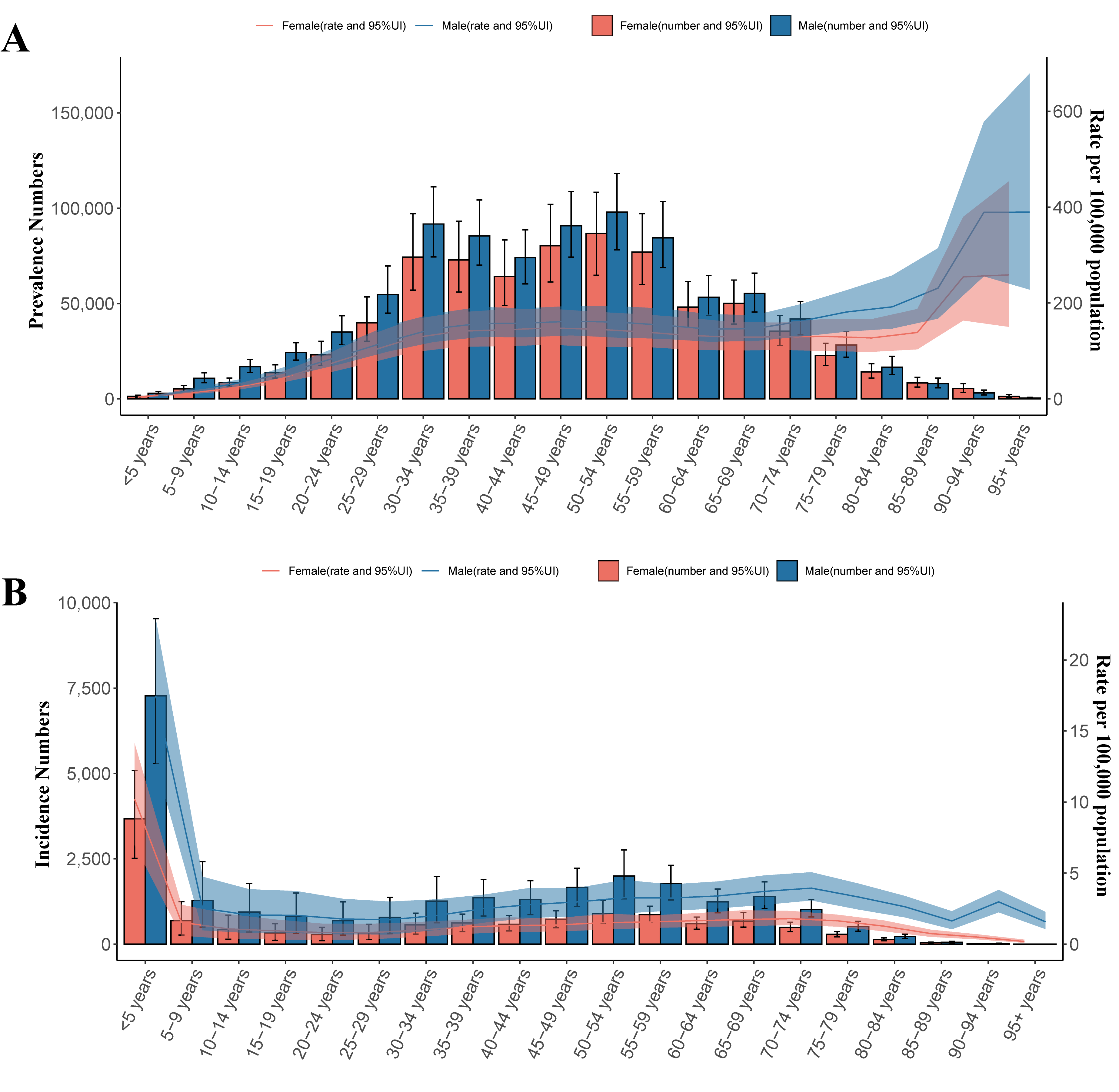

Supplement: figures and sub figures.zip [file IRNF_A_2564373_SM4374.zip › figures and sub figures/figures/figure1.tif]

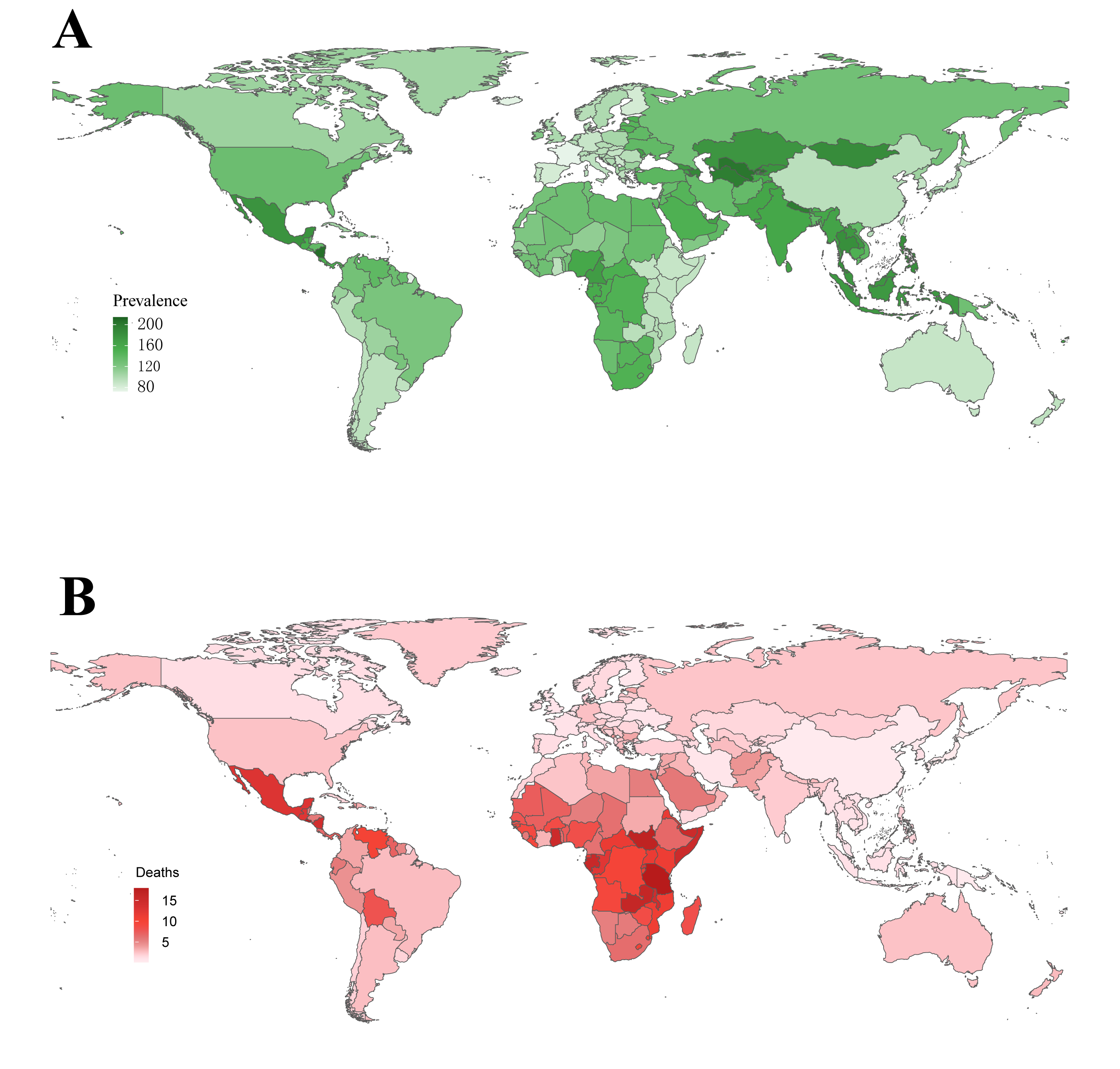

Supplement: figures and sub figures.zip [file IRNF_A_2564373_SM4374.zip › figures and sub figures/figures/figure2.tif]

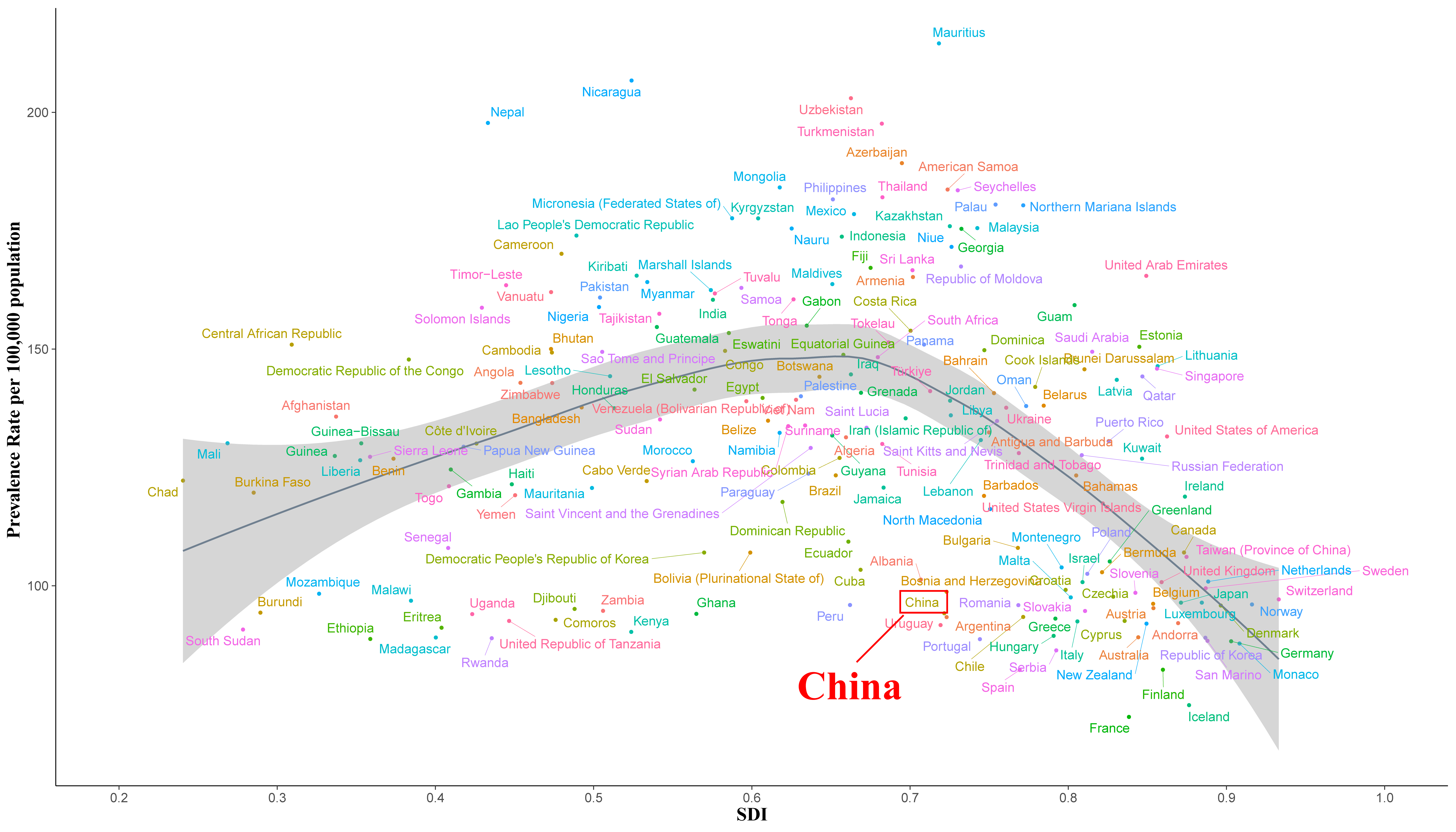

Supplement: figures and sub figures.zip [file IRNF_A_2564373_SM4374.zip › figures and sub figures/figures/figure3.tif]

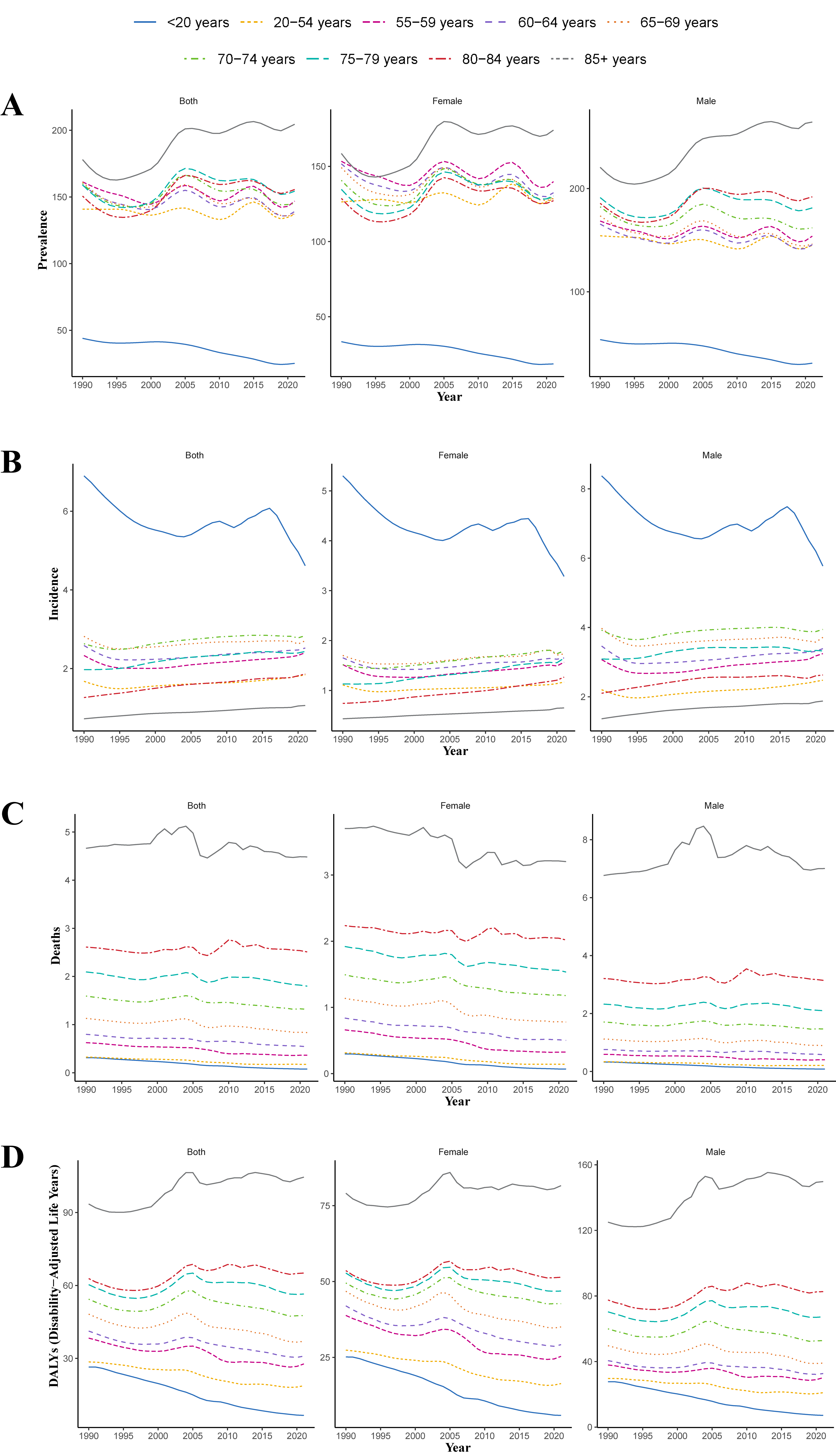

Supplement: figures and sub figures.zip [file IRNF_A_2564373_SM4374.zip › figures and sub figures/figures/figure4.tif]

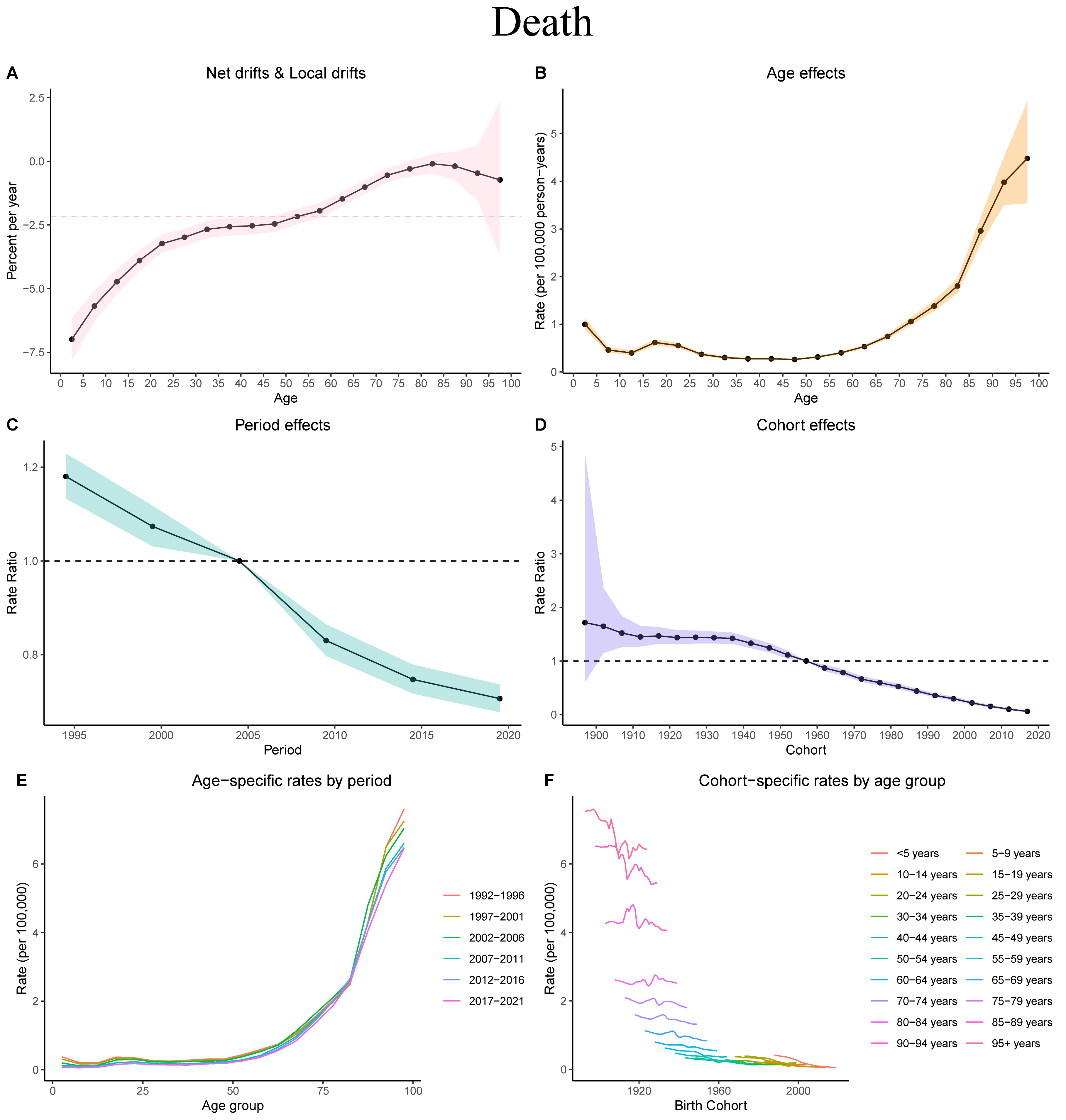

Supplement: figures and sub figures.zip [file IRNF_A_2564373_SM4374.zip › figures and sub figures/figures/figure5.tif]

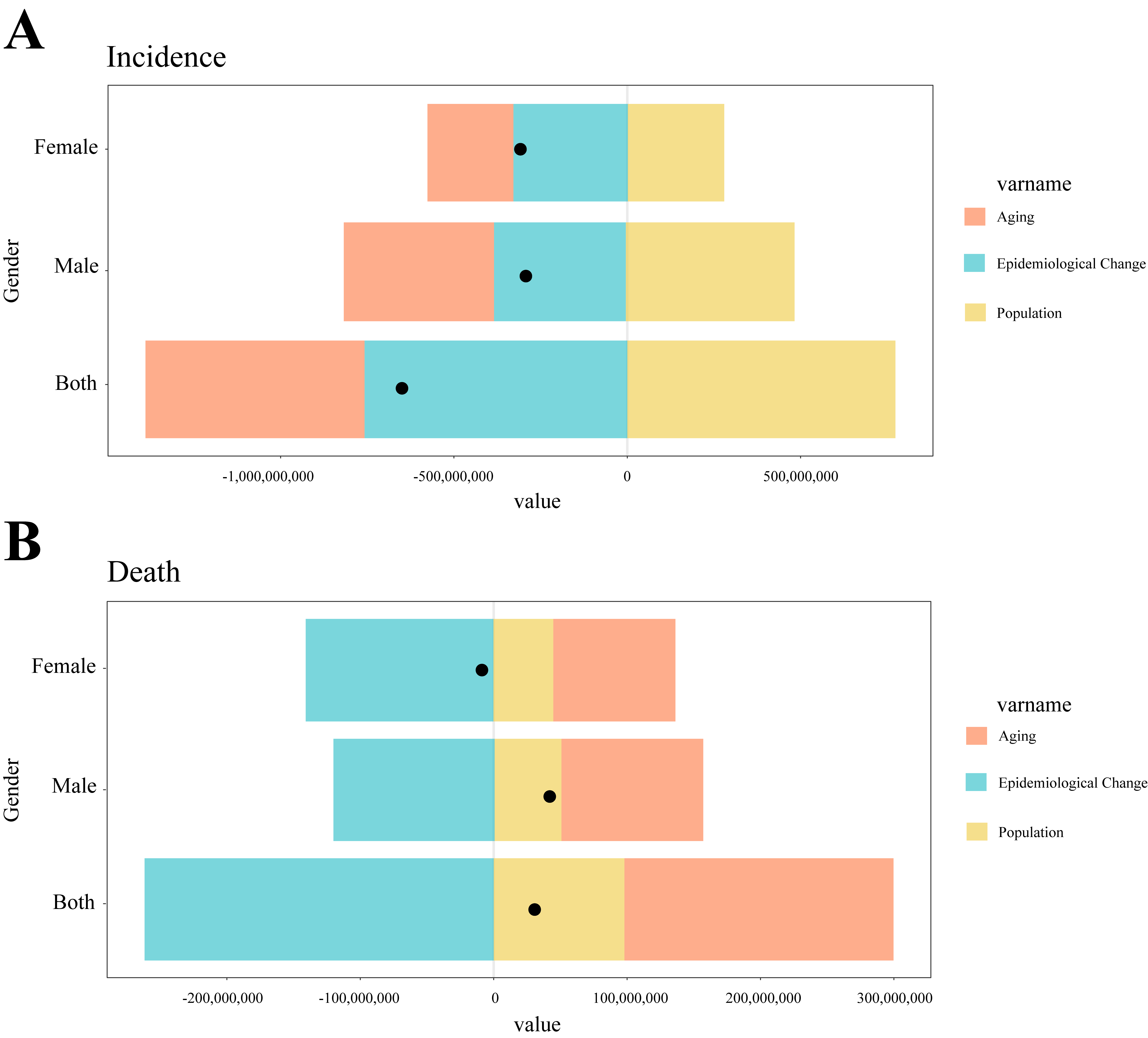

Supplement: figures and sub figures.zip [file IRNF_A_2564373_SM4374.zip › figures and sub figures/figures/figure6.tif]

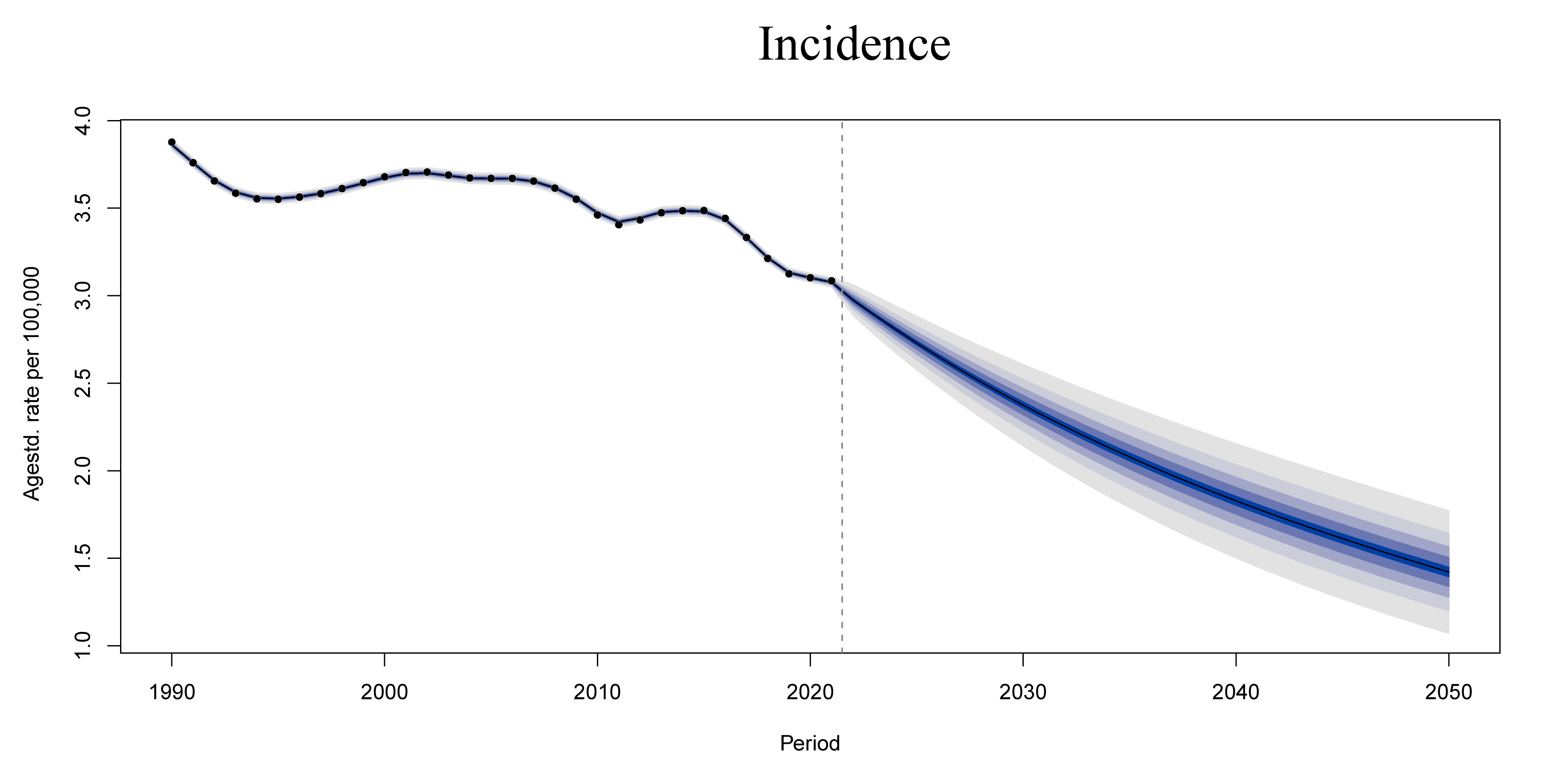

Supplement: figures and sub figures.zip [file IRNF_A_2564373_SM4374.zip › figures and sub figures/figures/figure7.tif]

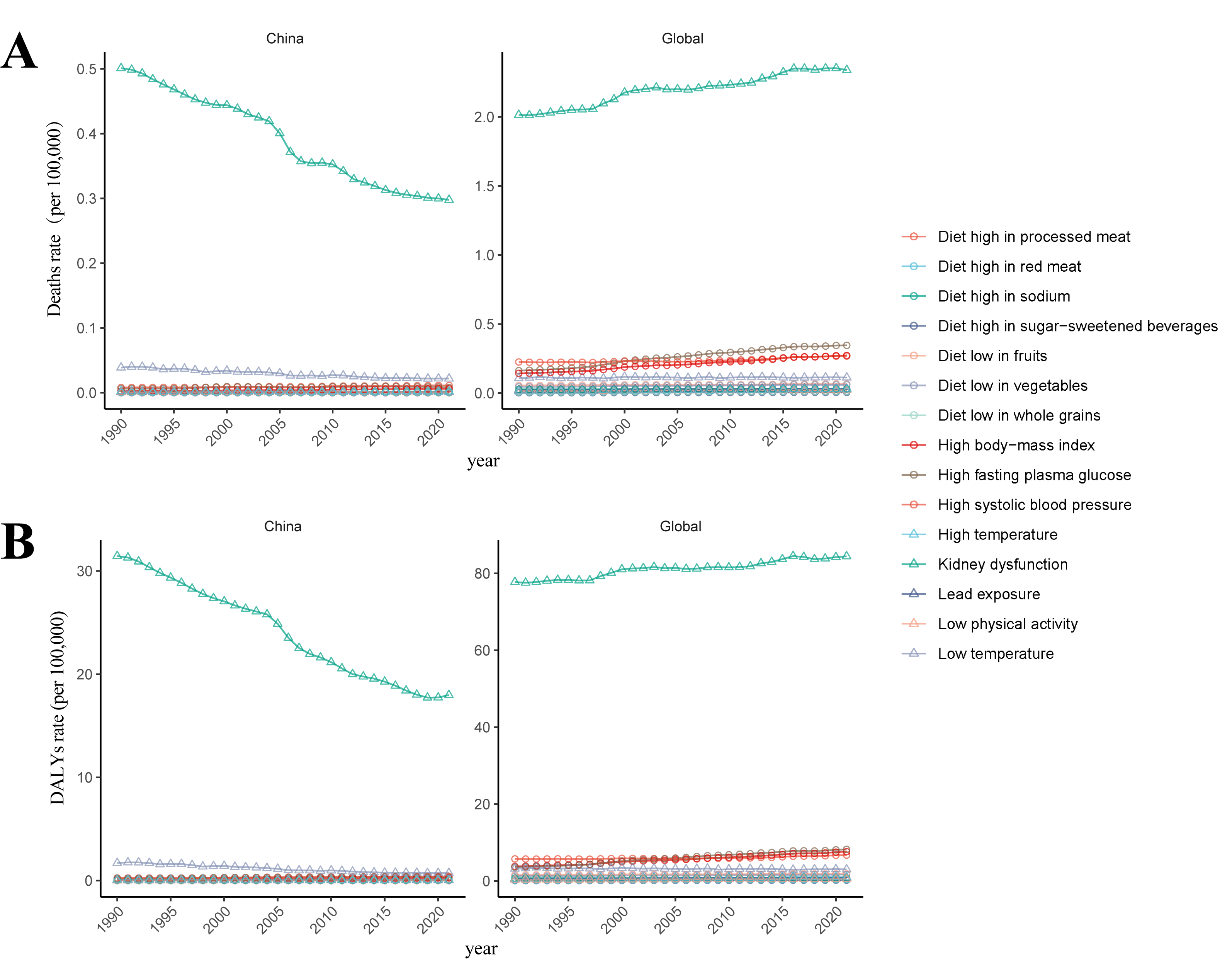

Supplement: figures and sub figures.zip [file IRNF_A_2564373_SM4374.zip › figures and sub figures/figures/figure8.tif]

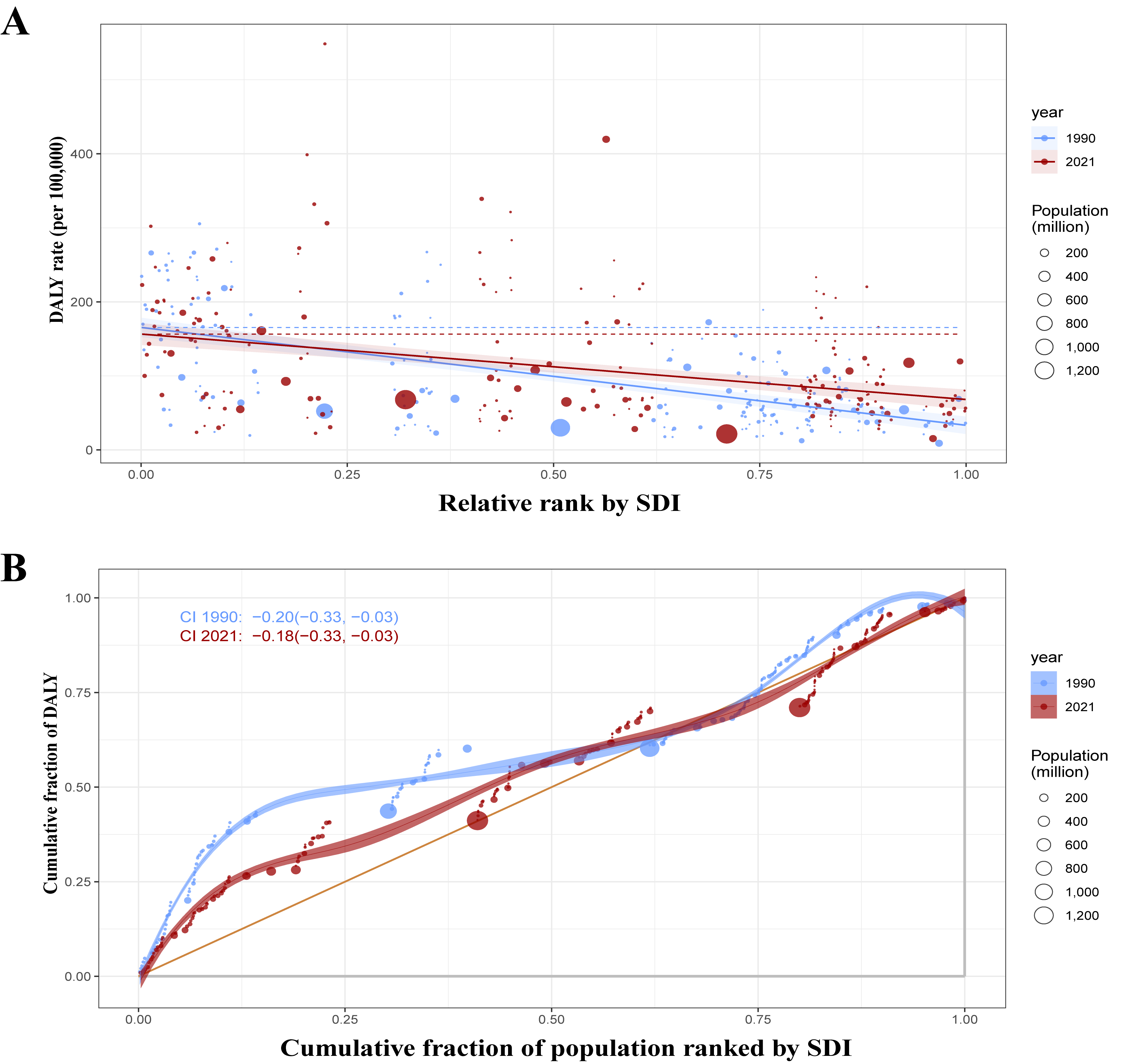

Supplement: figures and sub figures.zip [file IRNF_A_2564373_SM4374.zip › figures and sub figures/figures/figure9.tif]

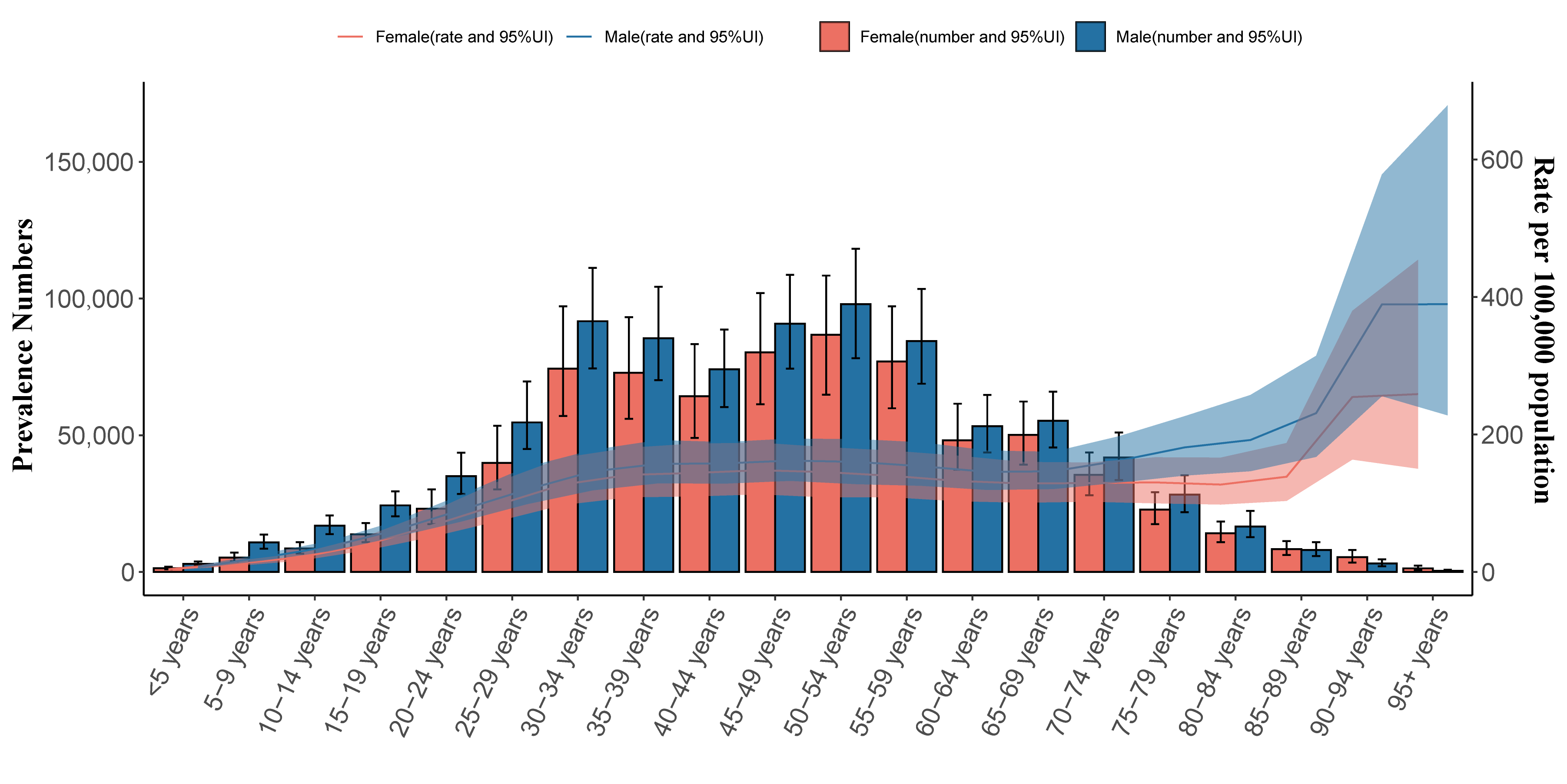

Supplement: figures and sub figures.zip [file IRNF_A_2564373_SM4374.zip › figures and sub figures/sub figures/figure1A.tif]

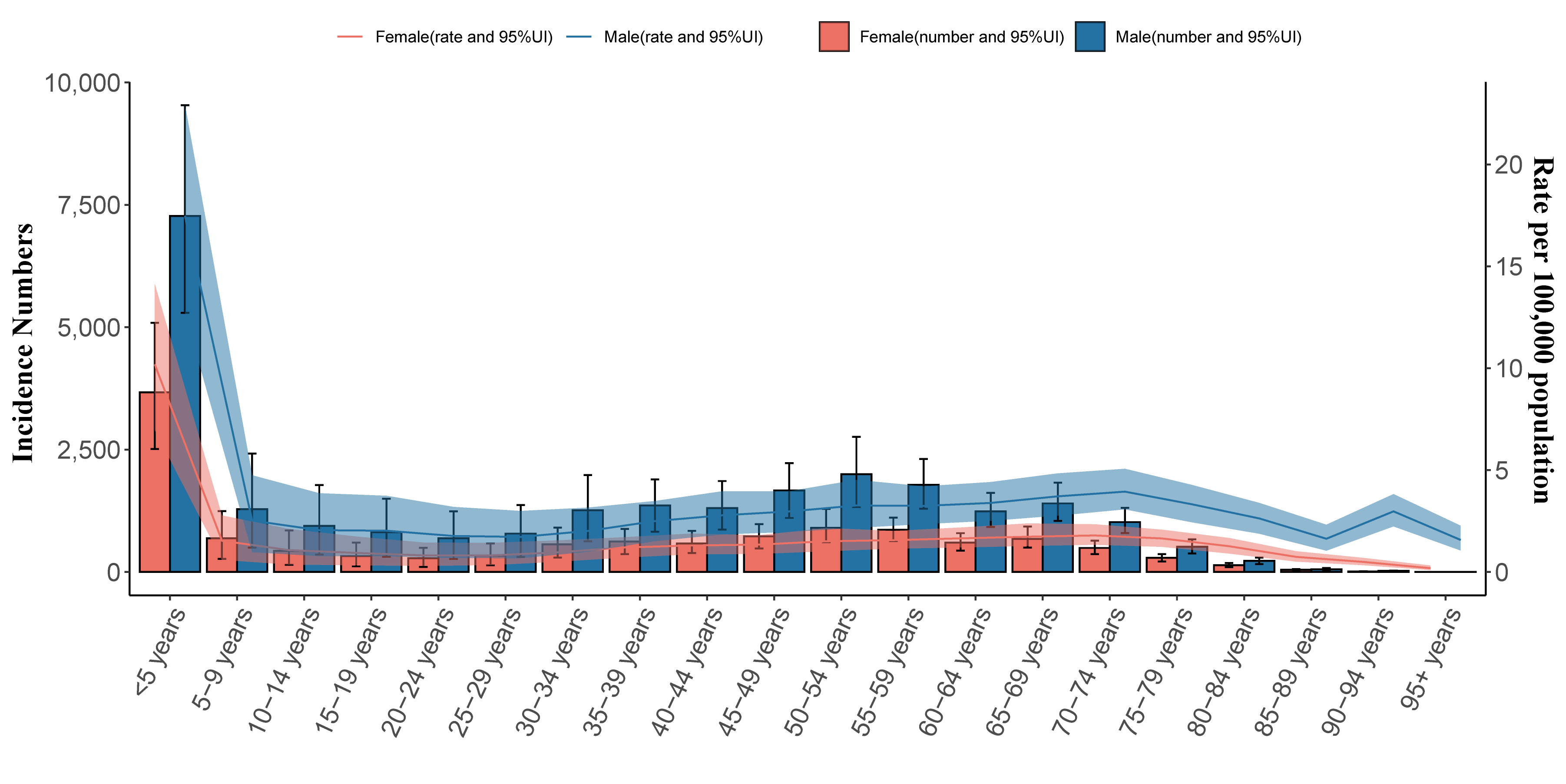

Supplement: figures and sub figures.zip [file IRNF_A_2564373_SM4374.zip › figures and sub figures/sub figures/figure1B.tif]

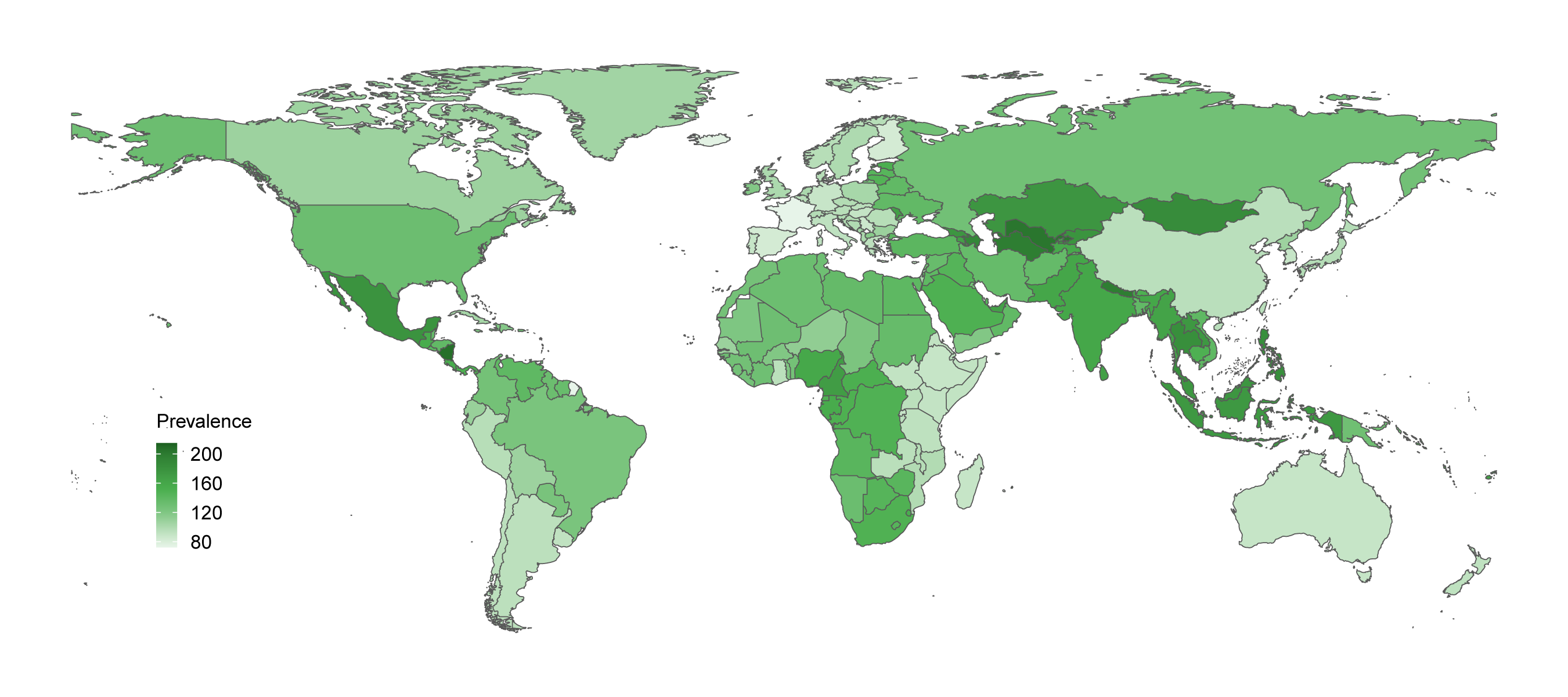

Supplement: figures and sub figures.zip [file IRNF_A_2564373_SM4374.zip › figures and sub figures/sub figures/figure2A.tif]

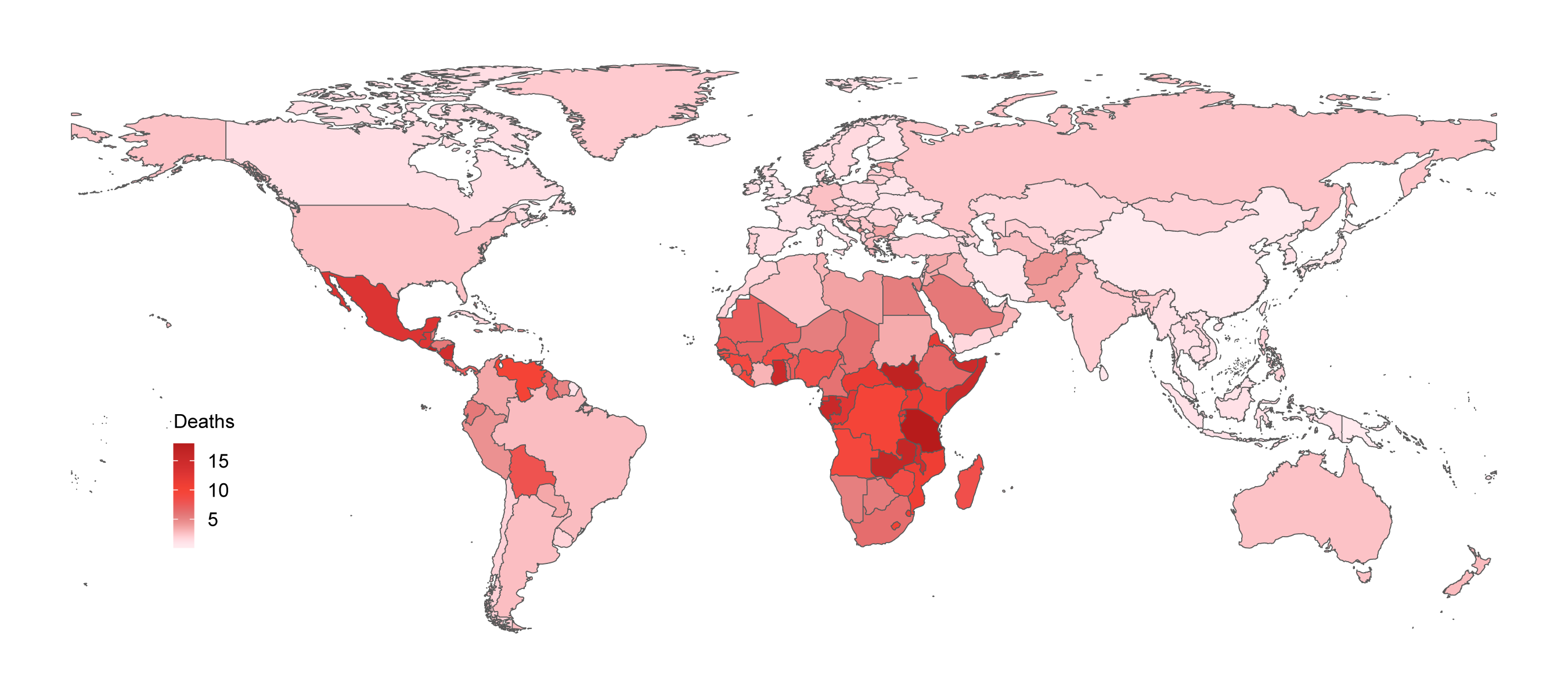

Supplement: figures and sub figures.zip [file IRNF_A_2564373_SM4374.zip › figures and sub figures/sub figures/figure2B.tif]

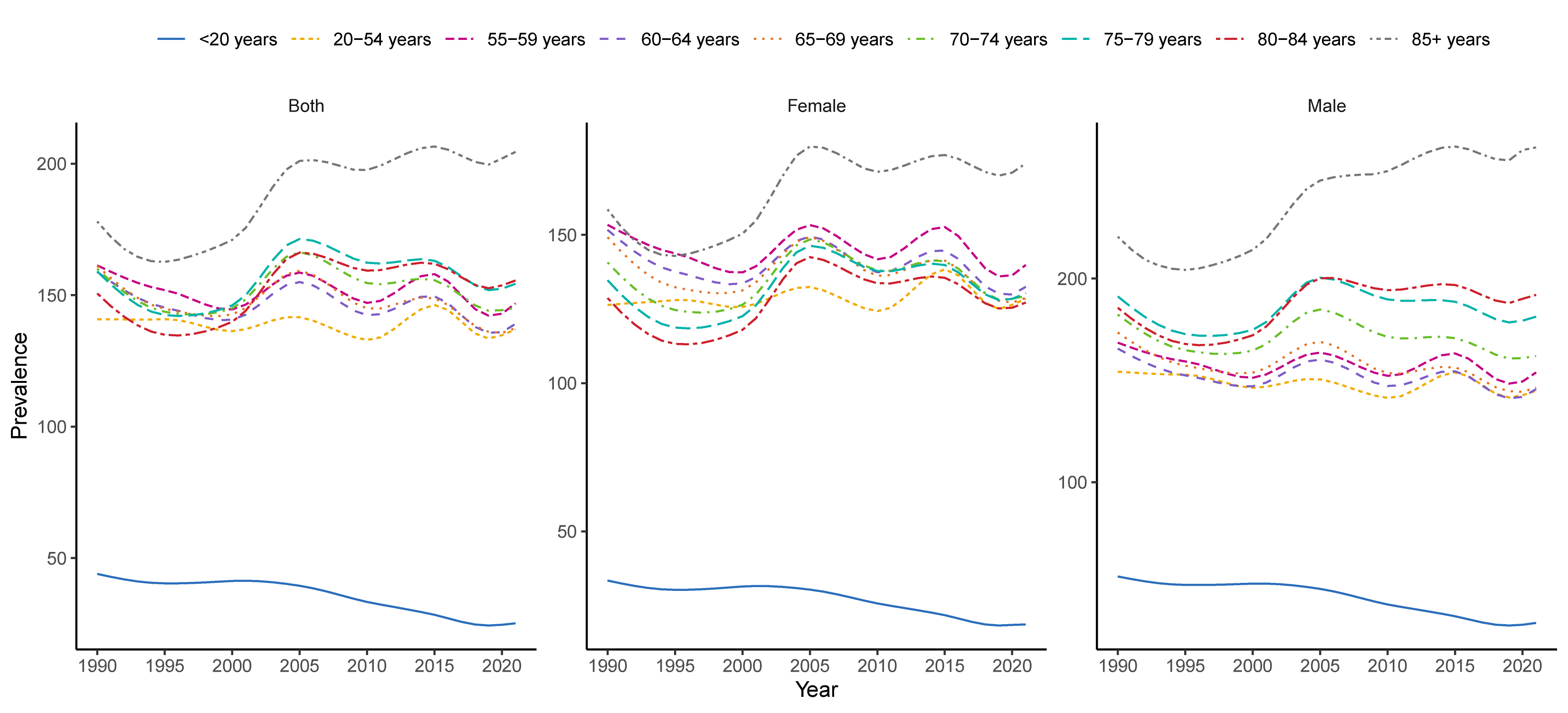

Supplement: figures and sub figures.zip [file IRNF_A_2564373_SM4374.zip › figures and sub figures/sub figures/figure4A.tif]

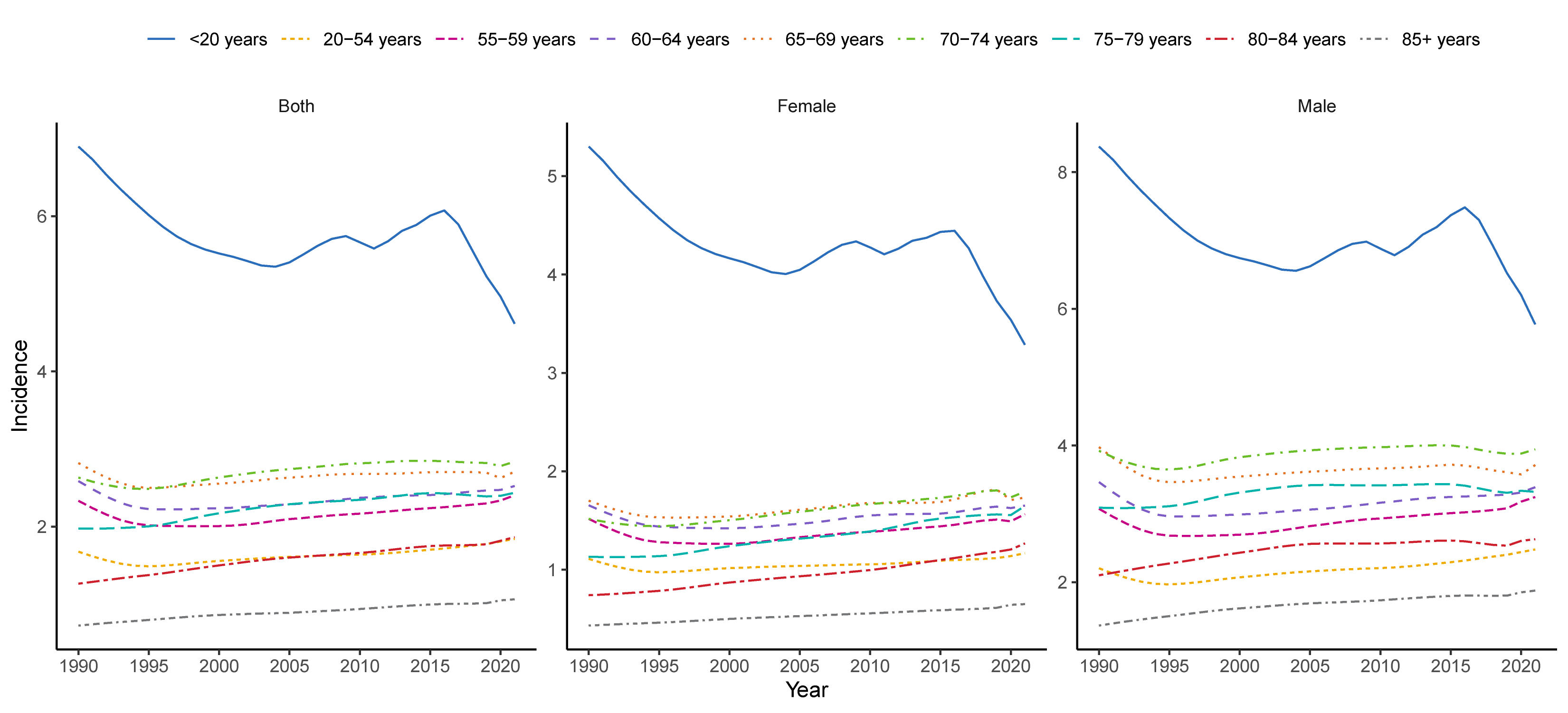

Supplement: figures and sub figures.zip [file IRNF_A_2564373_SM4374.zip › figures and sub figures/sub figures/figure4B.tif]

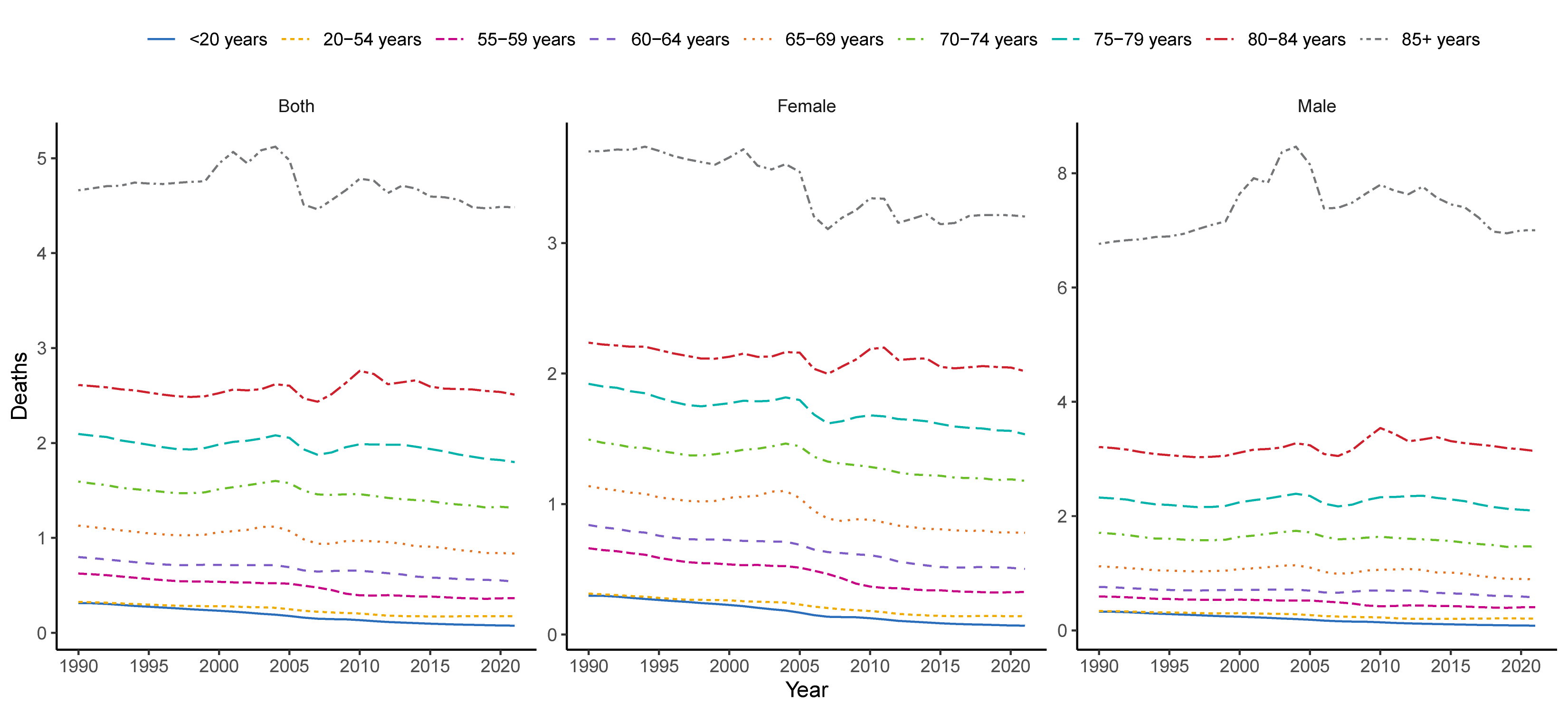

Supplement: figures and sub figures.zip [file IRNF_A_2564373_SM4374.zip › figures and sub figures/sub figures/figure4C.tif]

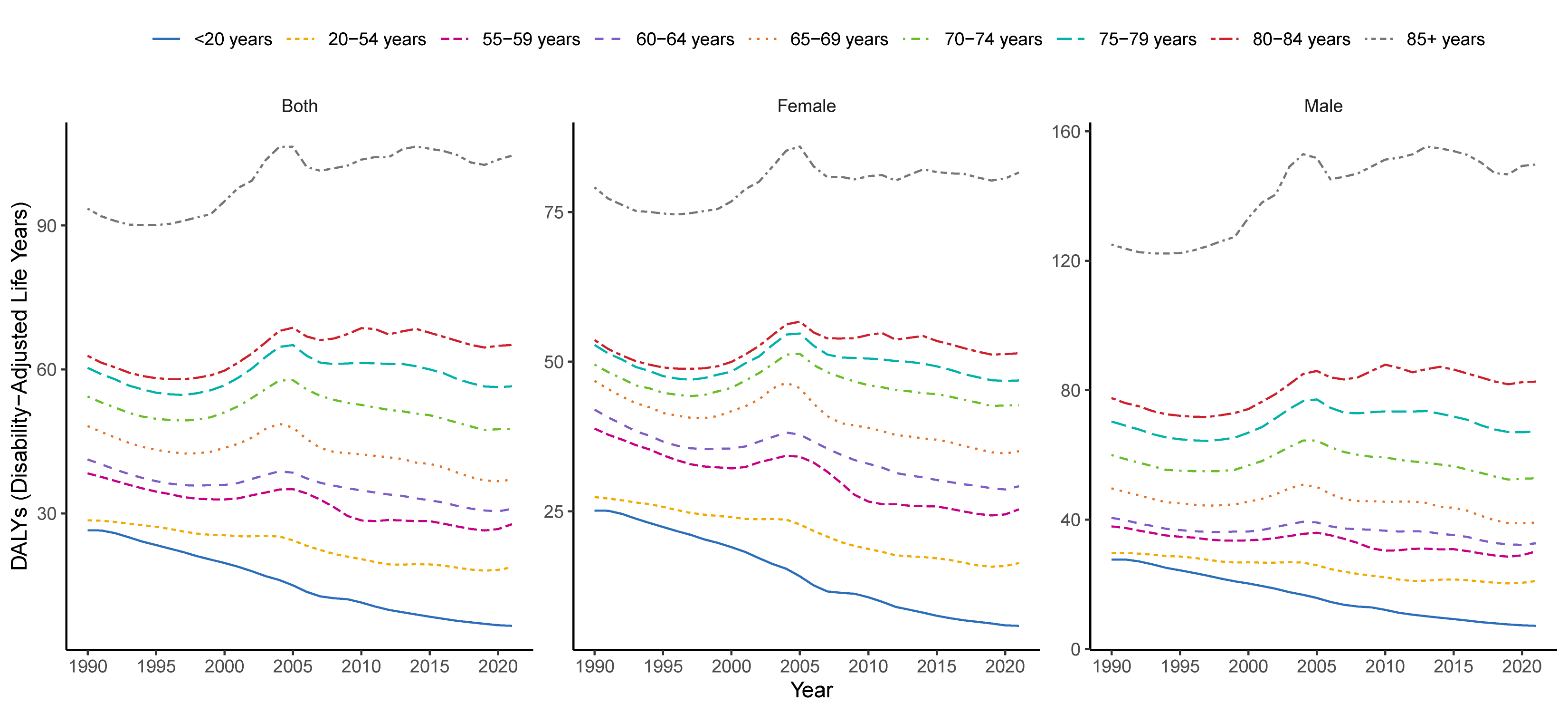

Supplement: figures and sub figures.zip [file IRNF_A_2564373_SM4374.zip › figures and sub figures/sub figures/figure4D.tif]

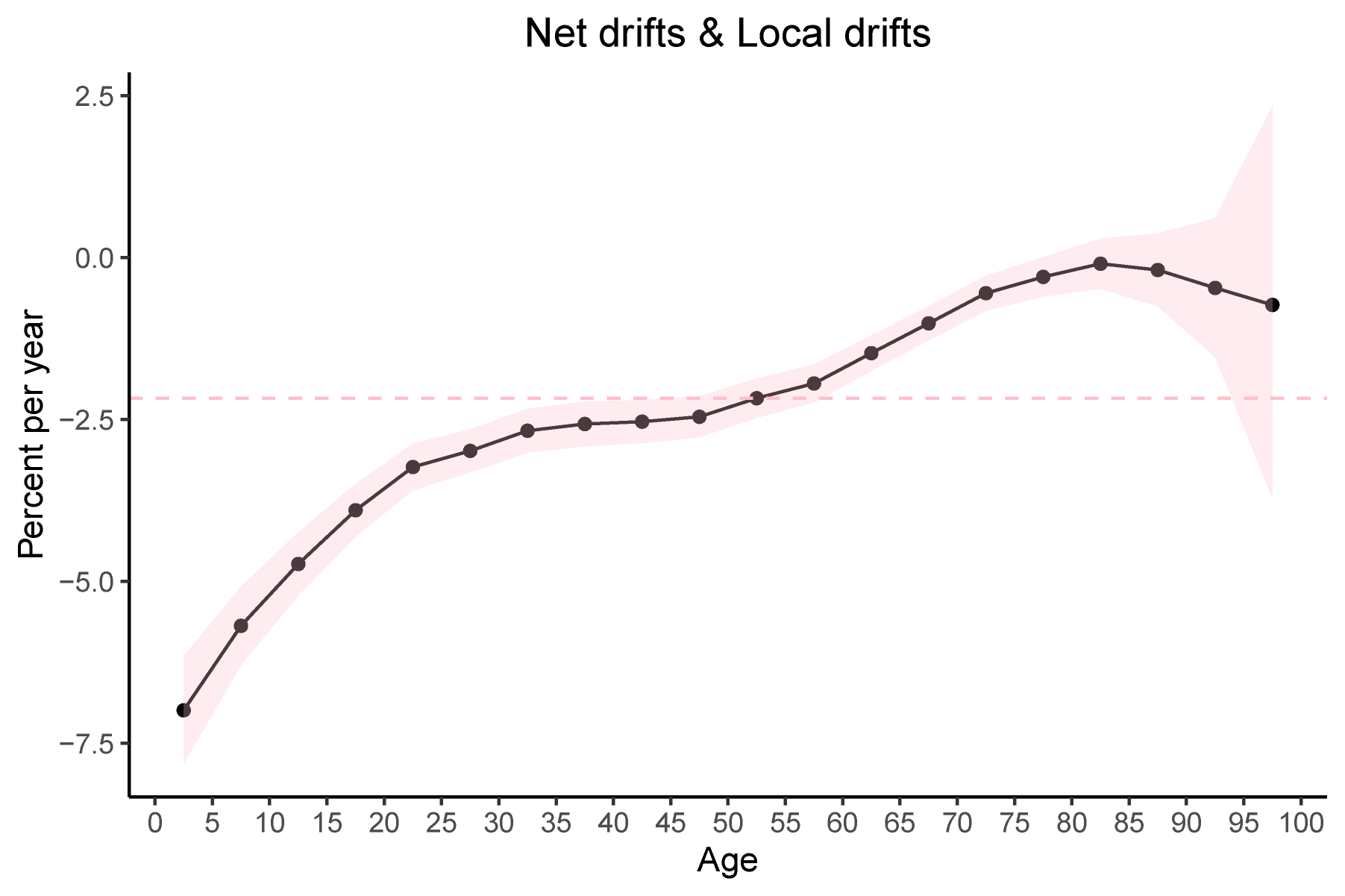

Supplement: figures and sub figures.zip [file IRNF_A_2564373_SM4374.zip › figures and sub figures/sub figures/figure5A.tif]

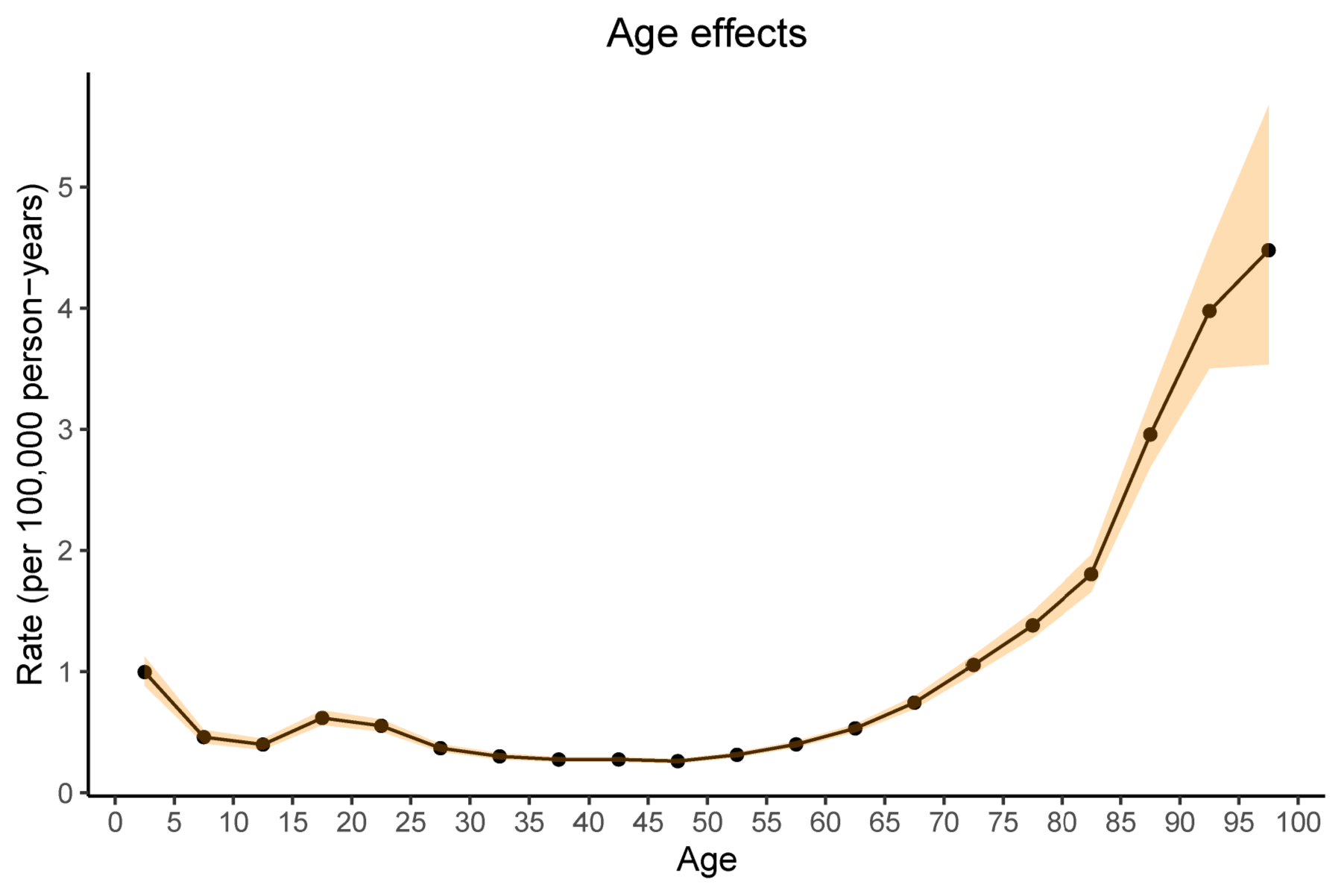

Supplement: figures and sub figures.zip [file IRNF_A_2564373_SM4374.zip › figures and sub figures/sub figures/figure5B.tif]

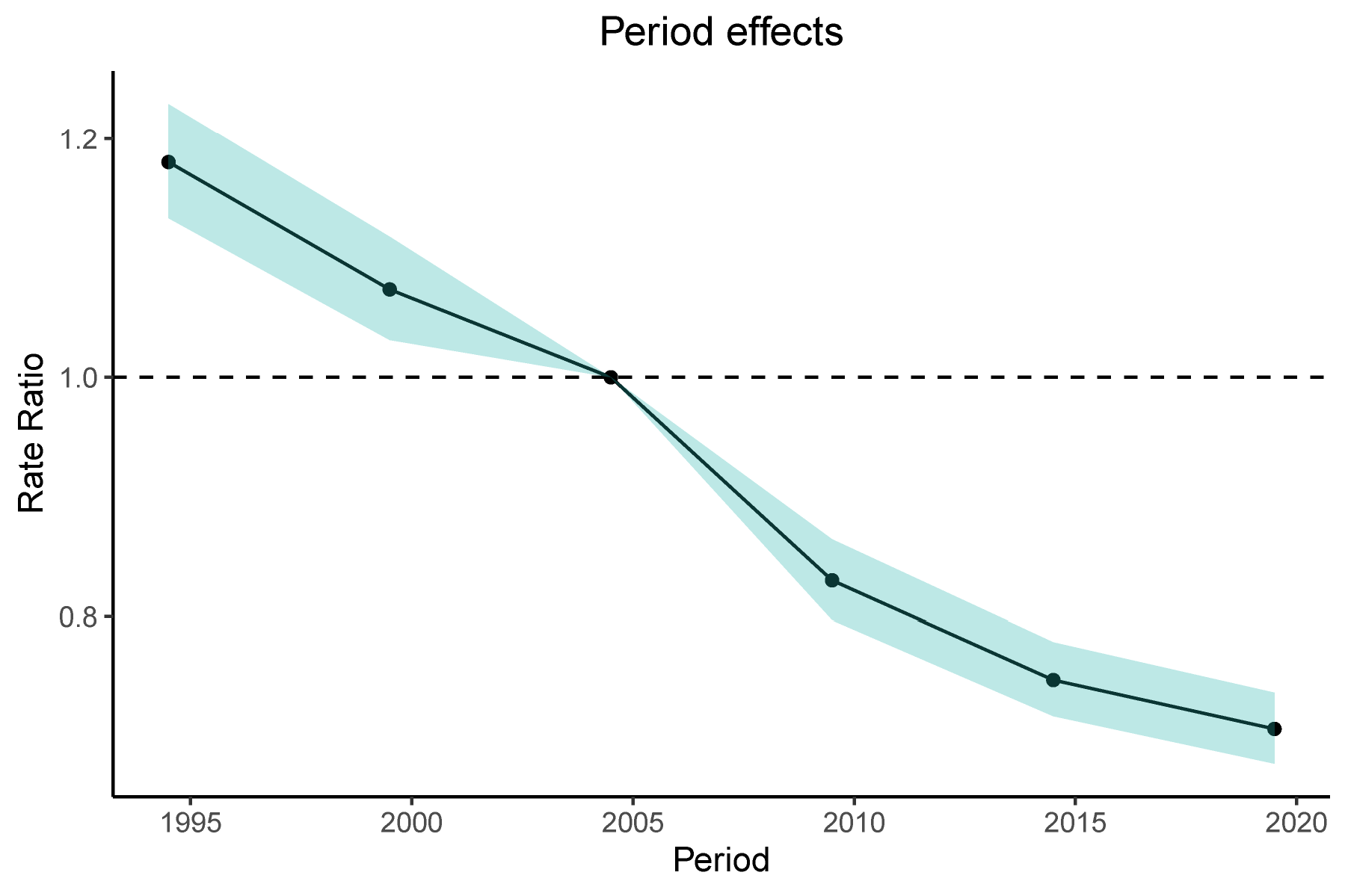

Supplement: figures and sub figures.zip [file IRNF_A_2564373_SM4374.zip › figures and sub figures/sub figures/figure5C.tif]

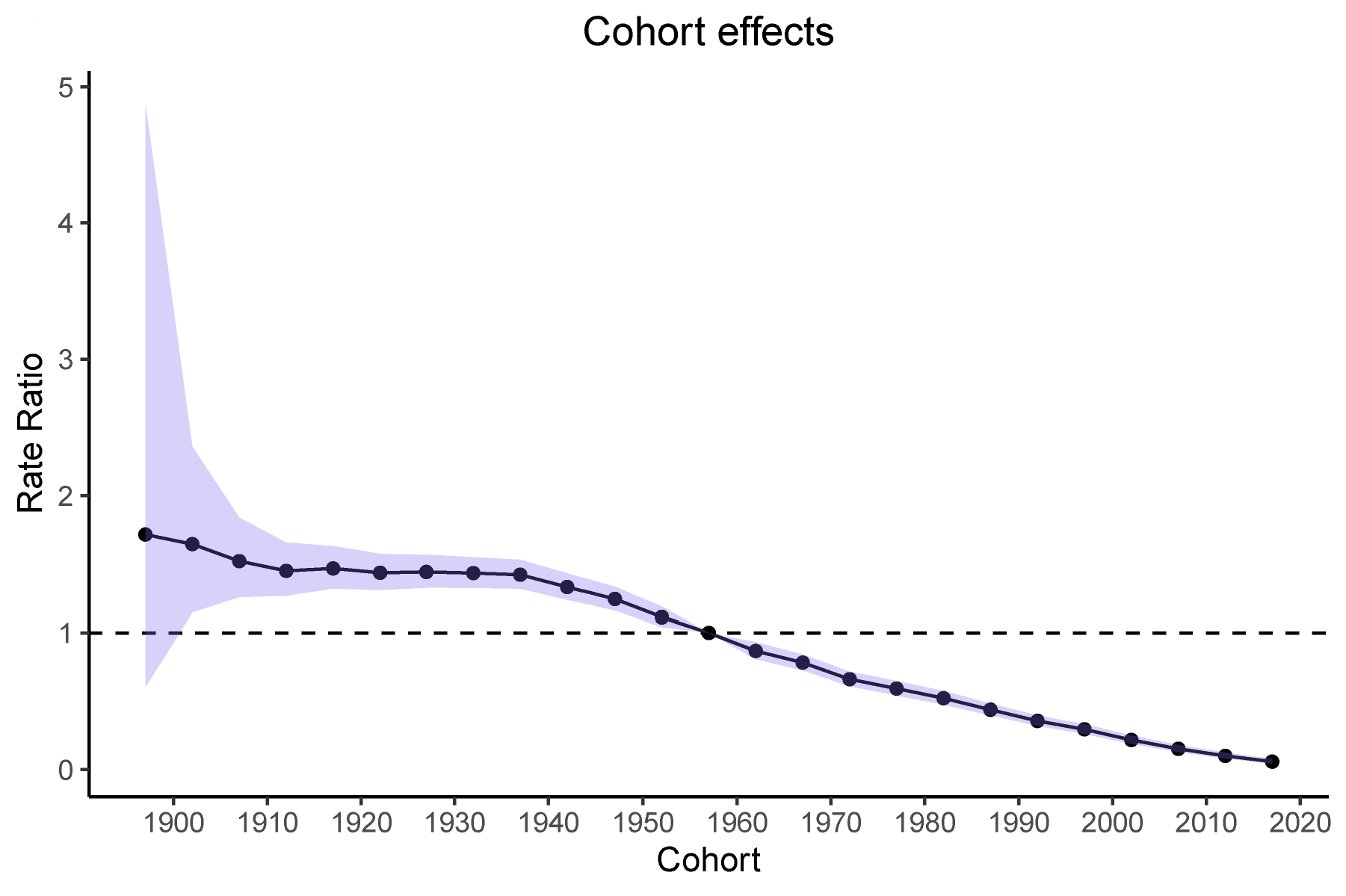

Supplement: figures and sub figures.zip [file IRNF_A_2564373_SM4374.zip › figures and sub figures/sub figures/figure5D.tif]

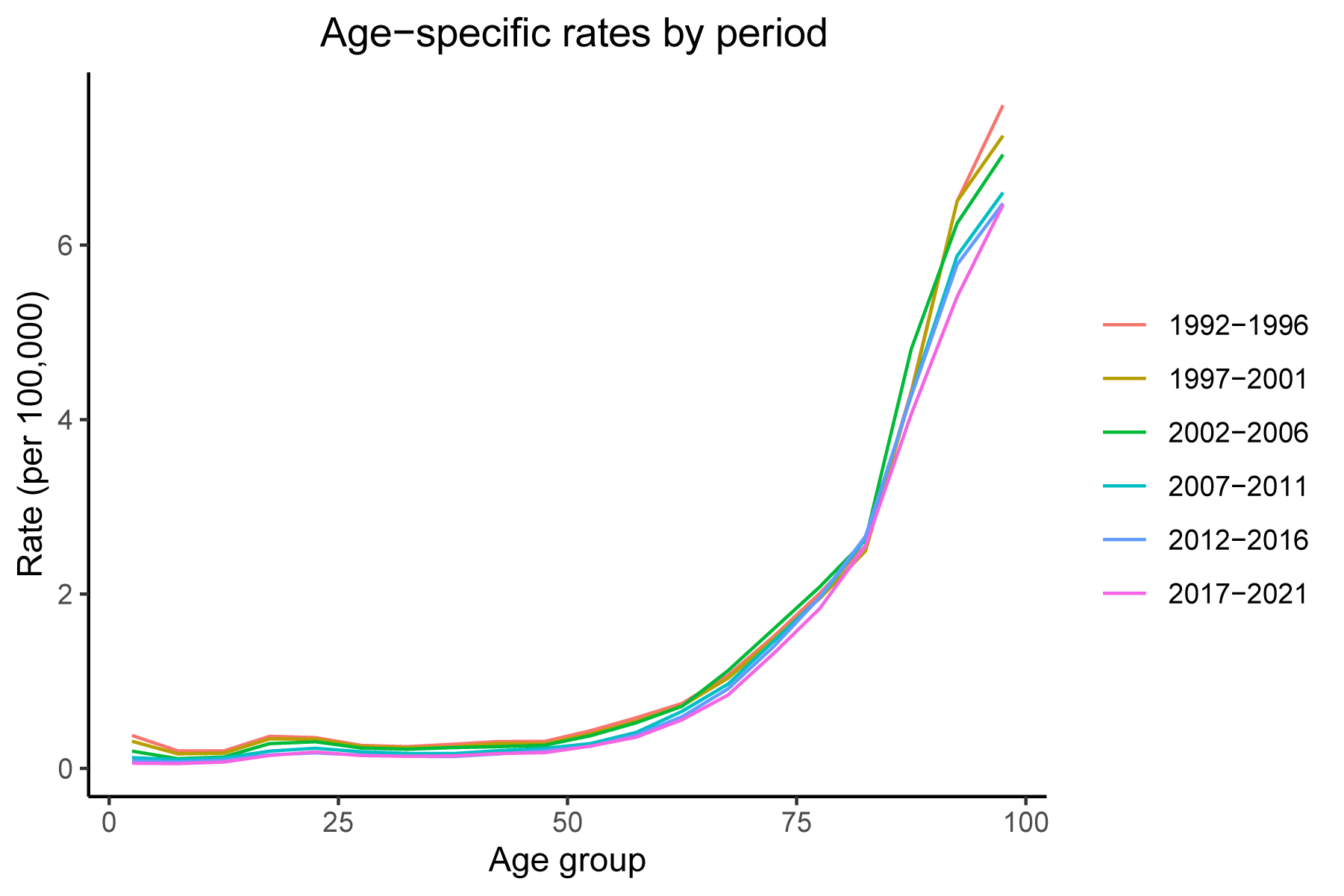

Supplement: figures and sub figures.zip [file IRNF_A_2564373_SM4374.zip › figures and sub figures/sub figures/figure5E.tif]

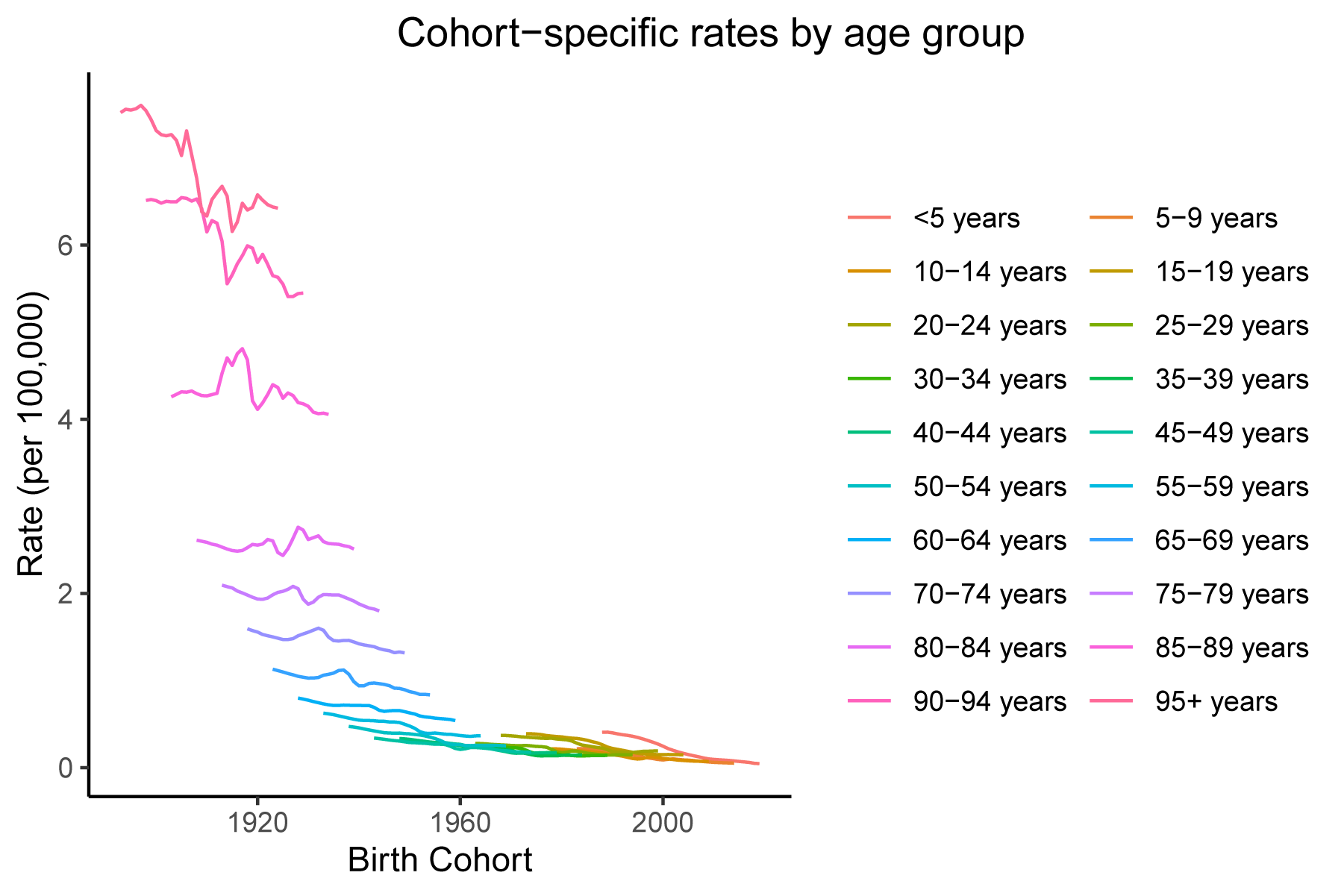

Supplement: figures and sub figures.zip [file IRNF_A_2564373_SM4374.zip › figures and sub figures/sub figures/figure5F.tif]

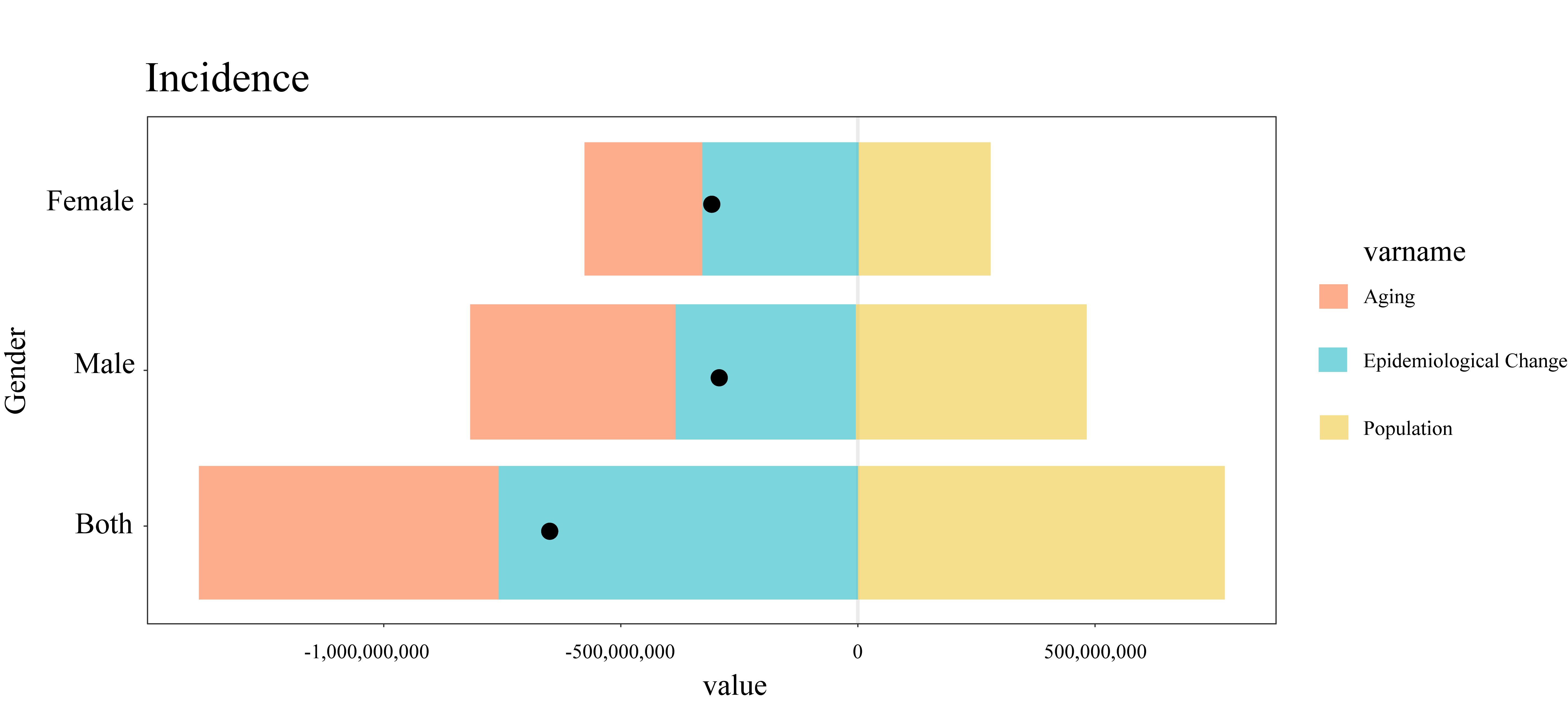

Supplement: figures and sub figures.zip [file IRNF_A_2564373_SM4374.zip › figures and sub figures/sub figures/figure6A.tif]

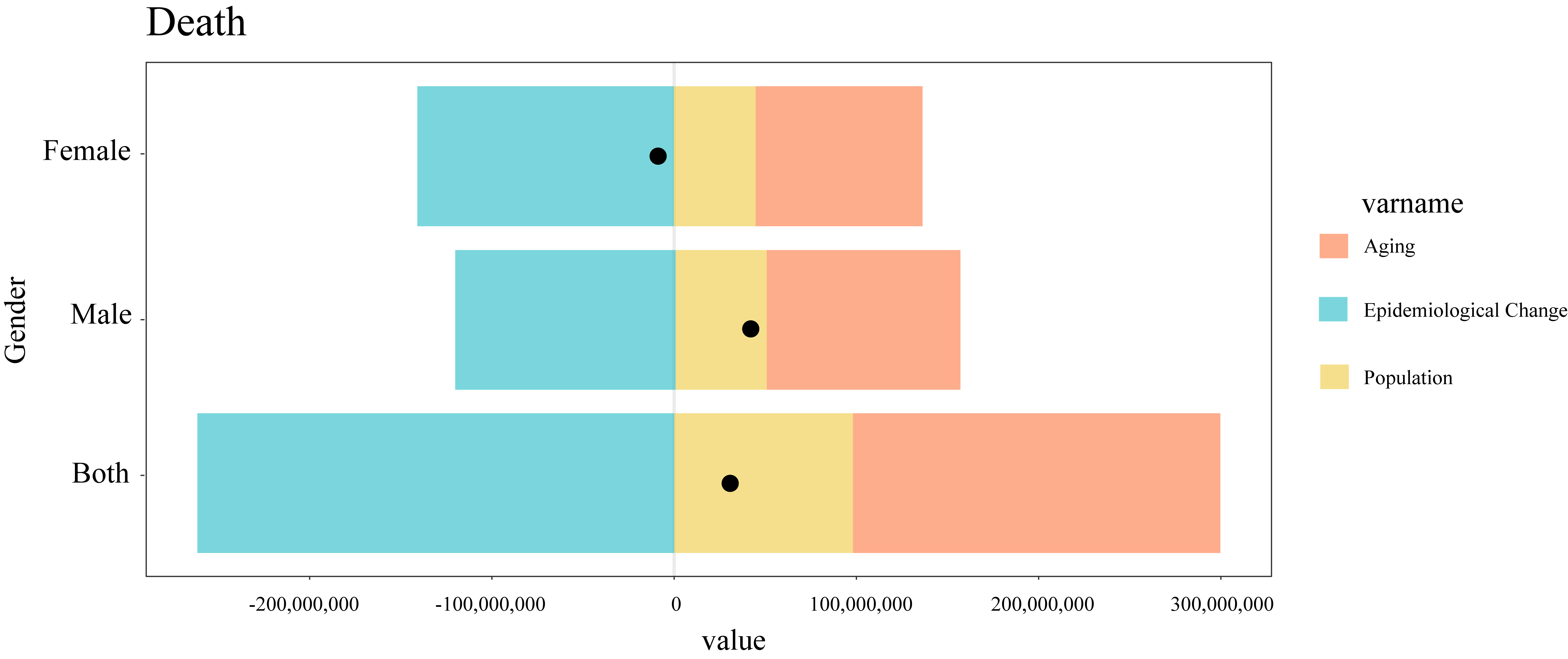

Supplement: figures and sub figures.zip [file IRNF_A_2564373_SM4374.zip › figures and sub figures/sub figures/figure6B.tif]

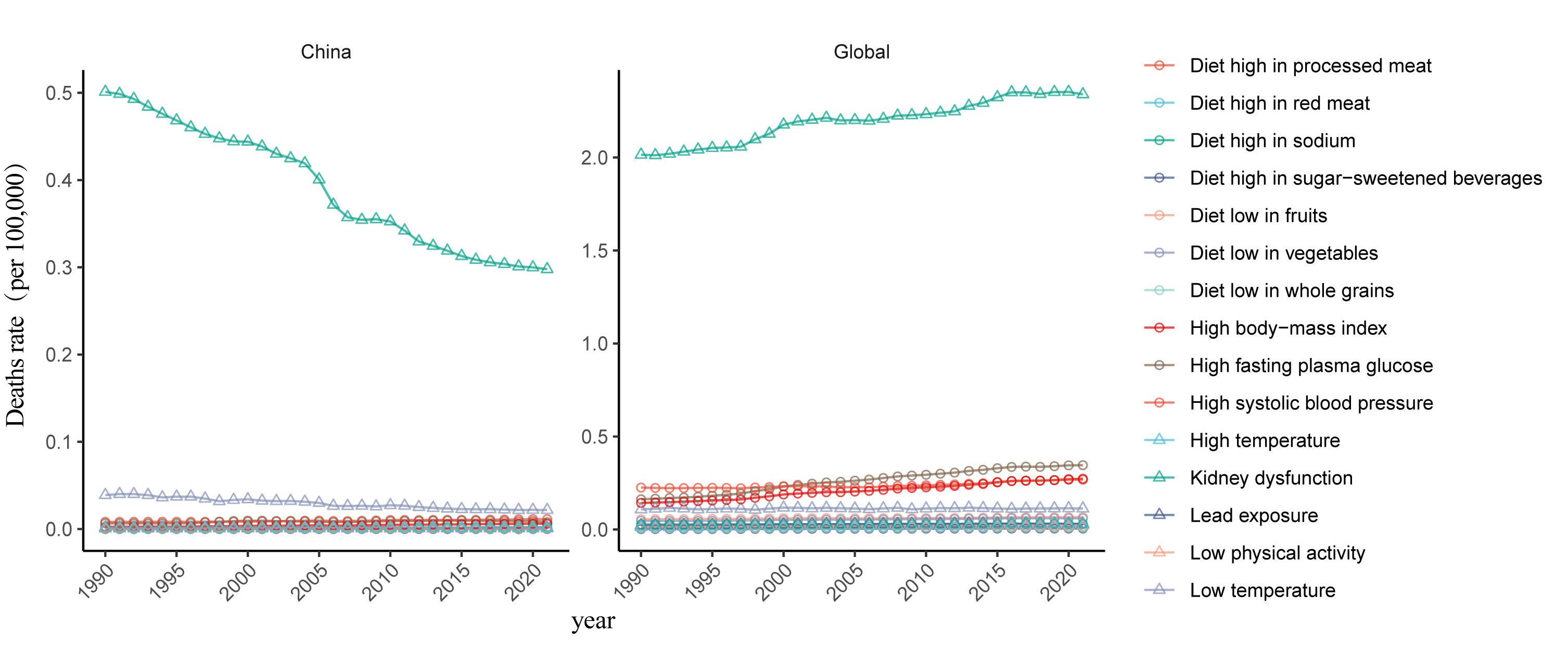

Supplement: figures and sub figures.zip [file IRNF_A_2564373_SM4374.zip › figures and sub figures/sub figures/figure8A.tif]

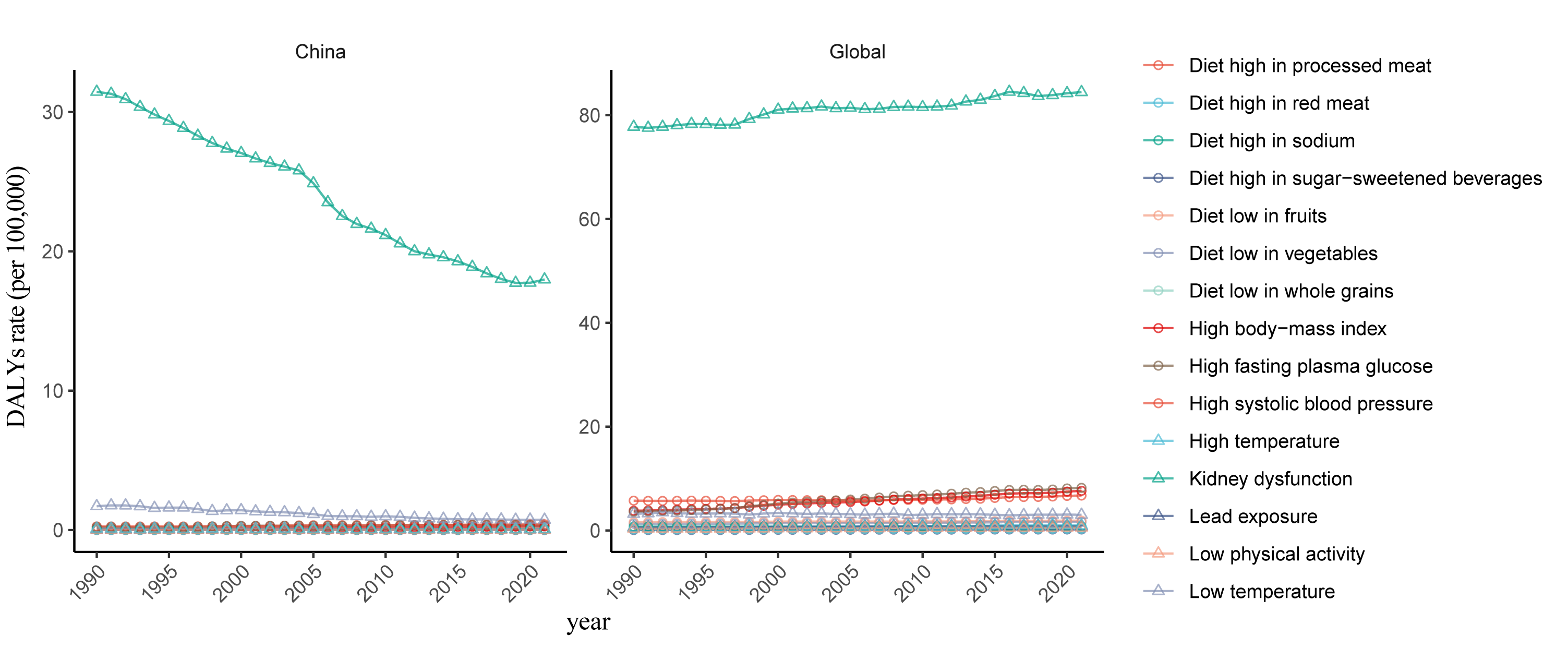

Supplement: figures and sub figures.zip [file IRNF_A_2564373_SM4374.zip › figures and sub figures/sub figures/figure8B.tif]

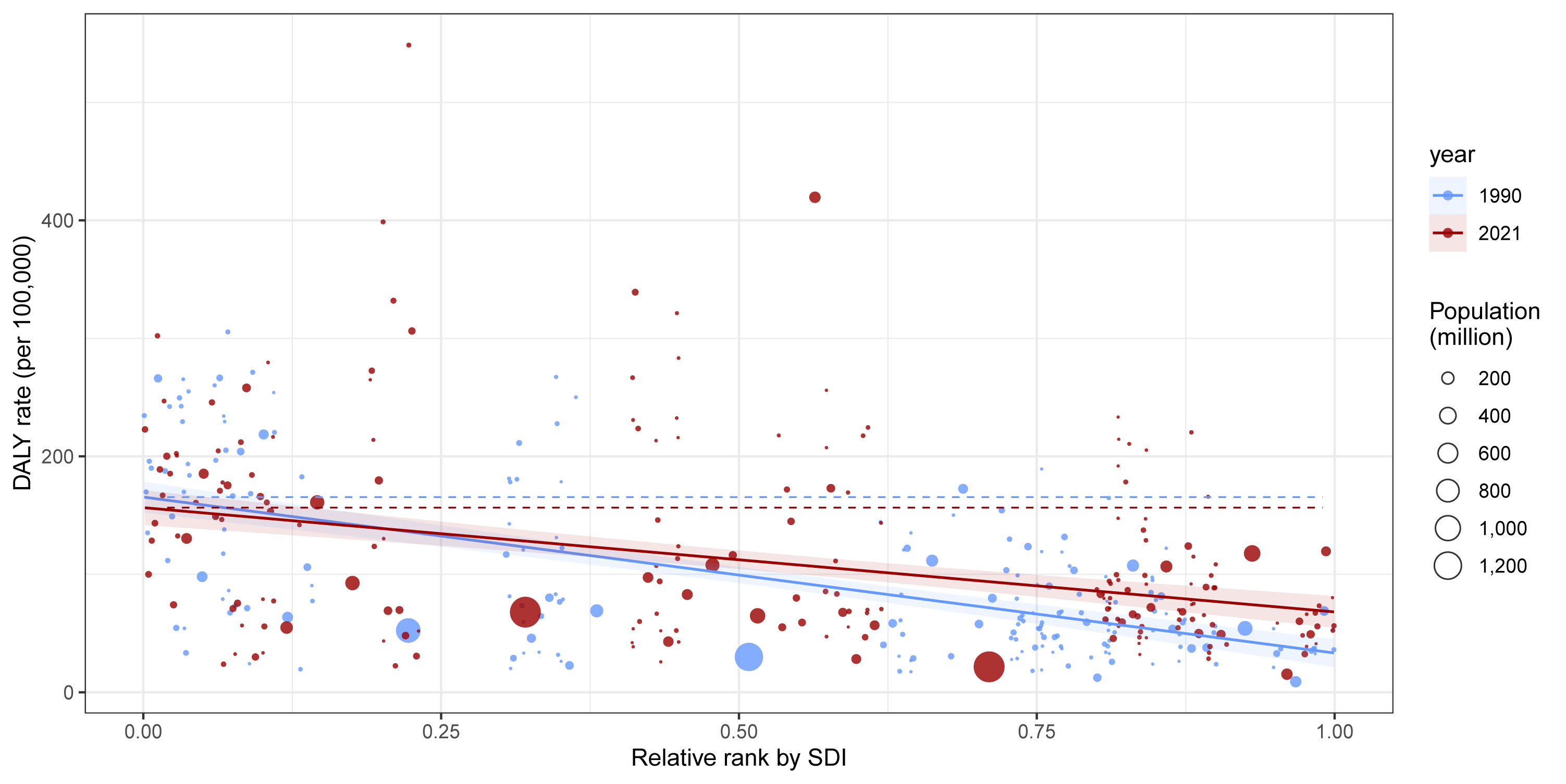

Supplement: figures and sub figures.zip [file IRNF_A_2564373_SM4374.zip › figures and sub figures/sub figures/figure9A.tif]

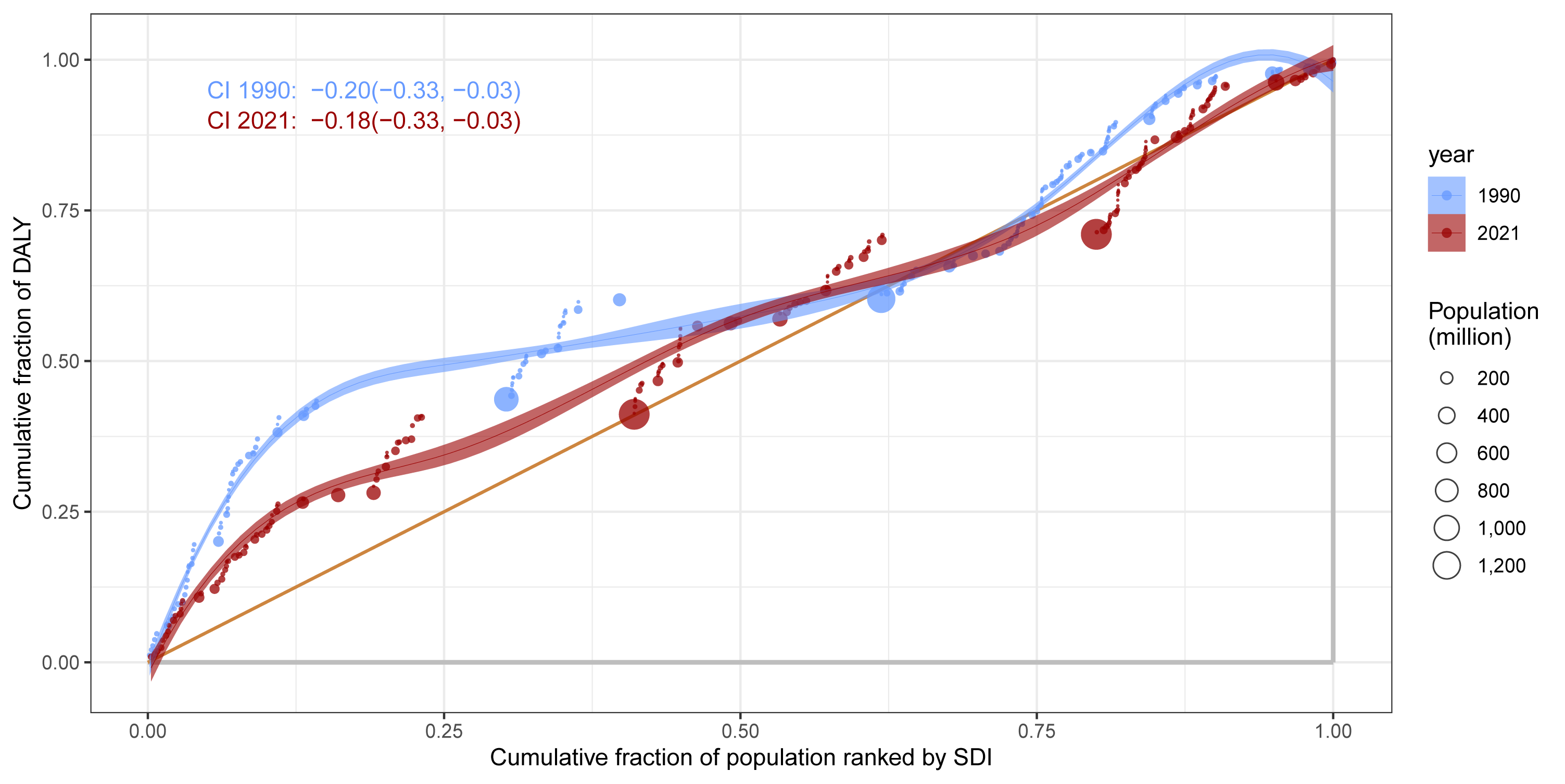

Supplement: figures and sub figures.zip [file IRNF_A_2564373_SM4374.zip › figures and sub figures/sub figures/figure9B.tif]
