# Supplementary material for: Global and China trends in glomerulonephritis-induced chronic kidney disease: health inequities, risk factors and projections to 2050
Source: Ren Fail. 2025 Oct 15;47(1):2564373. doi: 10.1080/0886022X.2025.2564373 (PMC12532362; doi:10.1080/0886022X.2025.2564373)
Supplement: Supplementary Table 1.docx [file IRNF_A_2564373_SM4372.docx]

**The diagnosis of Glomerulonephritis-induced CKD refers to ICD-10 codes (N02-N06.9)**

| **ICD10 codes** | **Detailed types** |
| --- | --- |
| N02 | Recurrent and persistent haematuria |
| N02.0 | Recurrent and persistent haematuria : minor glomerular abnormality |
| N02.1 | Recurrent and persistent haematuria : focal and segmental glomerular lesions |
| N02.2 | Recurrent and persistent haematuria : diffuse membranous glomerulonephritis |
| N02.3 | Recurrent and persistent haematuria : diffuse mesangial proliferative glomerulonephritis |
| N02.4 | Recurrent and persistent haematuria : diffuse endocapillary proliferative glomerulonephritis |
| N02.5 | Recurrent and persistent haematuria : diffuse mesangiocapillary glomerulonephritis |
| N02.6 | Recurrent and persistent haematuria : dense deposit disease |
| N02.7 | Recurrent and persistent haematuria : diffuse crescentic glomerulonephritis |
| N02.8 | Recurrent and persistent haematuria : other |
| N02.9 | Recurrent and persistent haematuria : unspecified |
| N03 | Chronic nephritic syndrome |
| N03.0 | Chronic nephritic syndrome : minor glomerular abnormality |
| N03.1 | Chronic nephritic syndrome : focal and segmental glomerular lesions |
| N03.2 | Chronic nephritic syndrome : diffuse membranous glomerulonephritis |
| N03.3 | Chronic nephritic syndrome : diffuse mesangial proliferative glomerulonephritis |
| N03.4 | Chronic nephritic syndrome : diffuse endocapillary proliferative glomerulonephritis |
| N03.5 | Chronic nephritic syndrome : diffuse mesangiocapillary glomerulonephritis |
| N03.6 | Chronic nephritic syndrome : dense deposit disease |
| N03.7 | Chronic nephritic syndrome : diffuse crescentic glomerulonephritis |
| N03.8 | Chronic nephritic syndrome : other |
| N03.9 | Chronic nephritic syndrome : unspecified |
| N04 | Nephrotic syndrome |
| N04.0 | Nephrotic syndrome : minor glomerular abnormality |
| N04.1 | Nephrotic syndrome : focal and segmental glomerular lesions |
| N04.2 | Nephrotic syndrome : diffuse membranous glomerulonephritis |
| N04.3 | Nephrotic syndrome : diffuse mesangial proliferative glomerulonephritis |
| N04.4 | Nephrotic syndrome : diffuse endocapillary proliferative glomerulonephritis |
| N04.5 | Nephrotic syndrome : diffuse mesangiocapillary glomerulonephritis |
| N04.6 | Nephrotic syndrome : dense deposit disease |
| N04.7 | Nephrotic syndrome : diffuse crescentic glomerulonephritis |
| N04.8 | Nephrotic syndrome : other |
| N04.9 | Nephrotic syndrome : unspecified |
| N05 | Unspecified nephritic syndrome |
| N05.1 | Unspecified nephritic syndrome : focal and segmental glomerular lesions |
| N05.2 | Unspecified nephritic syndrome : diffuse membranous glomerulonephritis |
| N05.3 | Unspecified nephritic syndrome : diffuse mesangial proliferative glomerulonephritis |
| N05.4 | Unspecified nephritic syndrome : diffuse endocapillary proliferative glomerulonephritis |
| N05.5 | Unspecified nephritic syndrome : diffuse mesangiocapillary glomerulonephritis |
| N05.6 | Unspecified nephritic syndrome : dense deposit disease |
| N05.7 | Unspecified nephritic syndrome : diffuse crescentic glomerulonephritis |
| N05.8 | Unspecified nephritic syndrome : other |
| N05.9 | Unspecified nephritic syndrome : unspecified |
| N06 | Isolated proteinuria with specified morphological lesion |
| N06.0 | Isolated proteinuria with specified morphological lesion : minor glomerular abnormality |
| N06.1 | Isolated proteinuria with specified morphological lesion : focal and segmental glomerular lesions |
| N06.2 | Isolated proteinuria with specified morphological lesion : diffuse membranous glomerulonephritis |
| N06.3 | Isolated proteinuria with specified morphological lesion : diffuse mesangial proliferative glomerulonephritis |
| N06.4 | Isolated proteinuria with specified morphological lesion : diffuse endocapillary proliferative glomerulonephritis |
| N06.5 | Isolated proteinuria with specified morphological lesion : diffuse mesangiocapillary glomerulonephritis |
| N06.6 | Isolated proteinuria with specified morphological lesion : dense deposit disease |
| N06.7 | Isolated proteinuria with specified morphological lesion : diffuse crescentic glomerulonephritis |
| N06.8 | Isolated proteinuria with specified morphological lesion : other |
| N06.9 | Isolated proteinuria with specified morphological lesion : unspecified |
