# Supplementary material for: Global and China trends in glomerulonephritis-induced chronic kidney disease: health inequities, risk factors and projections to 2050
Source: Ren Fail. 2025 Oct 15;47(1):2564373. doi: 10.1080/0886022X.2025.2564373 (PMC12532362; doi:10.1080/0886022X.2025.2564373)
Supplement: Supplementary Table 2.docx [file IRNF_A_2564373_SM4371.docx]

**Supplementary Table 2**: Comparative Estimated Annual Percentage Changes (EAPC) in ASRs of glomerulonephritis-induced CKD Between China and the Globe, 1990–2021

|  | **Global** |  |  | **China** |  |  |
| --- | --- | --- | --- | --- | --- | --- |
| **Measure** | **Total (95% UI)** | **Male (95% UI)** | **Female (95% UI)** | **Total (95% UI)** | **Male (95% UI)** | **Female (95% UI)** |
| **Prevalence** | 0.06(0.04, 0.08) | 0.04(0.02, 0.06) | 0.09(0.06, 0.11) | -0.39(-0.48, -0.31) | -0.48(-0.56, -0.40) | -0.28(-0.39, -0.18) |
| **Incidence** | 0.39(0.36, 0.43) | 0.45(0.41, 0.49) | 0.28(0.24, 0.32) | -0.44(-0.52, -0.36) | -0.31(-0.37, -0.24) | -0.70(-0.82, -0.58) |
| **Death** | 0.54(0.50, 0.59) | 0.43(0.39, 0.47) | 0.60(0.54, 0.66) | -1.90(-2.01, -1.79) | -1.42(-1.51, -1.33) | -2.42(-2.56, -2.27) |
| **DALYs** | 0.28(0.25, 0.31) | 0.29(0.27, 0.31) | 0.23(0.19, 0.28) | -2.05(-2.15, -1.96) | -1.76(-1.84, -1.68) | -2.38(-2.49, -2.27) |
| **YLDs** | 0.35(0.25, 0.44) | 0.35(0.25, 0.45) | 0.33(0.24, 0.41) | -0.50(-0.67, -0.34) | -0.43(-0.60, -0.26) | -0.58(-0.75, -0.40) |
| **YLLs** | 0.27(0.23, 0.31) | 0.28(0.25, 0.30) | 0.22(0.16, 0.27) | -2.95(-3.12, -2.78) | -2.52(-2.67, -2.38) | -3.45(-3.65, -3.26) |
